# Decentralisation, integration, and task-shifting in hepatitis C virus infection testing and treatment: a global systematic review and meta-analysis

**DOI:** 10.1016/S2214-109X(20)30505-2

**Published:** 2021-02-24

**Authors:** Ena Oru, Adam Trickey, Rohan Shirali, Steve Kanters, Philippa Easterbrook

**Affiliations:** a Department of Global HIV, Hepatitis and STI Programmes, World Health Organization, Geneva, Switzerland; b Population Health Sciences, University of Bristol, Bristol, UK; c Precision Xtract, Boston, MA, USA; d School of Population and Public Health, University of British Columbia, Vancouver, BC, Canada

## Abstract

**Background:**

Increasing access to hepatitis C virus (HCV) care and treatment will require simplified service delivery models. We aimed to evaluate the effects of decentralisation and integration of testing, care, and treatment with harm-reduction and other services, and task-shifting to non-specialists on outcomes across the HCV care continuum.

**Methods:**

For this systematic review and meta-analysis, we searched PubMed, Embase, WHO Global Index Medicus, and conference abstracts for studies published between Jan 1, 2008, and Feb 20, 2018, that evaluated uptake of HCV testing, linkage to care, treatment, cure assessment, and sustained virological response at 12 weeks (SVR12) in people who inject drugs, people in prisons, people living with HIV, and the general population. Randomised controlled trials, non-randomised studies, and observational studies were eligible for inclusion. Studies with a sample size of ten or less for the largest denominator were excluded. Studies were categorised according to the level of decentralisation: full (testing and treatment at same site), partial (testing at decentralised site and referral elsewhere for treatment), or none. Task-shifting was categorised as treatment by specialists or non-specialists. Data on outcomes across the HCV care continuum (linkage to care, treatment uptake, and SVR12) were pooled using random-effects meta-analysis.

**Findings:**

Our search identified 8050 reports, of which 132 met the eligibility criteria, and an additional ten reports were identified from reference citations and grey literature. Therefore, the final synthesis included 142 studies from 34 countries (20 [14%] studies from low-income and middle-income countries) and a total of 489 996 patients (239 446 [49%] from low-income and middle-income countries). Rates of linkage to care were higher with full decentralisation compared with partial or no decentralisation among people who inject drugs (full 72% [95% CI 57–85] *vs* partial 53% [38–67] *vs* none 47% [11–84]) and among people in prisons (full 94% [79–100] *vs* partial 50% [29–71]), although the CIs overlap for people who inject drugs. Similarly, treatment uptake was higher with full decentralisation compared with partial or no decentralisation (people who inject drugs: full 73% [65–80] *vs* partial 66% [55–77] *vs* none 35% [23–48]; people in prisons: full 72% [48–91] *vs* partial 39% [17–63]), although CIs overlap for full versus partial decentralisation. The results in the general population studies were more heterogeneous. SVR12 rates were high (≥90%) across different levels of decentralisation in all populations. Task-shifting of care and treatment to a non-specialist was associated with similar SVR12 rates to treatment delivered by specialists. There was a severe or critical risk of bias for 46% of studies, and heterogeneity across studies tended to be very high (*I*^2^>90%).

**Interpretation:**

Decentralisation and integration of HCV care to harm-reduction sites or primary care showed some evidence of improved access to testing, linkage to care, and treatment, and task-shifting of care and treatment to non-specialists was associated with similarly high cure rates to care delivered by specialists, across a range of populations and settings. These findings provide support for the adoption of decentralisation and task-shifting to non-specialists in national HCV programmes.

**Funding:**

Unitaid.

## Introduction

Hepatitis C virus (HCV) infection is a major cause of liver disease that leads to approximately 399 000 deaths annually.^1^ An estimated 71 million people are chronically infected, with a disproportionately high burden in low-income and middle-income countries (LMICs). In recognition of this major global public health burden, in 2016, WHO launched a Global Health Sector Strategy on Viral Hepatitis 2016–2021.^2^ This strategy outlined a set of global targets and the scale-up of five key synergistic interventions for prevention, testing, and treatment to achieve the goal of eliminating viral hepatitis as a public health threat by 2030 (ie, a reduction in hepatitis-related mortality by 65% and in incidence of chronic infections by 90%). Good progress has been made in several key LMICs, such as Egypt, Georgia, Rwanda, and Mongolia, in the scale-up of treatment access and highly effective preventive approaches to reduce transmission, such as blood and injection safety.^3^ However, we are far from achieving the 2030 coverage targets of 90% diagnosis and 80% treatment of those infected. As of 2017, only 20% of people with HCV infection had been tested and approximately a quarter of diagnosed people were treated.^4^

**Research in context**

**Evidence before this study**

We searched PubMed, Embase, WHO Global Index Medicus, WHO International Clinical Trials Registry, and conference abstracts for studies of models of hepatitis C virus (HCV) care. The search included studies in all languages published from Jan 1, 2008, to Feb 20, 2018. The following search terms were used: “hepatitis C OR HCV” AND “delivery of health care” OR “delivery of health care, integrated” OR “model of care” OR “community care” OR “primary care”. We identified eight systematic reviews that had examined different interventions aimed to increase uptake of HCV testing, linkage to care, and treatment. The studies in these systematic reviews were mostly from high-income countries, focused only on certain steps in the care cascade, and were predominantly among people who inject drugs. Many were also done before the introduction of direct-acting antivirals (DAAs) and had small sample sizes (<50 patients). Nonetheless, previous studies found evidence that strategies such as offering HCV testing and care within harm-reduction services and primary care clinics would improve uptake of care and treatment.

**Added value of this study**

To inform future WHO global recommendations on simplified service delivery for HCV care and treatment, we assessed the effects of a range of different service delivery approaches, specifically decentralisation and task-shifting, on all key outcomes along the cascade of HCV care in people who inject drugs, people in prisons, people living with HIV, and the general population. Outcomes evaluated included uptake of serological and nucleic acid testing, linkage to care, treatment, cure assessment (ie, sustained virological response at 12 weeks after the end of treatment, SVR12), and cure rate. Overall, studies that provided fully decentralised care had similar cure rates (for both interferon-based and DAA-based regimens) to those achieved at tertiary centres, but also had increased linkage to care and treatment among people who inject drugs, at sites where HCV testing and treatment were also integrated with delivery of harm-reduction services. Among people in prisons, studies with fully decentralised care had higher rates of linkage to care than those with partially decentralised care. Our study also showed that in all populations studied, task-shifting of HCV care and treatment to non-specialists achieved similarly high SVR12 cure rates to care provided by specialists.

**Implications of all the available evidence**

To meet the global targets for elimination of HCV, access to testing and treatment needs to be scaled up substantially. Our findings provide new evidence that decentralisation of testing, care, and treatment to harm-reduction sites among people who inject drugs and within prisons improves linkage to care and treatment, as well as achieving high cure rates. We also found that care delivered by non-specialists has similar outcomes to that delivered by specialists. This finding has direct implications for countries where HCV care and treatment are currently restricted to certain tertiary-care facilities or provided only by specialists (eg, hepatologists). Our results provide support for countries to expand access to their services for HCV testing, care, and treatment beyond specialist centres and providers to lower-level health facilities and to use the existing non-specialist and primary health-care workforce.

The global response and opportunities for elimination of HCV infection have been transformed by advances in treatment and diagnostics, as well as by price reductions.^3^ These advances include the introduction of curative, short-course direct-acting antiviral (DAA) therapy in 2014, and the widespread availability of rapid diagnostic testing for HCV antibody and availability of nucleic acid testing or core antigen testing for HCV viraemia. The development of evidence-based WHO guidelines for a simplified public health approach for HCV testing and treatment, and recommendations for a treat-all approach regardless of stage of disease using a few pangenotypic regimens, has provided further support for the scale-up of testing and treatment.^5^, ^6^, ^7^ However, until recently, data have been scarce and little attention has been paid to the optimal approaches to service delivery of HCV testing, care, and treatment in different settings and populations. Much of the evidence to inform simplified approaches, such as decentralisation of care to primary-care facilities and task-shifting to nurses and non-specialists, is based on the HIV literature,^8^ in which adoption of these approaches had a transformative impact on the scale-up of antiretroviral treatment.^9^ There are even greater opportunities with HCV infection compared with HIV, because care and short-course curative treatment requires minimal expertise and monitoring.

Although previous reviews of interventions to promote uptake of HCV testing and treatment have been done, to our knowledge none have comprehensively addressed the effects of decentralisation of testing and treatment beyond tertiary-level or specialist facilities to the primary-care or secondary-care level, or task-shifting of care to non-specialists, and previous reviews have not included studies from LMICs.^10^, ^11^, ^12^, ^13^, ^14^, ^15^, ^16^, ^17^ We did a systematic review and meta-analysis of published literature, grey literature, and unpublished sources to synthesise evidence on the effectiveness of these service delivery approaches. The specific interventions considered were full decentralisation of testing and treatment at the same site or partial decentralisation of testing but referral elsewhere for treatment, integration of HCV testing and treatment with harm-reduction and other services, and task-shifting of care and treatment to non-specialists. We evaluated the effects of these interventions on six key outcomes across the HCV care cascade: uptake of serological testing, uptake of viral load testing, linkage to care, treatment uptake, uptake of cure assessment, and attainment of treatment cure. These outcomes were evaluated in four populations most affected by HCV infection: people who inject drugs, people in prisons, and people living with HIV, as well as the general population.

## Methods

### Search strategy and selection criteria

We did a systematic review and meta-analysis in accordance with PRISMA guidelines. We did a comprehensive search of PubMed, Embase, and WHO Global Index Medicus for studies reporting models of HCV care. The search was done on Feb 20, 2018, and included studies in all languages published from Jan 1, 2008. A further updated search was done on April 1, 2020, to identify studies published from Feb 21, 2018, to Feb 28, 2020. Searches were tailored to the functionality of each database, but generally used the following terms: “hepatitis C OR HCV” AND “delivery of health care” OR “delivery of health care, integrated” OR “model of care” OR “community care” OR “primary care”. In addition, accepted conference abstracts from the World Hepatitis Summit (2017), the International Network on Hepatitis in Substance Users (2015–18), the European Association for the Study of the Liver (2015–18), the American Association for the Study of Liver Diseases (2015–18), and the Asian Pacific Association for the Study of the Liver (2015–18) were searched. The reference lists of all retrieved articles, as well as review articles identified during the initial search, were screened for relevant citations. Forward citation checks were done to identify any additional studies. Full details of the search strategy are provided in the appendix (p 1). A secondary search on Google search engine and the WHO International Clinical Trials Registry was done to identify existing grey literature (appendix p 1). The contacts directory of the WHO Global Hepatitis Programme was also used to solicit additional studies of hepatitis service delivery from LMICs.

Authors EO and AT did the search and independently evaluated retrieved articles, abstracts, and grey literature to determine the eligibility of the study according to the inclusion criteria. PE reviewed final selection and arbitrated when there were differences between the two primary reviewers. Citations generated by electronic scanning were assessed for relevance based on title, abstracts, and descriptor terms. We included randomised controlled trials (RCTs), non-randomised studies, and observational studies that reported interventions along the early phase of the care continuum, including serological testing, confirmatory viral load testing, and linkage to care, and the late phase of the care continuum, including treatment uptake, assessment of cure (sustained virological response at 12 weeks after completion of treatment [SVR12]), and cure rate (SVR12) for chronic HCV infection in adults (≥18 years). If necessary, data obtained from abstracts or grey literature were returned to authors for verification or provision of additional information. Studies with a sample size of ten or less for the largest denominator were excluded.

### Data analysis

Data were extracted from eligible studies and entered into preformatted spreadsheets. The following data points were extracted: study design, participant population, location or setting of testing and treatment, the role of the health providers, and the scope of care provided. If testing was reported, additional interventions used to promote testing uptake, access to confirmatory viral load testing, and linkage to care were recorded. If treatment was reported, we documented methods of staging of liver disease, and treatment regimens used. For consistency, testing uptake was recorded as the proportion of participants tested relative to all those offered testing, whereas treatment uptake was based only on participants who had undergone viral load testing, initial staging, and treatment assessment. If a single study contained comparator groups, data from both groups were recorded in the relevant intervention category (eg, full, partial, or no decentralisation). The main outcomes of interest were HCV serological and viral load testing, linkage to care, treatment, cure assessment, and SVR12.

Studies were classified according to levels of decentralisation for testing and treatment. Full decentralisation was defined as when both testing and treatment occurred at a primary health clinic or district hospital, harm-reduction site, or prison; partial decentralisation was defined as when testing occurred at a primary level facility followed by referral elsewhere for treatment; and no decentralisation was defined as when testing and treatment occurred at tertiary or specialist sites only. For studies of people who inject drugs, people in prisons, and people living with HIV, integration was defined as when HCV testing and treatment were done at harm-reduction sites, in prisons, or at HIV clinics alongside delivery of other key harm-reduction interventions, such as opiate substitution therapy (OST) or a needle-syringe exchange programme (NSP), or HIV care. Task-shifting was defined as delivery of HCV care and treatment by non-specialist physicians or nurses rather than specialists (eg, hepatologists). Among people who inject drugs, we categorised care facilities into three main groups: traditional OST facilities (often referred to as methadone maintenance therapy programmes or medication-assisted therapy programmes); NSP facilities (including drop-in centres and safe injection facilities); and primary-care clinics.

Authors EO and AT assessed the risk of bias for RCTs, non-randomised studies, and observational studies using the modified Downs and Black criteria.^18^ Outcomes were assessed for reporting bias, selection bias, internal validity bias, and external validity bias. For study quality, the STROBE guidelines for reporting of observational studies and conference abstracts were used.^19^

When interventions were sufficiently similar across different studies, binomial outcomes for effect sizes were pooled and summarised with 95% CIs, adjusted using the Wald method.^20^ Summary statistics, illustrated as forest plots for outcomes of interest, were calculated using meta-analytic methods stratified by population type, using the Freeman-Tukey correction method, and with random-effects models using the DerSimonian and Laird approach (fixed-effect models were used if heterogeneity was <20%).^21^ Heterogeneity was assessed using the *I*^2^ statistic,^21^ and publication bias assessed using Begg's test.^22^ Meta-regression was used to assess the effect of decentralisation on double arcsin-transformed outcome measures, as well as the effects of selected independent variables.^23^ We also pooled results from the subset of studies with a comparator group, including both randomised trials and non-randomised studies, and compared these with results from pooled analysis of non-comparator studies. p values obtained for comparative studies were from pairwise meta-analyses, whereas a two-sample z-test was used for non-comparative studies. Additional analyses were done to examine the effect of different types of harm-reduction service (NSP, OST, and other) on outcomes in studies of people who inject drugs; and the effect of the specific service provider (nurse, general practitioner [primary-care doctor], or specialist) on SVR12 in studies of task-shifting. Analyses were done using R version 3.6.1 and Stata version 15.1.

### Role of the funding source

The funder of the study had no role in study design, data collection, data analysis, data interpretation, or writing of the report.

## Results

**Figure 1:**
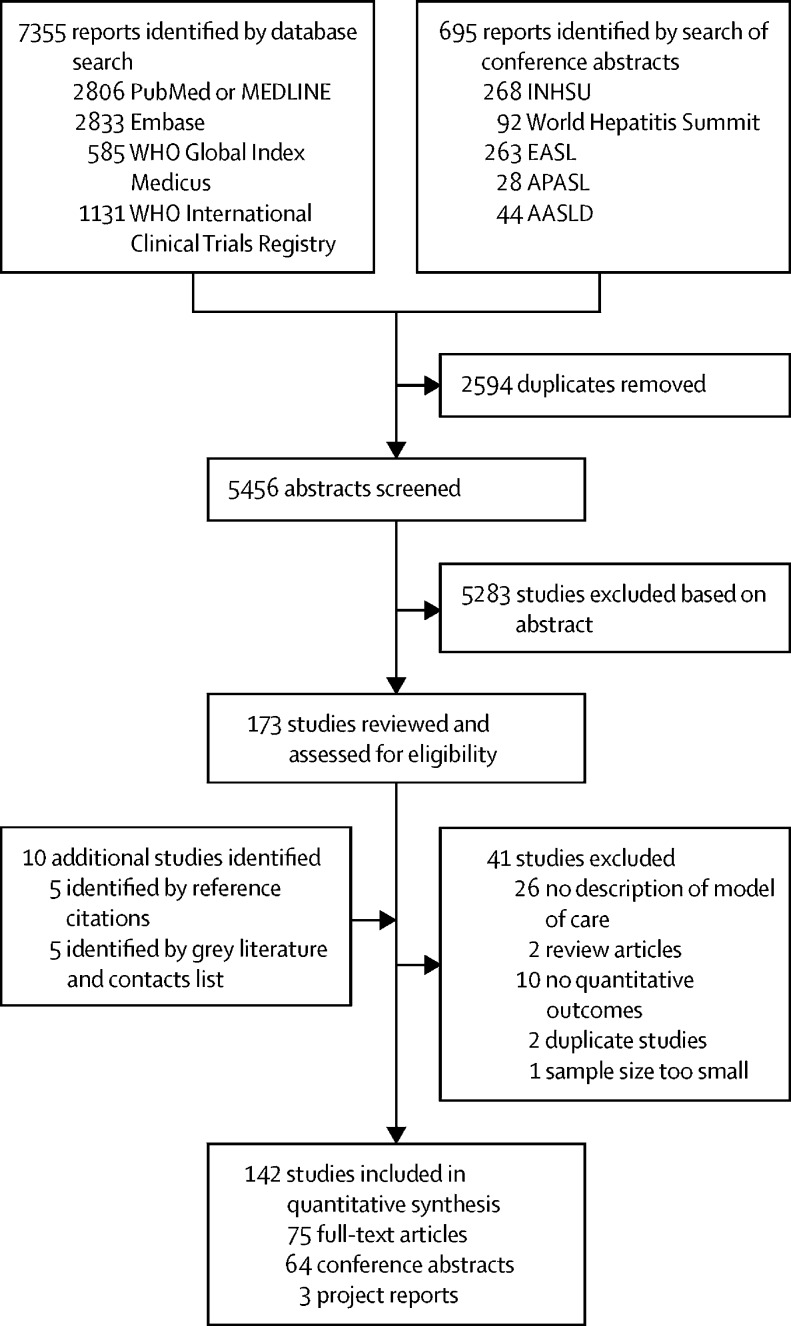
Study selection AASLD=American Association for the Study of Liver Diseases. APASL=Asian Pacific Association for the Study of the Liver. EASL=European Association for the Study of the Liver. INHSU=International Network on Hepatitis in Substance Users.

Our search identified 8050 reports, of which 5456 remained after removing duplicates (figure 1). After screening of abstracts, 5283 studies were excluded. The remaining 173 studies were reviewed and assessed for eligibility, of which 41 did not meet the eligibility criteria and were excluded. An additional ten studies were identified from reference citations and grey literature. 142 studies were included in the final synthesis, including one report with data on both people in prisons and people who inject drugs, which was counted as two separate studies. These studies included a total of 489 996 patients (239 446 [49%] from LMICs). Two additional papers identified by the updated search were not included because they did not provide any information that would have contradicted our findings on decentralisation, integration, and task-shifting.

**Figure 2:**
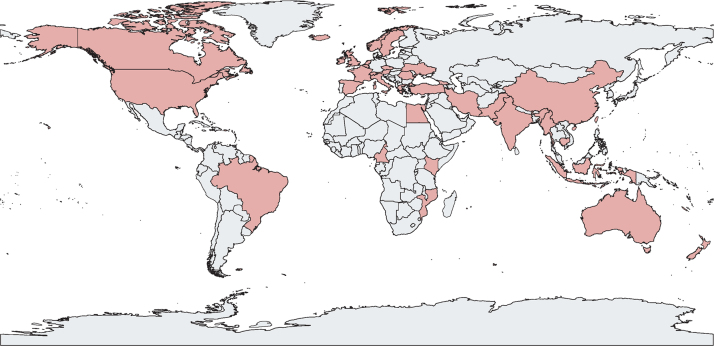
Geographical distribution of the 142 included studies Countries in which studies were done are shaded red.

Study characteristics are summarised in table 1 and the appendix (pp 2–25). 80 (56%) of 142 studies were focused on people who inject drugs, 20 (14%) on people in prisons, five (4%) on people living with HIV, and 37 (26%) on the general population. Figure 2 shows the geographical distribution of the included studies, of which 123 (87%) were from high-income countries, with the largest numbers from the USA (41 studies, 29%), Australia (27, 19%), the UK (18, 13%), and Canada (17, 12%). 20 (14%) studies were from LMICs, and included seven studies in people who inject drugs (Turkey, India, China, Kenya, Georgia, Romania, and Iran), one study in people in prisons (Ukraine), two studies in people living with HIV (Myanmar and Mozambique), and ten studies in the general population (Pakistan, Egypt, Indonesia, Cameroon, Cambodia, China, and two studies each in India and Brazil). In the 37 studies in the general population (across all income settings), care was provided in primary-care settings in ten studies and in hospital-based clinics in 27 studies. Most studies in people who inject drugs were based at harm-reduction sites offering OST (48 studies), with another 14 studies in NSPs and 18 studies in other locations, such as clinics for homeless people or drop-in centres.

**Table 1:** Summary of characteristics of 142 included studies

		**Total (142 studies)**	**People who inject drugs (80 studies, 56%)**	**General population (37 studies, 26%)**	**People in prisons (20 studies, 14%)**	**People living with HIV (5 studies, 4%)**
Low-income and middle-income countries		20 (14%)	7 (9%)	10 (27%)	1 (5%)	2 (40%)
Coverage of outcomes in care cascade						
	Early phase of cascade (testing with or without linkage)	17 (12%)	8 (10%)	2 (5%)	7 (35%)	0
	Late phase of cascade (treatment with or without linkage)	87 (61%)	53 (66%)	24 (65%)	8 (40%)	2 (40%)
	Full cascade	38 (27%)	19 (24%)	11 (30%)	5 (25%)	3 (60%)
Decentralisation*						
	Number of decentralisation study groups	154	86	41	20	7
	Full decentralisation (and integration)†	88 (57%)	55 (63%)	16 (39%)	12 (60%)	5 (71%)
	Partial decentralisation (testing at site, referral for care)	44 (29%)	25 (29%)	11 (27%)	8 (40%)	0
	No decentralisation	22 (14%)	6 (7%)	14 (34%)	0	2 (29%)
Task-shifting*						
	Number of task-shifting study groups	153	82	44	20	6
	Non-specialist treatment	46 (30%)	27 (33%)	10 (23%)	6 (30%)	3 (50%)
	Non-specialist treatment with onsite or telehealth-guided specialist support	24 (16%)	15 (18%)	4 (9%)	4 (20%)	1 (17%)
	Specialist treatment	51 (33%)	20 (24%)	25 (57%)	3 (15%)	2 (33%)
	Unknown or not applicable	32 (21%)	20 (24%)	5 (11%)	7 (35%)	0
Treatment regimen						
	DAAs	83 (58%)	50 (63%)	19 (51%)	10 (50%)	4 (80%)
	DAAs with interferon-based regimen	7 (5%)	4 (5%)	3 (8%)	0	0
	Interferon-based regimen	35 (25%)	18 (23%)	13 (35%)	3 (15%)	1 (20%)
	Not applicable	17 (12%)	8 (10%)	2 (5%)	7 (35%)	0
Study design						
	Randomised controlled trial	6 (4%)	6 (8%)	0	0	0
	Non-randomised trials	2 (1%)	1 (1%)	1 (3%)	0	0
	Comparative prospective or retrospective study	11 (8%)	2 (3%)	6 (16%)	1 (5%)	2 (40%)
	Non-comparative observational study	123 (87%)	71 (89%)	30 (81%)	19 (95%)	3 (60%)

Data are number of study groups, number of studies (%) or number of study groups (%). One study was stratified by people who inject drugs and people in prison, so it was included in this table twice. DAA=direct-acting antiviral.

* Studies that had comparator groups with different levels of decentralisation or task-shifting were included in multiple categories.

† Includes ten studies that use an embedded specialist clinic with care provided by visiting specialists.

39 (27%) studies had a low risk of bias (score ≥20), 38 (27%) studies had a moderate risk (score 15–19), 49 (35%) studies had a serious risk of bias (score 10–14), and 16 (11%) had a critically high risk of bias (score <10; appendix p 26). The overall risk of bias was rated as moderate. Among analyses with at least ten studies, evidence of publication bias was only found for higher SVR12 rate among interferon-based regimen studies (p=0·012; appendix p 27).

**Figure 3:**
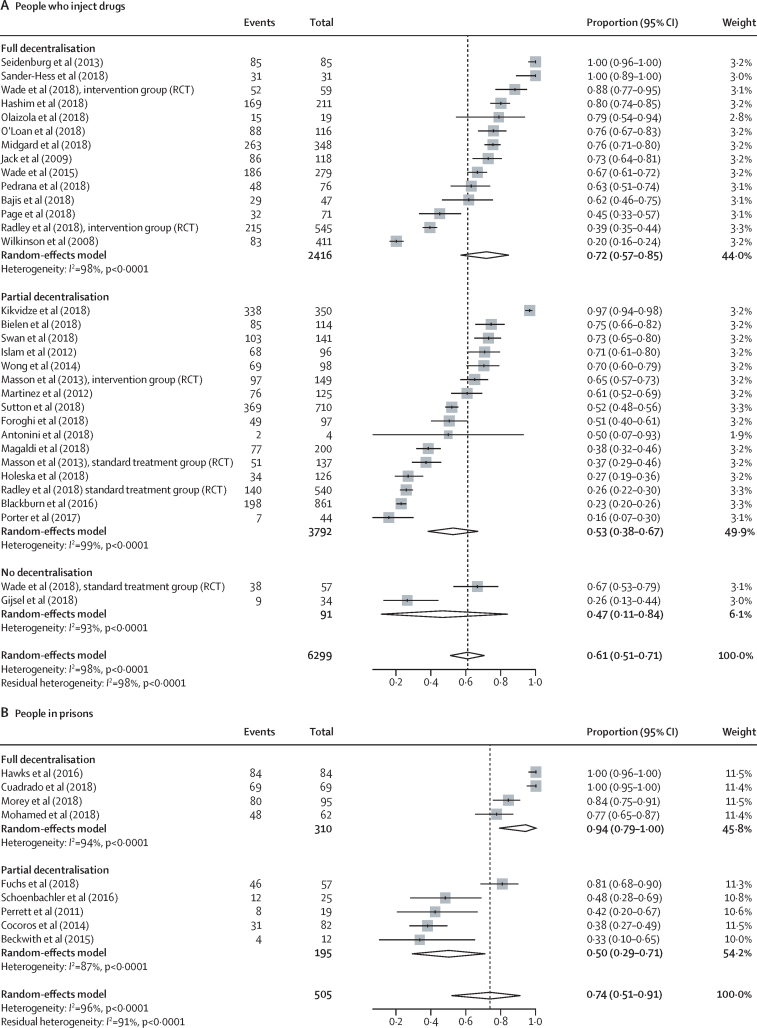

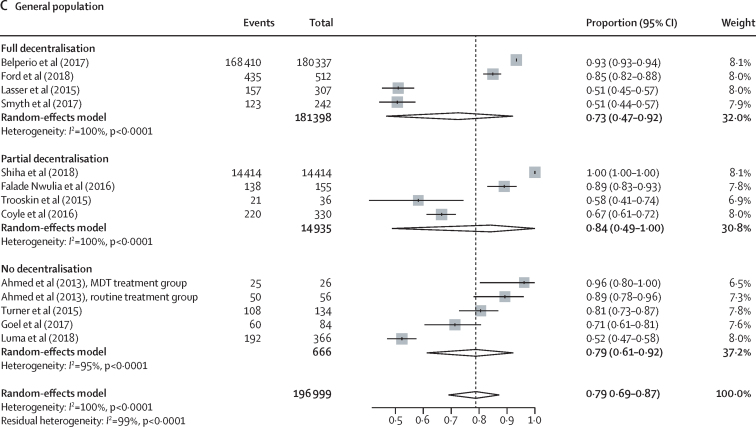
Effect of decentralisation and integration on linkage to care for people who inject drugs, people in prisons, and the general population Study details are provided in the appendix (pp 2–25). RCT=randomised controlled trial. MDT=multidisciplinary team.

**Figure 4:**
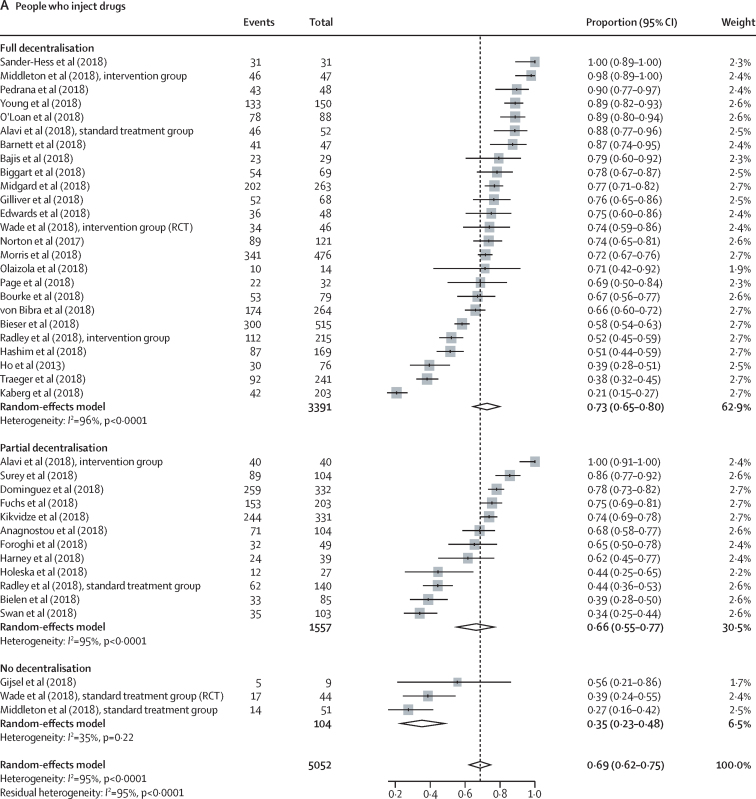

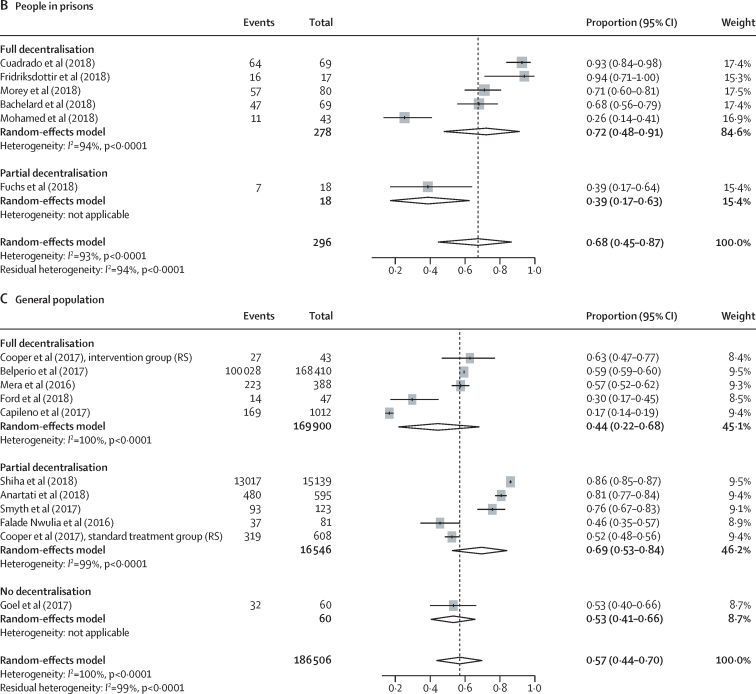
Effect of decentralisation and integration on direct-acting antiviral treatment uptake for people who inject drugs, people in prisons, and the general population Study details are provided in the appendix (pp 2–25). RCT=randomised controlled trial. RS=retrospective study.

In studies using DAAs, outcomes across the care cascade (linkage to care, treatment uptake, and SVR12) according to different levels of decentralisation among people who inject drugs, people in prisons, and the general population are presented in figure 3, figure 4, and the appendix (pp 28–40; data for studies using interferon-based regimens are presented in the appendix pp 28–38). Heterogeneity across studies in each analysis (grouped either by decentralisation or task-shifting) was generally very high. Heterogeneity of outcomes was particularly high for serological testing uptake (*I*^2^>95%), and therefore we did not report further on this outcome in the main results, and also for treatment uptake and SVR12 for interferon-based regimens. There were insufficient studies among people living with HIV for a robust examination of outcomes according to levels of decentralisation (appendix pp 41–42). Coefficients (and 95% CIs) of meta-regressions analysing differences according to levels of decentralisation for each outcome by population group are provided in the appendix (pp 49–51; analyses according to the presence or absence of task-shifting are on p 52).

Among people who inject drugs, full decentralisation of HCV testing and treatment (and when also integrated with delivery of harm reduction services [OST, NSP, or both]) showed some evidence of being associated with both increased linkage to care (full decentralisation 72% of patients linked to care [95% CI 57–85] *vs* partial decentralisation 53% [38–67] *vs* no decentralisation 47% [11–84]; figure 3A) and increased DAA treatment uptake (full decentralisation 73% [65–80] *vs* partial decentralisation 66% [55–77] *vs* no decentralisation 35% [23–48]; figure 4A), although with overlapping CIs. Based on an examination of both the estimates and CIs from the meta-analyses and the meta-regression analyses (appendix p 51), the evidence was strongest for higher levels of treatment uptake with full decentralisation versus none. There was increased nucleic acid testing uptake and linkage to care with full decentralisation versus partial or none, at harm-reduction sites providing OST or NSP, but this was statistically significant only at OST sites (appendix p 48).

Among people in prisons, there was also some evidence of increased linkage to care (full decentralisation 94% of patients linked to care [95% CI 79–100] *vs* partial 50% [29–71]; figure 3B) and increased treatment uptake (full decentralisation 72% of patients started DAA treatment [48–91] *vs* partial 39% [17–63]; figure 4B) with fully decentralised testing and treatment at the same site within a prison, compared with partial decentralisation, in which individuals were referred elsewhere for treatment after testing, although with overlapping CIs. Based on an examination of both the estimates and CIs from the meta-analyses and the meta-regression analyses (appendix p 51), there was strong evidence for increased linkage to care with full versus partial decentralisation among people in prisons.

Among the general population, the outcomes were heterogeneous, with little evidence of differences in linkage to care or DAA treatment uptake according to level of decentralisation (Figure 3, Figure 4). Similarly, in the meta-regression analyses (appendix p 51), we found no evidence of differences in outcomes according to level of decentralisation.

With use of DAA-based regimens, similar high SVR12 rates (≥90%) were achieved regardless of level of decentralisation for people who inject drugs (full decentralisation 95% [95% CI 93–97] *vs* partial 91% [86–95] *vs* none 94% [91–97]; appendix p 39), people in prisons (full 98% [96–99] *vs* partial 100% [77–100]; appendix p 40), people living with HIV (full 95% [87–99] *vs* none 100% [96–100]; appendix p 41), and the general population (full 93% [90–96] *vs* partial 94% [88–98] *vs* none 96% [92–99]; appendix p 40). In the meta-regression analyses (appendix p 52), we found no differences in DAA SVR12 rates according to level of decentralisation.

Task-shifting of care and treatment to a non-specialist was associated with similar SVR12 rates to treatment delivered by specialists using DAA-based regimens (appendix pp 43–45) in people who inject drugs (non-specialist 96% [95% CI 93–98] *vs* specialist 92% [88–96]), people in prisons (non-specialist 98% [96–99] *vs* specialist 100% [77–100]), people living with HIV (non-specialist 98% [96–99] *vs* specialist 100% [96–100]), and the general population (non-specialist 94% [90–97] *vs* specialist 94% [92–96]). Similarly, task-shifting of care and treatment was associated with similar SVR12 rates using interferon-based regimens (appendix pp 45–47). The meta-regressions for both interferon-based and DAA regimens showed no evidence of differences between specialist-led and non-specialist-led treatment (appendix p 52). The number of studies was insufficient to investigate differences between specialist and non-specialist treatment providers among people living with HIV and people in prisons.

Of 142 studies, 13 (9%) had comparator groups (RCTs, non-randomised trials, or prospective cohort studies) and had examined the effect of decentralisation of care or task-shifting of care and treatment to non-specialists. A comparison of available outcomes from this subgroup of studies using DAA regimens across groups with different levels of decentralisation (seven studies) and task-shifting (five studies), with the corresponding results for the non-comparative studies, are shown in Table 2, Table 3, Table 4 (outcomes of studies using interferon-based regimens are shown in the appendix pp 53–56). Findings were generally consistent between comparative and non-comparative studies. Among people who inject drugs, linkage to care and treatment uptake were considerably higher with full decentralisation compared with no decentralisation, with smaller increases observed for full decentralisation versus partial decentralisation (table 2). For SVR12, there were no differences by level of decentralisation or task-shifting status for any population (Table 3, Table 4). Meta-analyses of studies with comparator groups also showed similar results for full decentralisation versus partial or no decentralisation across all outcomes to those observed from the overall meta-analysis. Meta-regression analyses for outcomes according to level of decentralisation and population were limited by the lack of comparative studies, with only one study available for many of the comparisons (appendix pp 53–56). Results of the meta-regression analyses of study variables on the outcomes are presented and described in the appendix (p 49).

**Table 2:** Comparison of linkage to care, treatment uptake, and cure assessment in direct-acting antiviral regimen studies with or without comparative groups, according to population group and level of decentralisation

			**Linkage to care, % (95% CI)**				**Treatment uptake, % (95% CI)**				**Cure assessment, % (95% CI)**			
			Full decentralisation	Partial decentralisation	No decentralisation	p value	Full decentralisation	Partial decentralisation	No decentralisation	p value	Full decentralisation	Partial decentralisation	No decentralisation	p value
**People who inject drugs**														
Full decentralisation *vs* none														
	Comparative		88% (77–94)	..	67% (54–78)	0·008	88% (65–100)	..	33% (25–43)	<0·001	47% (31–64)	..	69% (44–86)	0·130
		Wade et al (2018), RCT	88% (77–94)	..	67% (54–78)	..	74% (60–85)	..	39% (26–53)	..	47% (31–64)	..	69% (44–86)	..
		Middleton et al (2018), RCS	..	..	..	..	98% (88–100)	..	27% (17–41)	..	..	..	..	..
	Non-comparative		73% (56–87)	..	26% (13–44)	<0·001	71% (62–79)	..	56% (21–86)	0·381	83% (76–90)	..	100% (98–100)	<0·001
		Number of studies	12	..	1	..	21	..	1	..	28	..	2	..
Full *vs* partial decentralisation														
	Comparative		39% (35–44)	26% (22–30)	..	<0·001	72% (32–98)	83% (10–100)	..	0·954	96% (91–99)	95% (87–98)	..	0·697
		Radley et al (2018), RCT	39% (35–44)	26% (22–30)	..	..	52% (45–59)	44% (36–53)	..	..	96% (91–99)	95% (87–98)	..	..
		Alavi et al (2018), PCS	..	..	..	..	88% (77–95)	100% (91–100)	..	..	..	..	..	..
		Cooper et al (2017), RCS	..	..	..	..	..	..	..	..	..	..	..	..
		McClure et al (2017), PCS	..	..	..	..	..	..	..	..	..	..	..	..
	Non-comparative		73% (56–87)	55% (39–70)	..	0·108	71% (62–79)	64% (53–74)	..	0·310	83% (76–90)	85% (73–94)	..	0·756
		Number of studies	12	15	..	..	21	10	..	..	28	4	..	..
**General population**														
Full *vs* partial decentralisation														
	Comparative		..	..	..	..	63% (47–77)	52% (48–57)	..	0·146	78% (73–82)	70% (50–86)	..	0·826
		Cooper et al (2017), RCS	..	..	..	..	63% (47–77)	52% (48–57)	..	..	78% (73–82)	70% (50–86)	..	..
	Non-comparative		..	..	..	..	71% (62–79)	64% (53–74)	..	0·310	86% (79–92)	89% (47–100)	..	0·829
		Number of studies	..	..	..	..	4	4	..	..	10	4	..	..

A full list of studies is provided in the appendix (pp 2–25). p values for comparative studies were from pairwise meta-analyses of dichotomous outcomes. p values for non-comparative studies were from two-sample z-tests comparing the results of meta-analyses for proportions using double arcsin transformations. RCT=randomised controlled trial. RCS=retrospective comparative study. PCS=prospective comparative study.

**Table 3:** Comparison of SVR12 outcomes in direct-acting antiviral regimen studies with or without comparative groups, according to population group and level of decentralisation

			**Full decentralisation, % (95% CI)**	**Partial decentralisation, % (95% CI)**	**No decentralisation, % (95% CI)**	**p value**
**People who inject drugs**						
Full decentralisation *vs* none						
	Comparative		100% (78–100)	..	100% (72–100)	0·688
		Wade et al (2018), RCT	100% (78–100)	..	100% (72–100)	..
		Middleton et al (2018), RCS	..	..	..	..
	Non-comparative		95% (93–97)	..	94% (90–97)	0·627
		Number of studies	28	..	3	..
Full *vs* partial decentralisation						
	Comparative		91% (84–95)	93% (90–95)	..	0·422
		Radley et al (2018), RCT	..	..	..	..
		Alavi et al (2018), PCS	..	..	..	..
		Cooper et al (2017), RCS	..	..	..	..
		McClure et al (2017), PCS	Nurse 88% (78–95); GP 93% (82–99)	93% (90–95)	..	..
	Non-comparative		95% (93–97)	90% (83–96)	..	0·150
		Number of studies	28	6	..	..
**General population**						
Full *vs* partial decentralisation						
	Comparative		95% (74–100)	95% (91–97)	..	0·780
		Cooper et al (2017), RCS	95% (74–100)	95% (91–97)	..	..
	Non-comparative		93% (90–96)	93% (86–98)	..	1·000
		Number of studies	10	4	..	..
**People living with HIV**						
Full decentralisation *vs* none						
	Comparative		94% (88–99)	..	100% (93–100)	0·867
		Doyle et al (2018), NRS	94% (88–99)	..	100% (93–100)	..
	Non-comparative		99% (97–100)	..	NA	NA
		Number of studies	3	..	0	..

A full list of studies is provided in the appendix (pp 2–25). p values for comparative studies were from pairwise meta-analyses of dichotomous outcomes. p values for non-comparative studies were from two-sample z-tests comparing the results of meta-analyses for proportions using double arcsin transformations. SVR12=sustained virological response at 12 weeks after completion of treatment. GP=general practitioner. RCT=randomised controlled trial. RCS=retrospective comparative study. PCS=prospective comparative study. NRS=non-randomised study. NA=not applicable (tests could not be performed due to a paucity of data).

**Table 4:** Comparison of SVR12 outcomes in direct-acting antiviral regimen studies with or without comparative groups, according to population group and task-shifting status

		**Non-specialist care, % (95% CI)**	**Specialist care, % (95% CI)**	**p value**
**People who inject drugs**				
Comparative		93% (86–98)	95% (92–97)	0·460
	Wade et al (2018), RCT	PCPs or nurses 100% (76–100)	100% (74–100)	..
	McClure et al (2017), PCS	Nurses 88% (78–95); GPs 93% (82–99)	93% (90–95)	..
Non-comparative		96% (94–98)	92% (86–96)	0·145
	Number of studies	22	8	..
**General population**				
Comparative		95% (92–98)	94% (91–96)	0·466
	Cooper et al (2017), RCS	Nurses 95% (74–100)	95% (91–97)	..
	Kattakuzhy et al (2017), NRS	PCPs 95% (90–98); nurses 95% (90–98)	92% (89–95)	..
Non-comparative		93% (87–97)	94% (91–97)	0·737
	Number of studies	5	9	..
**People living with HIV**				
Comparative		94% (88–98)	100% (93–100)	0·065
	Doyle et al (2018), NRS	Nurses 94% (88–98)	100% (93–100)	..
Non-comparative		99% (97–100)	NA	NA
	Number of studies	3	0	..

A full list of studies is provided in the appendix (pp 2–25). p values for comparative studies were from pairwise meta-analyses of dichotomous outcomes. p values for non-comparative studies were from two-sample z-tests comparing the results of meta-analyses for proportions using double arcsin transformations. SVR12=sustained virological response at 12 weeks after completion of treatment. RCT=randomised controlled trial. PCP=primary care physician. PCS=prospective comparative study. GP=general practitioner. RCS=retrospective comparative study. NRS=non-randomised study. NA=not applicable (tests could not be performed due to a paucity of data).

In addition to levels of decentralisation and task-shifting, other interventions were used in the various studies to promote uptake of testing. However, the use of multiple combinations of different strategies and outcomes precluded a formal quantitative analysis of their independent contribution, or attribution of an outcome to a particular intervention. We identified 11 (8%) studies that achieved high levels of coverage (>75%) for at least three steps across the care cascade (testing uptake, nucleic acid testing uptake, linkage to care, treatment uptake, and cure assessment).^24^, ^25^, ^26^, ^27^, ^28^, ^29^, ^30^, ^31^, ^32^ These studies included two in the general population;^24^, ^25^ one in people in prisons (full decentralisation);^26^ two in people living with HIV (Nguyen A, Médecins Sans Frontières, personal communication); and six in people who inject drugs (full decentralisation^27^, ^28^, ^29^, ^30^, ^31^ and partial decentralisation^32^). Across these studies, common strategies were adopted that have previously been shown to promote testing (use of rapid diagnostic tests, laboratory-based enzyme immunoassay,^28^, ^29^, ^32^ villager or peer workers,^24^ and use of dried blood spot testing^32^); uptake of nucleic acid testing (reflex laboratory nucleic acid testing,^27^, ^28^ immediate sample collection following positive rapid diagnostic test,^24^, ^25^ or use of point-of-care viral load assays); linkage to care (use of village promoters or peer workers and provision of sample transport);^24^ and treatment uptake (free treatment,^24^ peer workers,^24^ and non-specialists^25^, ^27^, ^29^, ^31^).

## Discussion

To our knowledge, this global systematic review is the most comprehensive review of service delivery models for HCV care to date. It was based on 142 studies from 34 countries, of which around a sixth were LMICs. It encompassed studies in four main affected populations: people who inject drugs, people in prisons, people living with HIV, and the general population, and examined the effects of different levels of decentralisation and task-shifting on all six key outcomes across the cascade of care.

This study has three key findings. First, we found that full decentralisation and integration of HCV testing and treatment at sites providing harm-reduction services (OST, NSP, or both, and also mental health support) for people who inject drugs and on site for people in prisons was associated with increased linkage to care and treatment. The strongest evidence was for improved treatment uptake with full decentralisation and integrated care among people who inject drugs, and for linkage to care with full decentralisation and integrated care among people in prisons. Second, similarly high rates of HCV cure (SVR12) were found in a wide range of decentralised settings for care and treatment, including harm-reduction sites, prisons, and primary-care or community settings, to those found when care was delivered in tertiary-level facilities. Third, task-shifting to non-specialist primary-care physicians or nurses was associated with similarly high levels of HCV cure using DAA regimens to those achieved by specialist-delivered care in all populations studied. Similar findings were also observed in studies using the older interferon-based regimens, as opposed to DAA regimens.

Our findings are consistent with those from the HIV literature, which show similar rates of viral suppression on antiretroviral therapy (ART), as well as increased uptake of testing and treatment, with full decentralisation (ie, community-based HIV testing and treatment at lower-level health facilities) compared with no decentralisation^15^, ^33^, ^34^, ^35^ and also with care delivered by non-specialists, including nurses, compared with care delivered by specialists.^36^, ^37^, ^38^ In our study, the effects of decentralisation were most notable among people who inject drugs and those in prisons. People who inject drugs, in particular, have difficulties accessing health services, including getting tested, linking to care, and navigating tertiary-care services.^39^ Similar to our systematic review, previous studies of ART have shown positive effects of the provision of integrated HIV care for people who inject drugs, with on-site community HIV testing and ART treatment.^40^, ^41^, ^42^ For HIV, the convenient co-location of testing and treatment integrated within OST services or primary care or community sites, where multiple needs can be met in one accessible setting, has been an important facilitator of access to care for people who inject drugs.^8^, ^41^, ^42^, ^43^ WHO currently recommends offering HIV care and ART at OST sites.^8^, ^42^ Our study findings regarding HCV support similar integration of HCV testing and treatment, and also provide new evidence that integration and co-location of HCV care and treatment can be effective not only at OST sites, but also at NSP sites.

There was considerable heterogeneity in outcomes (especially for testing uptake) across populations, care settings, treatment regimen, and service delivery interventions. However, SVR12 results for DAAs were consistently high (>90%) across all populations, care settings (eg, OST or NSP sites, prisons, primary-care clinics), and types of care provider (eg, nurses, general practitioners, addiction specialists, hepatologists), as well as across different levels of decentralised and integrated care. These findings are consistent with reports of successful treatment outcomes among people who inject drugs, including those with recent drug use and those receiving OST.^44^ We found the strongest evidence for full decentralisation was among people who inject drugs and people in prisons, but differences by level of decentralisation to primary care were not observed among studies in the general population, for which results were highly heterogeneous. This discrepancy is probably due to the currently more well established models for delivery of HCV care in OST and NSP sites in high-income countries as compared with the diverse care models for delivery of HCV care in primary-care settings for the general population.

Key strengths of this systematic review were the large number of studies included, of which around a sixth were from LMICs, and the inclusion of additional studies through searches of conference presentations and grey literature. Our updated search from 2018 to 2020 yielded only two potentially eligible studies that were not included. We also did a comprehensive analysis of the effects of interventions across all six outcomes along the cascade of HCV care. The inclusion of single-arm observational studies alongside comparative studies considerably expanded the evidence base from LMICs, and it also enabled an additional comparison of findings between studies with comparator groups and non-comparative studies. A high proportion of our studies were based on DAAs, reflecting current treatment practice.

There were several limitations to this systematic review. First, the meta-analysis was based largely on single-arm observational studies. Few studies (13, 9%) had a comparator group enabling a direct comparison of different levels of decentralisation or task-shifting, and of these only four were RCTs (there were six RCTs in total). This may introduce considerable selection bias and result in an overestimate of the effects of the interventions. Second, studies from LMICs were still under-represented, and there was a relative paucity of data on the early testing and linkage part of the cascade compared with the later treatment steps in the cascade. In addition, the use of terminology such as decentralisation, decentralised care, or task-shifting is still infrequent in the HCV literature, and it is possible that, as a result, some relevant studies were overlooked in our search strategy. Third, many included studies used several interventions targeting different steps in the care cascade, such as strategies to promote uptake and rate of serological testing (eg, opt-out testing, peer and outreach workers, electronic medical record and clinician reminders, and rapid diagnostic tests), HCV RNA confirmation (eg, reflex laboratory testing, immediate sample collection, or point-of-care viral load instruments such as GeneXpert), and linkage to care (eg, use of peer workers or patient navigators). Of note, ten studies that reported consistently high levels of coverage (>75%) across the care cascade had, in addition to full decentralisation, adopted several other well established interventions to promote access to testing and treatment. The use of multiple combinations of different strategies and outcomes precluded a formal quantitative analysis of their independent contributions, and we were unable to attribute an outcome to a particular intervention. Fourth, financial or socioeconomic factors were not reported that might have adversely affected engagement and impact on specific outcomes such as linkage rate and treatment uptake, especially in people who inject drugs or people in prisons, as well as people who are homeless.

This systematic review highlights the need for more methodologically rigorous comparative studies on packages of different interventions to promote the uptake of HCV testing, and linkage to assessment and treatment uptake, especially in LMICs. We identified few studies of different models of fully decentralised HCV testing, care, and treatment among the general population in primary care, but evidence from ongoing studies is likely to be available over the next 2 years, when an updated review would be helpful. Future studies should provide a full description of the service delivery model, and evaluation should capture effectiveness of interventions across the entire continuum of care and not just the treatment-related outcomes. Including costs would allow for comparative cost-effectiveness analyses. Additional and new strategies to promote testing and treatment access in LMICs that are currently being evaluated include the strategic use of point-of-care multi-platform technologies for measurement of HCV viral load such as GeneXpert, electronic medical record prompts,^45^, ^46^ use of mobile outreach services, especially in homeless populations,^47^, ^48^, ^49^ pharmacy-led testing and treatment initiation,^50^, ^51^ and telementoring to support non-specialists.^52^, ^53^

This systematic review has several major policy and clinical management implications for the scale-up of testing and treatment that is needed to achieve global HCV elimination targets. The WHO 2017 testing guidelines already recommend the adoption of several strategies to enhance uptake of hepatitis testing and linkage to care, including peer workers, clinician reminders for testing, and provision of testing as part of integrated services, based on a 2016 systematic review.^5^, ^17^ Similarly, the WHO 2018 guidelines for HCV infection promoted eight good-practice principles for simplified service delivery, including decentralisation, integration, and task-shifting.^7^ Our systematic review now provides a robust evidence base to support global policy and national guidelines for expanding HCV services beyond tertiary or specialist facilities to fully decentralised, co-located HCV testing and treatment at the primary-care and secondary-care level, harm-reduction services, prisons, and HIV clinics. We also show evidence for the effectiveness of task-shifting of HCV care and treatment to primary-care physicians and nurses, which should be supported by training and mentorship.^6^ Compared with HIV, there are even greater opportunities for decentralisation and task-shifting of HCV care and treatment because of the simplicity of short-course, curative, pangenotypic DAA regimens with minimal side-effects. A 2018 systematic review showed favourable SVR12 cure rates among people receiving OST and those with recent drug use.^54^ Of note, our study shows the feasibility of improving access to diagnosis and treatment among people who inject drugs and people in prisons, who are some of the most difficult populations to reach, which is critical to achieve elimination targets and reduce prevalence and ongoing transmission of HCV.

## Data sharing

The full search strategy and results used to generate data that inform the conclusion of this systematic review can be found in the appendix.
