# Supplementary material for: Decentralisation, integration, and task-shifting in hepatitis C virus infection testing and treatment: a global systematic review and meta-analysis
Source: Lancet Glob Health. 2021 Feb 24;9(4):e431–45. doi: 10.1016/S2214-109X(20)30505-2 (PMC7966682; doi:10.1016/S2214-109X(20)30505-2)
Supplement: Supplementary appendix [file mmc1.pdf]

THE LANCET
Global Health

Supplementary appendix

This appendix formed part of the original submission and has been peer reviewed. We post it as supplied by the authors.

Supplement to: Oru E, Trickey A, Shirali R, Kanters S, Easterbrook P. Decentralisation, integration, and task-shifting in hepatitis C virus infection testing and treatment: a global systematic review and meta-analysis. *Lancet Glob Health* 2021; published online Feb 24. http://dx.doi.org/10.1016/S2214-109X(20)30505-2.

Decentralization, integration, and task-shifting in hepatitis C virus infection testing and treatment: a global systematic review and meta-analysis

Supplementary materials: Figures and Tables

Search Strategy: Published Literature

Database Search: The search was carried out on 20th February 2018 on citations from January 1 2008 to February 20 2018. The search utilized the following terms "Hepatitis C OR HCV" AND "Delivery of Health Care" OR "model of care" OR "community care" OR "primary care" OR "Integrated care" as outlined below.

PubMed: (Hepatitis C" [Mesh] OR "hep c [TW] OR "hepatitis C" [Tw]) AND "last 10 years"[PDat]) AND ("Delivery of Health Care"[Mesh] OR "model of care" [TW]) AND "last 10 years"[PDat]) AND AND "last 10 years" [PDat]) OR ("Primary Health Care"[Mesh] OR "Community care"[TW] OR "Integrated care" [TW] AND "last 10 years"[PDat]). Hits: 2806

Global Index Medicus: (tw:(Delivery of healthcare)) OR (tw:(Integrated care)) OR (tw:(Primary healthcare)) OR (tw:(Community care)) AND (tw:(Hepatitis C)) AND (tw:(10 years)) Hits: 585

Embase: MeSH ((("Chronic Hepatitis C" AND "Healthcare delivery OR Community Care OR Integrated Care OR Primary Health Care OR Tertiary Health Care"))) Limit yr. "2008-current" Hits: 2833

Search Strategy: Unpublished and Grey Literature

Google Web Search: A review of the grey literature on models of service delivery models for HCV in LMICs was performed using the following indexed terms "Hepatitis C Service delivery in resource limited settings" "Hepatitis C program in low and middle-income countries". For Google Web, the first two hundred and fifty returns including presentations, reports, project summaries, flyers or posters of each search were retrieved and reviewed. Search related terms including country programs for hepatitis B and C care were also reviewed. (Search date Feb 26, 2018)

International Clinical Trials Registry: Search terminology included "Hepatitis C AND Testing", "Hepatitis C AND Treatment" AND "Hepatitis C AND Delivery of Care". Hits: 1131

Supplementary table 1: Characteristics of 142 included studies

A. People Who Inject Drugs (PWID)

Full Decentralization and Integration at sites of harm reduction								
Study	Year	Country/ Region	Setting(s)	Scope of care extracted	Study Design	Key Interventions	N	Reported Outcomes
Hashim (Project ITTREAT)[1]	2018	UK, Brighton	OST	Testing, Linkage to Care, Treatment (IFN/DAA)	Retrospective cohort of Community based testing and treatment service.	• Testing: Lab EIA (DBS), subsequent reflex HCV VL test<br>• Nurse led treatment,<br>• Other features: Onsite specialist support. Peer mentor involvement.	n= 485	Testing uptake- 97% (472/485)<br>NAT uptake- 96% (262/272)<br>Linkage rate- 80% (169/211)<br>Treatment uptake- 51% (8/169)<br>Cure assessment- 91% (79/87)<br>SVR - 87% (69/79)
Schulkind (Eradicate C study)[2]	2018	UK, Dundee	NSP	Treatment (IFN/DAA)	Prospective observational study of testing and treatment for PWID in NSPs.	• Testing: Lab EIA (DBS)<br>• Nurse led treatment,<br>• Other features: Follow up for counselling and HCV VL test at 6- and 18-months post SVR	n= 94	Testing uptake- not reported<br>NAT uptake- not reported<br>Linkage rate- not reported<br>Treatment uptake- not reported<br>Cure assessment- 100% (94/94)<br>SVR- 82% (77/94)<br>[Reinfection rate: 6 months- 5%, 18 months- 19%]
Wade (PRIME study)[*3]	2018	Australia/ New Zealand	Primary care clinic offering needle exchange, OST	Treatment (DAA)	Randomized controlled trial of HCV treatment in primary care clinic (intervention) or tertiary hospital (standardized arm)	• Testing: not described<br>• Treatment: PCP and nurse led, with specialist oversight available offsite	n= 70 (Int. arm); n= 66 (Stan. arm)	Testing uptake- not reported<br>NAT uptake- not reported<br>Linkage rate- not reported<br>Treatment uptake- Int. arm- 75% (43/57), Stan. arm- 34% (18/53)<br>Cure assessment- not reported<br>SVR- Int. arm- 49% (28/57) Stan. arm- 30% (16/53)
Ramers[4]	2018	USA, San Diego	Primary care clinic offering needle exchange	Treatment (DAA)	Prospective study on testing for HCV at NSP, OST clinic and referral to primary care clinic for staging and treatment.	• Testing: RDTs, followed immediately by phlebotomy for HCV VL testing.<br>• Treatment: PCP led.<br>• Other features: Referrals supported by patient navigator	n= 193	Testing uptake- not reported<br>NAT uptake- not reported<br>Linkage rate- not reported<br>Treatment uptake- not reported<br>Cure assessment- 100% (193/193)<br>SVR- 99% (191/193)
Davidson[5]	2018	UK, Scotland	Primary care clinic offering OST	Treatment (DAA)	Prospective observational study of treatment program for HCV in homeless clinic	• Testing: not described<br>• Treatment: Nurse led<br>• Other features: Onsite support from PCP and consultant hepatologist. Concomitant treatment with opiates	n= 20	Testing uptake- not reported<br>NAT uptake- not reported<br>Linkage rate- not reported<br>Treatment uptake- not reported<br>Cure assessment- 100% (20/20)<br>SVR- 85% (17/20)

Morris*[6]	2018	Australia, Queensland	OST	Treatment (DAA)	Prospective observational study of HCV for PWID on OST	• Testing: not described<br>• Treatment: Nurse and PCP led treatment.<br>• Other features: Social worker facilitated referral, Concomitant OST, Mental health assessment	n= 476	Testing uptake- not reported<br>NAT uptake- not reported<br>Linkage rate- not reported<br>Treatment uptake- 72% (341/476)<br>Cure assessment- 62% (212/341)<br>SVR- 98% (202/212)
Mason[7]	2017	Canada, Toronto	Primary care clinic offering needle exchange	Treatment (DAA)	Prospective cohort study of HCV care at primary care clinic	• Testing: Not described<br>• Treatment: PCP and nurse led treatment.<br>• Other features: On site specialist support.	n= 74	Testing uptake- not reported<br>NAT uptake- not reported<br>Linkage rate- not reported<br>Treatment uptake- not reported<br>Cure assessment- 93% (69/74)<br>SVR- 90% (60/67)
Read[8]	2017	Australia, Sydney	Primary care clinic offering OST	Treatment (DAA)	Retrospective observational study of HCV treatment program in accessible community clinic	• Testing: not described<br>• Treatment: PCP led.<br>• Other features: Use of standard or enhanced (intensive) approach to treatment adherence supportive services	n= 72	Testing uptake- not reported<br>NAT uptake- not reported<br>Linkage rate- not reported<br>Treatment uptake- not reported<br>Cure assessment- 100% (59/59)<br>SVR- 100% (59/59)
Norton[9]	2017	USA, New York	Urban primary care clinic	Treatment (DAA)	Prospective cohort study for HCV treatment within adult primary care center	• Testing: not described<br>• Treatment: Hepatologist experienced with addiction care.	n= 121	Testing uptake- not reported<br>NAT uptake- not reported<br>Linkage rate- not reported<br>Treatment uptake- 74% (89/121)<br>Cure assessment- 100% (89/89)<br>SVR- 96% (85/89)
ETHOS study: Keats[10], Alavi[11]	2015	Australia, Newcastle/ New South Wales	OST	Treatment (IFN)	Prospective cohort study of HCV assessment and treatment in clinics for addiction care with limited experience in HCV	• Testing: Lab based EIA<br>• Treatment: by addiction specialist<br>• Other features: Focus on indigenous peoples, Peer worker counselling and referral support, adjacent NSP, client review by hepatologist if needed	n= 242	Testing uptake- not reported<br>NAT uptake- not reported<br>Linkage rate- not reported<br>Treatment uptake- 8% (20/242)<br>Cure assessment- 100% (20/20)<br>SVR- 75% (15/20)
Wade[12]	2015	Australia, Victoria	Primary care clinic offering OST	Linkage to care, Treatment (IFN/DAA)	Retrospective observational study of primary care-based treatment of HCV in PWID.	• Testing: not described<br>• Treatment: On-site Gastroenterologist/ID specialist<br>• Other features: referral for mental health services (as needed).	n= 279	Testing uptake- not reported<br>NAT uptake- not reported<br>Linkage rate- 67% (186/279)<br>Treatment uptake- 30% (55/186)<br>Cure assessment- 93% (51/55)<br>SVR- 65% (33/51)
Milne[13]	2015	Canada, British Columbia	Primary care clinic offering NE, OST	Treatment (IFN)	Descriptive study of Multidisciplinary based HCV treatment involving PCPs, NP, counsellors and psychiatrists.	• Testing: not described<br>• Treatment: PCP led<br>• Other features: Concomitant dispensary of Methadone (and ART, if applicable), Use of designated 'liver days' for care. Peer facilitated support groups. On site pharmacist to improve adherence.	n= 131	Testing uptake- not reported<br>NAT uptake- not reported<br>Linkage rate- not reported<br>Treatment uptake- not reported<br>Cure assessment- 100% (131/131)<br>SVR- 77% (101/131)

Brunner[14]	2013	Switzerland, Zurich	OST	Treatment (IFN)	Retrospective chart review of HCV treatment within a heroin maintenance program.	• Testing: not described<br>• Treatment: PCP and Internist led<br>• Other features: Consultation with off-site hepatologists, as needed. Nurse provided psychosocial support. counselling for substance abuse. Concomitant OST	n= 66	Testing uptake- not reported<br>NAT uptake- not reported<br>Linkage rate- not reported<br>Treatment uptake- not reported<br>Cure assessment- 100% (66/66)<br>SVR- 62% (41/66)
Seidenberg[15]	2013	Switzerland, Zurich	OST	Linkage to care, Treatment (IFN)	Retrospective chart review of HCV testing and treatment integrated into opioid maintenance therapy program.	• Testing: not described<br>• Treatment: PCP led testing and treatment<br>• Other features: Psychiatric assessment, Concomitant OST	n= 85	Testing uptake- not reported<br>NAT uptake- not reported<br>Linkage rate- 100% (85/85)<br>Treatment uptake- 41% (35/85)<br>Cure assessment- 100% (35/35)<br>SVR- 71% (25/35)
Newman[16]	2013	Canada, Toronto	Primary care clinic offering NE, OST	Treatment (IFN)	Prospective Observational study of HCV treatment in community clinic by multidisciplinary team	• Testing: not described<br>• Treatment: PCP led.<br>• Other features: Concomitant OST. Use of standardized treatment protocols, Psychiatric evaluation,	n= 34	Testing uptake- not reported<br>NAT uptake- not reported<br>Linkage rate- not reported<br>Treatment uptake- 41% (14/34)<br>Cure assessment- 100% (14/14)<br>SVR- 57% (8/14)
Bruce[17]	2012	USA, Connecticut	OST	Treatment (IFN)	RCT comparing modified DOT at OST clinic vs. self-administered therapy (SAT) at a liver specialty clinic	• Testing: not described<br>• Treatment: not described<br>• Other features: Referrals supported by social workers	n= 12 (Int. arm); n= 9 (Stan. arm)	Testing uptake- not reported<br>NAT uptake- not reported<br>Linkage rate- not reported<br>Treatment uptake- Int. arm- 83%; (10/12) Stan. Arm- 44% (4/9)<br>Cure assessment- not reported<br>SVR- Int. arm- 50% (6/12)<br>Stan. arm- 11% (1/9)
Stein[18]	2012	USA, New York	OST	Treatment (IFN/DAA)	Retrospective chart review of HCV testing and treatment in OST by onsite family physicians familiar with HIV care.	• Testing: not described.<br>• Treatment: PCP and Internist led<br>• Other features: DOT, psychological evaluation, peer led psychosocial support on and off-site for full treatment duration.	n= 42	Testing uptake- not reported<br>NAT uptake- not reported<br>Linkage rate- not reported<br>Treatment uptake- not reported<br>Cure assessment- 100% (42/42)<br>SVR- 41% (11/27)
Lindenburg (DUTCH-C project)[19]	2011	Netherlands, Amsterdam	OST/Primary care clinic	Testing, Treatment (IFN)	Prospective cohort study of multidisciplinary HCV treatment in PWID without advanced disease	• Testing: Lab based EIA and Reflex HCV VL test.<br>• Treatment: PCP and hepatologist	n= 497	Testing uptake- 90% (449/497)<br>NAT uptake- 100% (267/267)<br>Linkage rate- not reported<br>Treatment uptake- not reported<br>Cure assessment- 98% (57/58)<br>SVR- 65% (37/57)
Litwin[20]	2011	USA, New York	OST	Treatment (IFN)	Randomized controlled trial comparing effects of DOT on treatment adherence and virologic	• Testing: not described<br>• Treatment: PCP led<br>• Other features: DOT, use of peer support groups	n= 59	Testing uptake- not reported<br>NAT uptake- not reported<br>Linkage rate- not reported<br>Treatment uptake- 68% (40/59)<br>Cure assessment- not reported

					outcomes among PWID to non-DOT			SVR- not reported
Grebely[21]	2010	Canada, Vancouver	Primary care clinic offering needle exchange, OST	Treatment (IFN)	Retrospective chart review of on-site HCV treatment by multidisciplinary team-	• Testing: not described<br>• Treatment: ID specialist, follow up by NP and counsellors	n= 109	Testing uptake- not reported<br>NAT uptake- not reported<br>Linkage rate- not reported<br>Treatment uptake- 52% (57/109)<br>Cure assessment- 33% (19/57)<br>SVR- 63% (12/19)
Jack[22]	2009	UK, Nottingham	Primary care clinic offering OST	Testing, Linkage to care, Treatment (IFN)	Prospective observational study of HCV treatment in primary care practice proving OST	• Testing: Lab-based EIA and VL Treatment: PCP led treatment<br>• Other features: onsite specialists support if needed	n= 353	Testing uptake- 75% (266/353)<br>NAT uptake- 100% (174/174)<br>Linkage rate- 73% (86/118)<br>Treatment uptake- 35% (30/86)<br>Cure assessment- 100% (30/30)<br>SVR- 43% (13/30)
John-Baptiste[23]	2008	Canada, Toronto	OST	Treatment (IFN)	Retrospective chart review of HCV treatment in OST clinic for PWID without advanced liver disease.	• Testing: not described<br>• Treatment: Trained PCPs, ID specialist.<br>• Other features: Concomitant OST. psychological assessment, onsite specialists support if needed	n= 109	Testing uptake- not reported<br>NAT uptake- not reported<br>Linkage rate- not reported<br>Treatment uptake- not reported<br>Cure assessment- 100% (109/109)<br>SVR- 56% (61/109)
Alavi[24]	2018	Iran, Tehran	OST, DIC	Testing, Treatment (DAA)	NRS to evaluate on-site HCV testing, linkage to care and treatment uptake in OST (Stan. Arm) vs. DICs (Int. arm)	• Testing: RDT, Lab-based EIA<br>• Treatment: not described	n= 270 (Stan. arm) n= 166 (Int. arm)	Testing uptake- not reported<br>NAT uptake- 100% (Stan. Arm) (270/270), 100% (Int. Arm) (166/166)<br>Linkage rate- not reported<br>Treatment uptake- 100% (Stan. Arm) (40/40) 88% (Int. Arm) (46/52)<br>Cure assessment- not reported<br>SVR- not reported
Gayam[25]	2018	USA, New York	Primary care clinic offering needle exchange, OST	Treatment (DAA)	Retrospective cohort study of HCV treatment in a community care setting	• Testing: not described<br>• Treatment: not described<br>• Other features: Concomitant use of Opioid substitution therapy	n= 181	Testing uptake- not reported<br>NAT uptake- not reported<br>Linkage rate- not reported<br>Treatment uptake- not reported<br>Cure assessment- not reported<br>SVR- 95% (147/181)
Schubert[26]	2018	Austria, Vienna	OST	Treatment (DAA)	Prospective observational study of treatment embedded in OST site	• Testing: not described<br>• Treatment: Provided by pharmacist, GP, or nurse<br>• Other features: Concomitant OST	n= 249	Testing uptake- not reported<br>NAT uptake- not reported<br>Linkage rate- not reported<br>Treatment uptake- not reported<br>Cure assessment- 72% (179/249)<br>SVR- 99% (178/179)
Kaberg[27]	2018	Sweden, Stockholm	NSP	Treatment (DAA)	Prospective observational study of testing and treatment	• Testing: venepuncture, liver function tests, use of TE for liver staging<br>• Treatment: No reimbursement restrictions	n= 203	Testing uptake- not reported<br>NAT uptake- not reported<br>Linkage rate- not reported<br>Treatment uptake- 21% (42/203)<br>Cure assessment- 45% (19/42)<br>SVR- 89% (17/19)

Selfridge[28]	2018	Canada, Victoria	Primary care clinic offering needle exchange, OST	Treatment (DAA)	Retrospective observational study of testing and treatment provided by on-site multidisciplinary team	• Testing: not described<br>• Treatment: Nurse-led<br>• Other features: Onsite TE for liver staging, harm reduction and counselling provided	n= 273	Testing uptake- not reported<br>NAT uptake- not reported<br>Linkage rate- not reported<br>Treatment uptake- not reported<br>Cure assessment- 90% (246/273)<br>SVR- 98% (241/246)
Traeger[29]	2018	Australia, Victoria	Primary care clinics offering needle exchange, OST	Treatment (DAA)	Retrospective chart review of a nurse-led model of care in the community	• Testing: Nurse-led<br>• Treatment: Nurse-led	n= 241	Testing uptake- not reported<br>NAT uptake- not reported<br>Linkage rate- not reported<br>Treatment uptake- 38% (92/241)<br>Cure assessment- 24% (22/68)<br>SVR- 95% (21/22)
Edwards[30]	2018	UK, London	Primary care clinic offering needle exchange, OST	Testing, Treatment (DAA)	Prospective observational study of nurse led clinic for HCV testing and treatment	• Testing: Nurse-led, DBS<br>• Treatment: Nurse-led<br>• Other features: OST prescription dates amended to encourage HCV treatment adherence	n= 65	Testing uptake- 92% (60/65)<br>NAT uptake- not reported<br>Linkage rate- not reported<br>Treatment uptake- 75% (36/48)<br>Cure assessment- 84% (26/31)<br>SVR- 96% (25/26)
Biggart[31]	2018	UK, Glasgow	Addictions clinic (OST)	Treatment (DAA)	Retrospective observational study of treatment in a community outreach clinic	• Testing: Performed by liver nurse specialist<br>• Treatment: F0-2 can be treated by nurses whilst F3-4 require consultant review	n= 69	Testing uptake- not reported<br>NAT uptake- not reported<br>Linkage rate- not reported<br>Treatment uptake- 78% (54/69)<br>Cure assessment- 54% (29/54)<br>SVR- 97% (28/29)
Page[32]	2018	Australia, Sydney	NSP	Testing, Linkage to care, Treatment (DAA)	Retrospective observational study of nurse led testing and treatment at NSP.	• Testing: not described<br>• Treatment: Nurse-led	n= 319	Testing uptake- 45% (145/319)<br>NAT uptake- not reported<br>Linkage rate- 51% (32/71)<br>Treatment uptake- 69% (22/32)<br>Cure assessment- 95% (21/22)<br>SVR- not reported
Macbeth[33]	2018	UK, Edinburgh	Homeless clinic	Treatment (DAA)	Retrospective chart review of a hepatologist-led outreach service at a homeless clinic	• Testing: not described<br>• Treatment: Nurse-led<br>• Other features: HCV medication linked into OST to monitor adherence	n= 49	Testing uptake- not reported<br>NAT uptake- not reported<br>Linkage rate- not reported<br>Treatment uptake- not reported<br>Cure assessment- 57% (28/49)<br>SVR- 92% (25/28)
Middleton[34]	2018	UK, Glasgow	OST clinic, Hospital	Treatment (DAA)	Retrospective comparative study of HCV treatment integrated within OST (Int. arm) compared to traditional hospital-based services (Stan. Arm)	• Testing: not described<br>• Treatment: Nurse-led (Int. arm)<br>• Other features: Specialist care for patients with advanced liver disease	n= 47 (Int. arm)<br><br>n= 51 (Stan. arm)	Testing uptake- not reported<br>NAT uptake- not reported<br>Linkage rate- not reported<br>Treatment uptake- 98% (Int. arm) (46/47); 27% (Stan. Arm) (14/51)<br>Cure assessment- not reported<br>SVR- not reported
Rehak[35]	2018	Czech Republic, Prague	OST	Treatment (IFN)	Retrospective chart review on-site	• Testing: not described	n= 343	Testing uptake- not reported<br>NAT uptake- not reported<br>Linkage rate- not reported

					treatment and medical care	• Treatment: Integrated treatment in a substance use centre<br>• Other features: Integrated provision of counselling, psychotherapy and harm reduction services		Treatment uptake- not reported<br>Cure assessment- not reported<br>SVR- 82% (280/343)
Ryder[36]	2018	Australia, Sydney	OST, Sexual health clinic	Treatment (DAA)	Retrospective observational study of HCV treatment at sexual health clinic adjacent to OST service	• Testing: MDT<br>• Treatment: MDT<br>• Other features: Tailored treatment and follow-up to individual needs, with SMS reminders and social work support	n= 79	Testing uptake- not reported<br>NAT uptake- not reported<br>Linkage rate- not reported<br>Treatment uptake- not reported<br>Cure assessment- 71% (56/79)<br>SVR- 96% (54/56)
Bourke[37]	2018	Dublin, Ireland	OST	Treatment (DAA)	Retrospective observational pilot project of treatment at addiction services centers	• Testing: PCR test<br>• Treatment: GP led<br>• Other features: Fibrosis restrictions. Peer and group support provided	n= 79	Testing uptake- not reported<br>NAT uptake- not reported<br>Linkage rate- not reported<br>Treatment uptake- 67% (31/79)<br>Cure assessment- 100% (31/31)<br>SVR- 100% (31/31)
Bajis[38]	2018	Australia, Sydney	Homeless service clinic	Testing, Linkage to care, Treatment (DAA)	Prospective observational study of HCV care within hospital	• Testing: venous sample for HCV VL, HCV VL using Gene Xpert, use of TE for liver staging<br>• Treatment: GP led	n= 205	Testing uptake- not reported<br>NAT uptake- 99% (202/205)<br>Linkage rate- 62% (29/47)<br>Treatment uptake- 79% (23/29)<br>Cure assessment- 65% (15/23)<br>SVR- 100% (15/15)
Pedrana[39]	2018	Australia, Melbourne	NSP	Testing, Linkage to care, Treatment (DAA)	Retrospective observational study of NSP centered, same-day testing and treatment	• Testing: RDT, HCV VL using GeneXpert orGenedrive<br>• Treatment: GP led	n= 174	Testing uptake- not reported<br>NAT uptake- 93% (140/150)<br>Linkage rate- 63% (48/76)<br>Treatment uptake- 90% (43/48)<br>Cure assessment- not reported<br>SVR- not reported
Radley[40]	2018	UK, Dundee	OST	Linkage to care, Treatment (DAA)	RCT comparing HCV testing and treatment in pharmacy (Int. arm) vs. hospital care (Stan. arm).	• Testing: DBS, Lab-based EIA and HCV lab NAT<br>• Treatment: Pharmacist led (Int. arm)	n= 545 (Int. arm)<br><br>n= 540 (Stan. arm)	Testing uptake- not reported<br>NAT uptake- not reported<br>Linkage rate- 39% (Int. arm) (215/545) 26% (Stan. arm) (140/540)<br>Treatment uptake- 52% (Int. arm) (112/215) 44% (Stan. arm) (62/140)<br>Cure assessment- 96% (Int. arm) (108/112) 95% (Stan. arm) (59/62)<br>SVR- not reported
Gilliver[41]	2018	Australia, Sydney	OST	Testing, Treatment (DAA)	Retrospective observational study of testing and treatment at OST site	• Testing: Recall of patients with a previous positive HCV RNA PCR test, then retested<br>• Treatment: not described	n= 112	Testing uptake- not reported<br>NAT uptake- 95% (116/122)<br>Linkage rate- not reported<br>Treatment uptake- 76% (52/68)<br>Cure assessment- 77% (40/52)

								SVR- 100% (40/40)
Midgard[42]	2018	Norway, Oslo	NSP	Treatment (DAA)	Retrospective observational study of HCV treatment integrated within harm reduction services	• Testing: not described<br>• Treatment: GP and nurse led	N= 263	Testing uptake- not reported<br>NAT uptake- not reported<br>Linkage rate- not reported<br>Treatment uptake- 77% (202/263)<br>Cure assessment- not reported<br>SVR- not reported
Bieser[43]	2018	USA, Boston	Homeless service clinic	Treatment (DAA)	Retrospective chart review of treatment outcomes.	• Testing: not described<br>• Treatment: not described<br>• Other features: Concomitant administration of OST, frequent patient counselling	n= 515	Testing uptake- not reported<br>NAT uptake- not reported<br>Linkage rate- not reported<br>Treatment uptake- 58% (300/515)<br>Cure assessment- 90% (271/300)<br>SVR- 94% (254/271)
Young[44]	2018	Australia, Inala	OST	Treatment (DAA)	Retrospective observational study of nurse and GP care within hospital	• Testing: not described<br>• Treatment: GP led<br>• Other features: Involvement of peer workers for support	n= 150	Testing uptake- not reported<br>NAT uptake- not reported<br>Linkage rate- not reported<br>Treatment uptake- 89% (133/150)<br>Cure assessment- not reported<br>SVR- 89% (119/133)
O'Loan[45]	2018	Australia, Brisbane	Mobile units	Linkage to care, Treatment (DAA)	Retrospective chart review of testing and treatment outcomes	• Testing: Not described, use of TE for liver staging<br>• Treatment: Nurse and GP led	n= 116	Testing uptake- not reported<br>NAT uptake- not reported<br>Linkage rate- 76% (88/116)<br>Treatment uptake- 89% (78/88)<br>Cure assessment- 47% (37/78)<br>SVR- 96% (23/24)
Von Bibra[46]	2018	Australia, Melbourne	NSP, mobile units	Treatment (DAA)	Retrospective chart review of nurse led testing and treatment from a mobile unit	• Testing: Not described, use of TE for liver staging<br>• Treatment: Nurse and GP led	n= 264	Testing uptake- not reported<br>NAT uptake- not reported<br>Linkage rate- not reported<br>Treatment uptake- 66% (174/264)<br>Cure assessment- 69% (120/174)<br>SVR- not reported
Olaizola[47]	2018	France, Bordeaux	Mobile unit	Linkage to care, Treatment (DAA)	Retrospective observational study of testing in addiction clinics and treatment in an outreach centers	• Testing: RDT<br>• Treatment: Nurse-led<br>• Other features: nurse facilitated referrals.<br>•	n= 19	Testing uptake- not reported<br>NAT uptake- not reported<br>Linkage rate- 79% (15/19)<br>Treatment uptake- 71% (10/14)<br>Cure assessment- 80% (8/10)<br>SVR- 88% (7/8)
Taylor[48]	2018	USA, multiple states	Community health centers or OST	Treatment (DAA)	RCT comparing effect of facilitated referral and modified DOT on treatment uptake	• Testing: not described<br>• Treatment: Sofosbuvir/velpatasvir for 12 weeks<br>• Other features: Those at community centres record themselves taking medication	n= 651	Testing uptake- not reported<br>NAT uptake- not reported<br>Linkage rate- not reported<br>Treatment uptake- 92% (601/651)<br>Cure assessment- not reported<br>SVR- not reported
McClure et al[49]	2017	Australia, Melbourne	(i) Tertiary clinic (ii and iii)	Treatment (DAA)	Prospective comparative study of	• Testing: not described	n=548 (i:	Testing uptake- not reported<br>NAT uptake- not reported

			Community, primary care clinics		treatment assessment supervised by specialist liver clinic for (i) specialist-led HCV treatment (tertiary liver clinic), (ii) nurse-led HCV treatment (in community), and (iii) GP led treatment (in community) facilitated by telehealth	• Treatment: Treatment with offsite specialist support available if needed; PCP remote consultation; specialist led care<br>• Other features: Telehealth	specia list)<br>n=67 (ii: nurse led)<br>n=46 (iii: GP led)	Linkage rate- not reported<br>Treatment uptake- not reported<br>Cure assessment- not reported<br>SVR- 93% (508/548) (i: Specialist); 88% (59/67) (ii: Nurse); -<br>94% (43/46) (iii: GP)
**Embedded clinic/Visiting Specialist**								
Lukhwaro (MdM)[50]	2017	Kenya, Nairobi	NSP	Testing, Treatment (DAA)	Prospective observational study of HCV testing and treatment at DIC.	• Testing: RDT, immediate sample collection for HCV VL test<br>• Treatment: Led by Infectious disease specialists<br>• Other features: DOT, 1-month counselling prior to treatment initiation, defaulters traced via peer-educators	n= 105	Testing uptake- not reported<br>NAT uptake- 75% (79/105)<br>Linkage rate- not reported<br>Treatment uptake- not reported<br>Cure assessment- 100% (44/44)<br>SVR- 98% (43/44)
Barnett[51]	2018	Canada, Victoria	Nurse visiting community centres	Treatment (DAA)	Retrospective chart review of testing and treatment at supportive housing facilities	• Testing: RDTs, use of TE for liver staging<br>• Treatment: Nurse led<br>• Other features: STI screening and treatments incorporated into visits	n= 47	Testing uptake- not reported<br>NAT uptake- not reported<br>Linkage rate- not reported<br>Treatment uptake- 87% (41/47)<br>Cure assessment- not reported<br>SVR- not reported
Fragomeli[52]	2015	Australia, New South Wales	OST	Treatment (IFN)	Descriptive study of liver clinic for HCV care integrated into OST center on specific weekdays.	• Testing: not described<br>• Treatment: Hepatologist led<br>• Other features: Concomitant OST and HCV treatment to improve compliance. Routine psychological assessment.	n= 300	Testing uptake- not reported<br>NAT uptake- not reported<br>Linkage rate- not reported<br>Treatment uptake- 13% (40/300)<br>Cure assessment- not reported<br>SVR- not reported
Sockaling-ham[53]	2013	Canada, Toronto	NSP	Treatment (IFN)	Retrospective chart review of HCV treatment for PWID and persons with severe mental illnesses (SMI).	• Testing: not described<br>• Treatment: ID specialist led<br>• Other features: concurrent weekly psychoeducational support group	n= 24	Testing uptake- not reported<br>NAT uptake- not reported<br>Linkage rate- not reported<br>Treatment uptake- not reported<br>Cure assessment- 100% (24/24)<br>SVR- 71% (17/24)
Ho[54]	2013	USA, San Jose, California	Homeless clinic	Treatment (IFN)	Prospective observational study of HCV treatment in pts with mental illnesses/ substance use in homeless clinic.	• Testing: not described<br>• Treatment: PCP led.<br>• Other features: adequate attendance (>75%) before treatment initiation, weekly assessment by PCP, psychologist and psychiatric evaluation.	n= 76	Testing uptake- not reported<br>NAT uptake- not reported<br>Linkage rate- not reported<br>Treatment uptake- 39% (30/76)<br>Cure assessment- 100% (30/30)<br>SVR- 63% (17/24)

Wilkinson[55]	2008	UK, London	OST	Linkage to care, Treatment (IFN)	Retrospective observational study on HCV treatment facilitated by monthly liver clinic outreach to addiction unit	• Testing: lab-based EIA.<br>• Treatment: Hepatologist led.<br>• Other features: Use of records to identify previously diagnosed persons, psychiatrist review	n= 411	Testing uptake- not reported<br>NAT uptake- not reported<br>Linkage rate- 20% (83/411)<br>Treatment uptake- 76% (63/83)<br>Cure assessment- 57% (36/63)<br>SVR - 58% (21/36)
Ulstein[56]	2018	Norway, Oslo	OST	Treatment (DAA)	Retrospective observational study of testing and treatment provided by on-site multidisciplinary team	• Testing: not described<br>• Treatment: GP and nurse-led, ID specialist available if needed	n= 110	Testing uptake- not reported<br>NAT uptake- not reported<br>Linkage rate- not reported<br>Treatment uptake- not reported<br>Cure assessment- 85% (94/110)<br>SVR- 99% (93/94)
Sander-Hess[57]	2018	UK, Stoke	OST	Testing, Linkage to care, Treatment (DAA)	Retrospective observational study hepatologist led treatment in an addiction clinic	• Testing: DBS for EIA and HCV RNA PCR<br>• Treatment: Hepatologist-led	n= 395	Testing uptake- 75% (302/395)<br>NAT uptake- 100% (63/63)<br>Linkage rate- 100% (31/31)<br>Treatment uptake- 100% (31/31)<br>Cure assessment- 55% (17/31)<br>SVR- 88% (15/17)
Testing and Referral for Treatment (Partial decentralization)								
Kikvidze[58]	2018	Georgia, Tbilisi	NSP- Medical center	Testing, Linkage to care, treatment (DAA)	Prospective observational study of HCV testing and counselling at NSP and treatment service at medical center	• Testing: RDT, lab-based HCV VL test<br>• Treatment: Led by Infectious disease specialists, (> F2 patients)<br>• Other features: Peer based counselling, Follow up at HRC for counselling and VL test at 6- and 12-months post SVR	n= 2600	Testing uptake- 21% (554/2600)<br>NAT uptake- not reported<br>Linkage rate- 97% (338/350)<br>Treatment uptake- 74% (244/331)<br>Cure assessment- 98% (234/239)<br>SVR- 88% (207/234)
Magaldi (C a Difference program)[59]	2018	USA, Philadelphia	OST	Testing, Linkage to Care	Descriptive study of CBO-led HCV testing in OST clinics, and referral to subspecialty centers for treatment	• Testing: RDTs, immediate on-site sample collection for HCV VL test.<br>• Treatment: not described<br>• Other features: Referrals supported by patient navigators	n= 403	Testing uptake- not reported<br>NAT uptake- 89% (358/403)<br>Linkage rate- 39% (77/200)<br>Treatment uptake- not reported<br>Cure assessment- not reported<br>SVR- not reported
Sutton[60]	2017	USA, Georgia	OST	Testing, Linkage to Care	Retrospective cohort study on HCV testing service co-facilitated by CBO and State health dept HIV testing program.	• Testing: Lab based EIA and reflex HCV VL test<br>• Treatment: not described<br>• Other features: referrals supported by patient navigator<br>•	n= 973	Testing uptake- not reported<br>NAT uptake- 92% (895/973)<br>Linkage rate- 52% (369/710)<br>Treatment uptake- not reported<br>Cure assessment- not reported<br>SVR- not reported
Porter[61]	2017	USA, Seattle	NSP	Testing, Linkage to Care	Prospective observational study of co-located HCV testing and NSP services	• Testing: Lab based EIA and Reflex HCV VL test<br>• Treatment: not described	n= 125	Testing uptake- not reported<br>NAT uptake- 73% (91/125)<br>Linkage rate- 16% (7/44)<br>Treatment uptake- not reported<br>Cure assessment- not reported<br>SVR- not reported

Blackburn (HepTLC)[62]	2016	USA, Multiple cities	NSP, Primary care clinics	Testing, Linkage to care	Prospective observational of HCV testing service in local health depts, STI clinics, community care organizations, NSPs.	• Testing: Birth cohort screening, lab-based EIA and reflex HCV VL test<br>• Treatment: not described<br>• Other features: referrals supported by peer workers	n= 3495	Testing uptake- not reported<br>NAT uptake- 47% (1630/3495)<br>Linkage rate- 23% (198/861)<br>Treatment uptake- not reported<br>Cure assessment- not reported<br>SVR- not reported
Rajkumar[63]	2016	India, Manipur	Communities	Testing	Descriptive study of outreach screening and referrals for PWID, FSW, MSM, truckers, and migrant workers.	• Testing: RDT via lay workers,<br>• Treatment: not described<br>• Other features: Enrolment: During CBO-led advocacy/ awareness campaigns. Community worker supported referral	n= 1659	Testing uptake- not reported<br>NAT uptake- 54% (889/1659)<br>Linkage rate- not reported<br>Treatment uptake- not reported<br>Cure assessment- not reported<br>SVR- not reported
Wong (New Life Liver project)[64]	2014	Hong Kong SAR	Rehabilitation center- Tertiary Hospital	Testing, Linkage to care, Treatment (IFN)	Prospective cohort study of HCV testing at Rehab center and referral for treatment at tertiary hospital	• Testing: POC RDTs. Lab based HC VL test<br>• Treatment: Specialist led<br>• Other features: Peer based counselling, facilitated referral by volunteer MDs, ex-PWIDs and social workers. Routine follow up for HCC monitoring	n= 111	Testing uptake- not reported<br>NAT uptake- 88% (98/111)<br>Linkage rate- 70% (69/98)<br>Treatment uptake- 38% (26/69)<br>Cure assessment- 35% (9/26)<br>SVR- 89% (8/9)
Masson[65]	2013	USA, New York/ San Francisco	OST	Linkage to care	RCT comparing linkage to care in patients with on-site screening and motivational case management vs. routine education and referral	• Testing: not described<br>• Treatment: not described<br>• Other features: Referrals supported by social workers	n= 149 (Int. arm); n= 137 (cont. arm)	Testing uptake- not reported<br>NAT uptake- not reported<br>Linkage rate: int. arm 65% (97/149); control arm 37%, (OR:4.1) (51/137)<br>Treatment uptake- not reported<br>Cure assessment- not reported<br>SVR- not reported
Islam[66]	2012	Australia, New South Wales	Primary care clinic offering NE- Liver clinic	Testing, Linkage to care	Retrospective chart review of nurse led HCV triage and referral to liver clinic for treatment.	• Testing: not described<br>• Treatment: not described<br>• Other features: Referrals supported by nurses	n= 479	Testing uptake- 74% (353/479)<br>NAT uptake- 93% (197/212)<br>Linkage rate- 71% (68/96)<br>Treatment uptake- not reported<br>Cure assessment- not reported<br>SVR- not reported
Martinez[67]	2012	USA, New York	OST- Liver clinic	Testing, Linkage to care, Treatment (IFN)	Retrospective observational study of HCV treatment by addiction medicine specialist embedded in hepatitis clinic	• Testing: not described<br>• Treatment- Internist led.<br>• Other features: Designated clinic day for HCV, supervision by hepatologist as needed.	n= 257	Testing uptake- not reported<br>NAT uptake- 86% (222/257)<br>Linkage rate- 61% (76/125)<br>Treatment uptake- 32% (24/76)<br>Cure assessment- 100% (24/24)<br>SVR- 68% (13/19)
Surey[68]	2018	UK, London	Mobile unit	Treatment (DAA)	Retrospective observational study of testing at a mobile unit	• Testing: DBS<br>• Treatment: not described<br>• Other features: peer facilitated referrals	n= 104	Testing uptake- not reported<br>NAT uptake- not reported<br>Linkage rate- not reported<br>Treatment uptake- 86% (89/104)

					for homeless persons and referral for care			Cure assessment- not reported<br>SVR- not reported
Anagnostou[69]	2018	Greece, Athens	OST	Treatment (DAA)	Retrospective observational study of assessment at OST clinic with treatment at outpatient liver unit	• Testing: not described<br>• Treatment: Hepatologist-led<br>• Other features: Reimbursement criteria based on liver stiffness and HIV status	n= 104	Testing uptake- not reported<br>NAT uptake- not reported<br>Linkage rate- not reported<br>Treatment uptake- 68% (71/104)<br>Cure assessment- 89% (63/71)<br>SVR- not reported
Foroghi[70]	2018	Italy, Rome	Harm reduction services	Linkage to care, Treatment (DAA)	Retrospective observational study of on-site testing at harm reduction services and referral to an ID clinic for treatment	• Testing: RDT<br>• Treatment: ID specialist<br>• Other features: Counselling	n= 829	Testing uptake- not reported<br>NAT uptake- not reported<br>Linkage rate- 51% (49/97)<br>Treatment uptake- 65% (32/49)<br>Cure assessment- not reported<br>SVR- not reported
Dominguez[71]	2018	France, Paris	OST and IDU care settings	Testing, Treatment (DAA)	Retrospective observational study of on-site testing at harm reduction services with treatment prescribed in hospital	• Testing: Not described, TE for liver staging<br>• Treatment: not described	n= 488	Testing uptake- not reported<br>NAT uptake- 79% (387/488)<br>Linkage rate- not reported<br>Treatment uptake- 78% (259/322)<br>Cure assessment- not reported<br>SVR- 95% (246/259)
Losikoff[72]	2018	USA, New Bedford	OST	Treatment (DAA)	Retrospective chart review of testing onsite with referral to community providers for evaluation and treatment	• Testing: Lab based EIA and Reflex HCV VL test<br>• Treatment: Community provider	n= 69	Testing uptake- not reported<br>NAT uptake- not reported<br>Linkage rate- not reported<br>Treatment uptake- not reported<br>Cure assessment- 77% (53/69)<br>SVR- 98% (52/53)
Bielen[73]	2018	Belgium, Limburg	OST	Testing, Linkage to care, Treatment (DAA)	Retrospective chart review of on-site screening and case management, with treatment at hospital	• Testing: DBS analysed at lab<br>• Treatment: Nurse led<br>• Other features: Concomitant OST	n= 482	Testing uptake- not reported<br>NAT uptake- 81% (392/482)<br>Linkage rate- 85% (85/114)<br>Treatment uptake- 39% (33/85)<br>Cure assessment- 97% (32/33)<br>SVR- 91% (16/17)
Fuchs++[74]	2018	Canada, Saskatchewa n	Community clinics	Testing, Treatment (DAA)	Retrospective observational study on testing integrated into routine harm reduction services and referral for treatment	• Testing: Reflex HCV VL tests, TE for liver staging<br>• Treatment: not described<br>• Other features: Many persons of self-declared indigenous heritage. Fibrosis restrictions.	n= 487	Testing uptake- not reported<br>NAT uptake- 93% (454/487)<br>Linkage rate- not reported<br>Treatment uptake- 75% (153/203)<br>Cure assessment- not reported<br>SVR- 82% (126/153)
Harney[75]	2018	Australia, Melbourne	Homeless services	Testing, Treatment (DAA)	Retrospective observational study of testing and assessment by a nurse at homeless services, with treatment provided in primary care	• Testing: Nurse-led<br>• Treatment: Prescribed in primary care	n= 67	Testing uptake- not reported<br>NAT uptake- 78% (52/67)<br>Linkage rate- not reported<br>Treatment uptake- 62% (24/39)<br>Cure assessment- not reported<br>SVR- not reported

Antonini[76]	2018	France, Villejuif	Drug addiction services (OST)	Testing, Linkage to care	Retrospective chart review with testing at site with linkage to a hepatologist for treatment within 2 weeks	• Testing: DBS, point of care GeneXpert for HCV viral load<br>• Treatment: not described<br>• Other features: Social services also provided	n= 26	Testing uptake- 100% (26/26)<br>NAT uptake- 47% (9/19)<br>Linkage rate- 50% (2/4)<br>Treatment uptake- not reported<br>Cure assessment- not reported<br>SVR- not reported
Holeska[77]	2018	Canada, Vancouver	Community pop up clinics at community centres	Linkage to care, Treatment (DAA)	Retrospective observational study of community testing and peer supported linkage to care	• Testing: RDTs<br>• Treatment: not described<br>• Other features: Facilitated referrals by peer workers	n= 126	Testing uptake- not reported<br>NAT uptake- not reported<br>Linkage rate- 30% (34/126)<br>Treatment uptake- 44% (12/27)<br>Cure assessment- not reported<br>SVR- not reported
Swan[78]	2018	Ireland, Dublin; UK, London; Romania, Bucharest	Primary care clinics	Linkage to care, Treatment (DAA)	Prospective observational study of testing at primary/community care site and treatment at a hepatology/ID service	• Testing: not described<br>• Treatment: Specialist led	n= 141	Testing uptake- not reported<br>NAT uptake- not reported<br>Linkage rate- 73% (103/141)<br>Treatment uptake- 34% (35/103)<br>Cure assessment- 71% (25/35)<br>SVR- 72% (18/25)
No Decentralization								
Alimohammed[79]	2018	Canada, Vancouver	Infectious disease center	Treatment (DAA)	Retrospective chart review of treatment at an ID clinic	• Testing: not described<br>• Treatment: Led by multi-disciplinary team	n= 225	Testing uptake- not reported<br>NAT uptake- not reported<br>Linkage rate- not reported<br>Treatment uptake- not reported<br>Cure assessment- not reported<br>SVR- 90% (203/225)
De Gijsel[80]	2018	USA, New England	Tertiary care center	Testing, Linkage to care, Treatment (DAA)	Retrospective chart review of tertiary care-based testing and treatment	• Testing: Lab-based EIA and HCV VL test<br>• Treatment: Specialist led	n= 65	Testing uptake- not reported<br>NAT uptake- 100% (65/65)<br>Linkage rate- 26% (9/34)<br>Treatment uptake- 56% (5/9)<br>Cure assessment- 100% (5/5)<br>SVR- 100% (5/5)
Ucbilek[81]	2018	Turkey, Mersin	University Gastroenterolog y and Infectious disease clinic	Treatment (DAA)	Retrospective chart review of study with treatment at specialist clinic	• Testing: not described<br>• Treatment: not described	n= 35	Testing uptake- not described<br>NAT uptake- not described<br>Linkage rate- not described<br>Treatment uptake- not described<br>Cure assessment- 100% (35/35)<br>SVR- 97% (34/35)

(Ab) Antibody, (APRI) AST to Platelet Ratio Index, (ART) Antiretroviral therapy, (BC) Birth Cohort, (CBO) Community Based Organization, (DAA) Direct Acting Antiviral, (N/A) Data Not Available, (DBS) Dried Blood Spot, (DIC) Drop-in Centre; (DOT) Direct Observed Therapy, (EIA) Enzyme Immuno-Assay, (FD) Full decentralization, (GT) Genotype, (HBV) Hepatitis B Virus, (ID) Infectious Disease, (IDU) Injecting drug use, (IFN) Interferon, (LFT) Liver Function Tests, (LTFU) Loss to Follow up, (MAT) Medically Assisted Therapy, (MMTP) Methadone Maintenance therapy program, (NAT) Nucleic acid test, (ND) No decentralization, (NP) Nurse Practitioner, (NSP) Needle Syringe Program, (OMT) Opioid maintenance therapy, (OST) Opioid substitution therapy, (PCP) Primary care physician, (PCR) Polymerase chain reaction, (PD) Partial Decentralization, (PLHIV) Persons Living with HIV, (PN) Patient Navigator, (PWID) Persons Who Inject Drugs, (RCT) Randomised control trial, (RDT) Rapid Diagnostic Test, (RNA) Ribonucleic acid, (Rx) Treatment, (SIF) Safe Injection Facility, (SMI) Severe Mental Illness, (STI) Sexually Transmitted Illness, (SVR) Sustained Virologic Response, (TE) Transient Elastography, (USS) Ultrasound, (VL) Viral load
*++ singular study split across populations * Outcomes updated with 2018 data*

B. People in Prisons

Full Decentralization and Integration								
Study	Year	Country/ Region	Setting(s)	Scope of care extracted	Study Design	Key Interventions	N	Reported Outcomes
Morey[82]	2018	UK, Durham	Prisons	Testing, Treatment (DAA)	Prospective observational study of HCV testing and treatment service in prisons facilitated by telehealth	• Testing: Opt-out testing, Lab based EIA (DBS) and HCV VL test<br>• Treatment: Nurse led<br>• Other features: specialist physician support available through Telehealth.	n= 2821	Testing uptake- 53% (1495/2821)<br>NAT uptake- 100% (95/95)<br>Linkage rate- not reported<br>Treatment uptake- 71% (57/80)<br>Cure assessment- not reported<br>SVR- not reported
Lloyd[83]	2013	Australia, Sydney	1 Max security and 2 rural prisons	Treatment (IFN)	Prospective cohort study of Nurse led HCV triage and treatment for inactive injectors in prisons	• Testing: not described<br>• Treatment: Nurse led<br>• Other features: Hepatologist review via telehealth, use of pre-developed protocols.	n= 141	Testing uptake- not reported<br>NAT uptake- not reported<br>Linkage rate- not reported<br>Treatment uptake- 77% (108/141)<br>Cure assessment- 63% (68/108)<br>SVR- 69% (47/68)
Fridriksdottir[84]	2018	Iceland	All Icelandic prisons	Testing, Treatment (DAA)	Prospective cohort study of nurse-led recruitment, testing, and treatment in prisons	• Testing: Nurse led<br>• Treatment: Nurse led<br>• Other features: Efforts made to link to care outside prison upon release	n= 68	Testing uptake- not reported<br>NAT uptake- 87% (59/68)<br>Linkage rate- not reported<br>Treatment uptake- 94% (16/17)<br>Cure assessment- not reported<br>SVR- not reported
Bachelard[85]	2018	France, Ile de France	Prisons	Treatment (DAA)	Prospective cohort study of testing and treatment with the prison setting	• Testing: not described<br>• Treatment: supervised by a coordination and mediation team	n= 69	Testing uptake- not reported<br>NAT uptake- not reported<br>Linkage rate- not reported<br>Treatment uptake- 68% (47/69)<br>Cure assessment- not reported<br>SVR- not reported
Levy[86]	2018	Australia, Canberra	Prisons	Treatment (DAAs)	Retrospective study of nurse led testing and treatment in prisons	• Testing: not described<br>• Treatment: Nurse led	n= 80	Testing uptake- not reported<br>NAT uptake- not reported<br>Linkage rate- not reported<br>Treatment uptake- not reported<br>Cure assessment- not reported<br>SVR- 98% (78/80)
Farley[87]	2018	Canada, Vancouver	Prisons	Treatment (DAAs)	Retrospective chart review of treatment in prisons by trained nurses and GPs with specialist input if needed	• Testing: not described<br>• Treatment: GP led	n= 439	Testing uptake- not reported<br>NAT uptake- not reported<br>Linkage rate- not reported<br>Treatment uptake- not reported<br>Cure assessment- 89% (389/439)<br>SVR- 98% (381/389)

Alliance for Public Health[88]	2017	Ukraine, Kyiv	Prisons	Treatment (DAA)	Descriptive study of HCV testing and treatment within Ukraine penitentiary system	• Testing: Lab based EIA and HCV VL test<br>• Treatment: not described<br>• Other features: Collaboration with the ministry of Justice.	n= 50	Testing uptake- not reported<br>NAT uptake- not reported<br>Linkage rate- not reported<br>Treatment uptake- not reported<br>Cure assessment- not reported<br>SVR- 98% (49/50)
Papaluca et al[89]	2019	Australia, Victoria	14 adult prisons, with OST programs (Exclusions: those with sentences less than duration of treatment	Treatment (DAA)	Prospective observational study of nurse-led treatment supported by telehealth in prisons	• Testing: Cirrhosis assessment/liver stagingwith TE<br>• Treatment: Nurse led treatment with offsite specialist support via telehealth<br>• If released early, prisoners provided with remaining treatment for continuation<br>• SVR assessments not necessarily in prison if prisoners already released	n=313	Testing uptake- not reported<br>NAT uptake- not reported<br>Linkage rate- not reported<br>Treatment uptake- not reported<br>Cure assessment- not reported<br>SVR- 96% (303/313)
Cuadrado et al[90]	2018	Spain, Cantabria	Prison	Testing, linkage to care, Treatment (DAA)	Prospective observational study of HCV testing and treatment in prisons supported by telehealth	• Testing: Opt-out<br>• Treatment: Specialist led. follow up facilitated by use of Telehealth between specialists and prison medical team (nurses, pharmacists)<br>• Treatment offered to those with a sentence > 30 days	n= 851	Testing uptake- 13% (110/851)<br>NAT uptake- 78% (86/110)<br>Linkage rate- 100% (69/69)<br>Treatment uptake- 100% (69/69)<br>Cure assessment- 93% (64/69)<br>SVR- 95% (61/64)
Full Decentralization and Integration (outside closed settings)								
Hawks[91]	2016	USA, New York	Transitions (Post-incarceration) clinic	Testing and Linkage to care and Treatment (IFN)	Retrospective cohort study of HCV testing and treatment service for newly released prisoners in post-incarceration clinic	• Testing: not described<br>• Treatment: PCP trained in HCV management<br>• Other features: Enrolment facilitated by formerly incarcerated community health worker, collaboration with CBO and criminal justice system	n= 451	Testing uptake- 70% (317/451)<br>NAT uptake- 88% (93/106)<br>Linkage rate- 100% (84/84)<br>Treatment uptake- 10% (8/84)<br>Cure assessment- 100% (8/8)<br>SVR- 38% (3/8)
Embedded clinic/Visiting Specialist								
Mohamed[92]	2018	UK, London	Prison	Linkage to care, Treatment (DAA)	Retrospective study of testing and treatment within prison hepatology clinic	• Testing: RDT, GeneXpert for HCV VL<br>• Treatment: Hepatologist led<br>• Other features: Treatment commenced if length of incarceration> length of treatment to avoid LTFU	n= 62	Testing uptake- not reported<br>NAT uptake- not reported<br>Linkage rate- 77% (48/62)<br>Treatment uptake- 26% (11/43)<br>Cure assessment- not reported<br>SVR- not reported

Boonwaat[93]	2010	Australia, New South Wales	Prisons	Treatment (IFN)	Retrospective observational study of specialist led HCV management through once monthly prison visit.	• Testing: not described<br>• Treatment: Gastroenterologist<br>• Other features: Coverage of indigenous populations. Program developed by Justice Health	n= 371	Testing uptake- not reported<br>NAT uptake- not reported<br>Linkage rate- not reported<br>Treatment uptake- 50% (185/371)<br>Cure assessment- 62% (115/185)<br>SVR- 44% (51/115)	
Testing and Referral for Treatment (Partial decentralization)									
Jack (C-INSIDE study)[94]	2018	UK, East Midlands	Prisons	Testing	Retrospective chart review of HCV testing service within prisons and referral for treatment post release	• Testing: Opt-out, Lab based EIA (DBS).<br>• Treatment: not described	n= 20075	Testing uptake- 8% (1643/20075)<br>NAT uptake- not reported<br>Linkage rate- not reported<br>Treatment uptake- not reported<br>Cure assessment- not reported<br>SVR- not reported	
Winter[95]	2016	Australia, Victoria	Prisons	Testing	Retrospective comparative study of weekly nurse led testing in STI clinic at prisons versus baseline	• Testing: Sample collection for lab-based EIA by nurse<br>• Treatment: not described	n= 285 (Baseline); n= 280 (Intervention arm)	Testing uptake- 62% (174/280)<br>NAT uptake- not reported<br>Linkage rate- not reported<br>Treatment uptake- not reported<br>Cure assessment- not reported<br>SVR- not reported	
Schoen Bachler[96]	2016	USA, Carolinas	Medium to small sized jails.	Testing, Linkage to care	Prospective observational study of HCV testing within prisons and linkage to care post-release	• Testing: Confidential opt-out HCV screening, Targeted – Birth cohort, persons with tattoos. Sample collection for EIA, Lab based reflex HCV VL test<br>• Other features: PN facilitated referral (prior to release	n= 106	Testing uptake - not reported<br>NAT uptake- 89% (94/106)<br>Linkage rate- 50% (12/25)<br>Treatment uptake- not reported<br>Cure assessment- not reported<br>SVR- not reported	
Sena[97]	2016	USA, Carolinas	County jail	Testing	Prospective observational study of HCV testing service within prisons and linkage to care post-release	• Testing: Sample collection for EIA and lab-based Reflex HCV VL test<br>• Treatment: not described<br>• Other features: PN facilitated referral	n= 89	Testing uptake- not reported<br>NAT uptake- 90% (80/89)<br>Linkage rate- not reported<br>Treatment uptake- not reported<br>Cure assessment- not reported<br>SVR- not reported	
Beckwith[98]	2015	USA, Rhode Island	Minimum security prisons	Testing, Linkage to care	Prospective observational study of HCV testing within prisons and linkage to care post-release	• Testing: RDT, immediate phlebotomy for HCV VL test coordinated by prison medical staff.<br>• Treatment: not described<br>• Other features: referral supported by correctional facility staff	n= 957	Testing uptake- 26% (252/957)<br>NAT uptake- 92% (23/25)<br>Linkage rate- 33% (4/12)<br>Treatment uptake- not reported<br>Cure assessment- not reported<br>SVR- not reported	
Cocoros (SHAPE initiative)[99]	2014	USA, Massa-chusetts	State Prison	Testing, Linkage to care	Prospective observational of HCV testing within prisons, and linkage to care at	• Testing: Opt-in. Samples for EIA collected at point of prison entry/orientation. Routine HCV VL test post release.	n= 2176	Testing uptake- 22% (596/2716)<br>NAT uptake- not reported<br>Linkage rate- 38% (31/82)<br>Treatment uptake- not reported	

					primary care clinics post release	• Treatment: not described<br>• Other features: Education of inmates on HCV on entry, integration of HCV services into existing HIV program, PN facilitated referral prior to release		Cure assessment- not reported<br>SVR- not reported
Perrett[100]	2011	UK, Cardiff	Substance use unit within prison	Testing, Linkage to care	Descriptive study of HCV testing within substance misuse unit at a local prison	• Testing: lab-based EIA, reflex HCV VL test<br>• Treatment: not described<br>• Other features: referral facilitated by NP	n= 24	Testing uptake- not reported<br>NAT uptake- 100% (24/24)<br>Linkage rate- 42% (8/19)<br>Treatment uptake- not reported<br>Cure assessment- not reported<br>SVR- not reported
Fuchs++[74]	2018	Canada, Saskatchewan	Prisons	Testing, Linkage to care, treatment (DAA)	Retrospective observational study of patients referred and treated at ID clinics from prisons	• Testing: not described<br>• Treatment: ID specialist<br>• Other features: Treatment reimbursement restrictions	n= 71	Testing uptake- not reported<br>NAT uptake- 99% (70/71)<br>Linkage rate- 81% (46/57)<br>Treatment uptake- 39% (7/18)<br>Cure assessment- not reported<br>SVR- 100% (7/7)

*(EIA/ELISA) Enzyme Immune Assay, (IFN) Interferon based regimen, (LTFU) Loss to Follow up, (PN) Patient Navigator, (PLHIV) Persons Living with HIV, (RDT) Rapid Diagnostic Test (STI) Sexually Transmitted Infection, (SRA) Stringent Regulatory Authority, (SVR) Sustained Virologic Response, (VL) Viral Load * Test for cure as of when due (n=7)*

C. People Living with HIV (PLHIV)

Full Decentralization								
Study	Year	Country/ Region	Setting(s)	Scope of care extracted	Study Design	Key Interventions	N	Reported Outcomes
Doyle (co-EC study)[101]	2018	Australia, Melbourne	Primary care clinic	Treatment (DAA)	Non-randomized comparative study of HCV treatment outcomes in primary clinics and tertiary centers.	• Testing: not described<br>• Treatment: Nurse led (at either location), General physician support available.	• n= 105 (Prim. Care); n= 48 (Ter. Care)	Testing uptake- not reported<br>NAT uptake- not reported<br>Linkage rate- not reported<br>Treatment uptake- not reported<br>Cure assessment- not reported<br>SVR- Prim, care- 95% (99/105); Ter. clinic- 100% (48/48)
Cachay[102]	2013	USA, California	HIV/ STI clinic	Treatment (IFN)	Retrospective comparative cohort study of management of HIV-HCV coinfection in primary care clinic for HIV care or hepatology (Hep.) clinics	• Testing: not described<br>• Treatment: ID specialists (Primary care clinic) and Hepatologists (hepatology clinic)	• n= 193 (Prim. Care);<br>• n= 163 (Hep. Model)	Testing uptake- not reported<br>NAT uptake- not reported<br>Linkage rate- not reported<br>Treatment uptake- Stan. Arm (Hep model)- 16% (26/163); Prim. care model- 25% (48/193)<br>Cure assessment- Stan. Arm (Hep model)- 100% (48/48); Prim. care model- 100% (26/26)<br>SVR- Stan. Arm (Hep model)- 35% (9/26); Prim. care model- 44% (21/48)
Traeger[103]	2018	Australia, Melbourne	Primary care	Testing, Treatment (DAA)	Retrospective chart review of testing and treatment for PLHIV in primary care clinics	• Testing: not described<br>• Treatment: Primary care clinics	• n= 94	Testing uptake- not reported<br>NAT uptake- 64% (119/185)<br>Linkage rate- not reported<br>Treatment uptake- 64% (60/94)<br>Cure assessment- 95% (54/57)<br>SVR- 98% (53/54)
MSF-Mozam-bique[104]	2018	Mozambique, Maputo	HIV/ STI clinic	Testing, Treatment (DAA)	Observational study of HCV testing and treatment integrated with routine HIV care at tertiary center	• Testing: RDT, DBS, near GeneXpert POC HCV VL test (onsite)<br>• Treatment: Primary care physician led	• n= 1448	Testing uptake- 97% (1398/1448)<br>NAT uptake- 100% (49/49)<br>Linkage rate- not reported<br>Treatment uptake- 60% (25/42)<br>Cure assessment- 80% (20/25)<br>SVR- 100% (20/20)
MSF-Myanmar[104]	2018	Myanmar, Dawei	HIV/ STI clinic	Testing, Treatment (DAA)	Observational study of HCV testing and treatment integrated with routine HIV care at tertiary center	• Testing: RDT, DBSnear GeneXpert POC HCV VL test (onsite)<br>• Treatment: Nurse led	• n= 4625	Testing uptake- 100% (4625/4625)<br>NAT uptake- 90% (356/394)<br>Linkage rate- not reported<br>Treatment uptake- 63% (225/356)<br>Cure assessment- 100% (225/225)<br>SVR- 98% (221/225)

(ART) Antiretroviral Therapy, (DAA) Direct Acting Antiviral, (FD) Full decentralization, (GT) Genotype, (LFT) Liver Function Tests, (ID) Infectious Diseases, (NP) Nurse Practitioner, (PLHIV) Persons Living with HIV, Primary Care Physician (PCP), (RNA) Ribonucleic acid, (Rx) Treatment, (STI) Sexually Transmitted Illness, (SVR) Sustained Virologic Response, (TE) Transient Elastography, (VL) Viral Load

D. General Population

Study	Year	Country/ Region	Setting(s)	Scope of care described	Study Design	Key Interventions	N	Outcomes
**Full Decentralization (Testing and Treatment)**								
Ford (Check Hep-C Program)[105]	2018	USA, New York	Outpatient clinics	Testing, Linkage to care, Treatment (DAA)	Prospective cohort study of HCV testing and treatment	• Testing: Targeted (based on risk profile), RDT, immediate blood sample for HCV VL test<br>• Treatment- Trained PCPs<br>• Other features: 5-day initial training sessions. Use of telehealth for provider support and training weekly, PN supported engagement, result notification and referrals	n= 881	Testing uptake- not reported<br>NAT uptake- 77% (678/881)<br>Linkage rate- 85% (435/512)<br>Treatment uptake- 30% (14/47)<br>Cure assessment- 43% (6/14)<br>SVR- 100% (6/6)
Belperio[106]	2017	USA, Multiple states	Urban, rural outpatient clinics	Testing, Linkage to care, Treatment (DAA)	Retrospective observational study of HCV testing and treatment in clinics by non-specialist providers.	• Testing: Birth Cohort automated clinician reminders, Lab-based reflex HCV VL test.<br>• Treatment: Led by NP, PA and pharmacists.<br>• Other features: Registry based recall of persons with positive tests, Use of telehealth/electronic databases for e-consults and clinical case discussions	n= 180337	Testing uptake- not reported<br>NAT uptake- not reported<br>Linkage rate- 93% (168410/180337)<br>Treatment uptake- 59% (100028/168410)<br>Cure assessment- not reported<br>SVR – 84% (84192/100028)
Capileno (MSF)[107]	2017	Pakistan, Karachi	Primary care clinic	Treatment (DAA)	Retrospective cohort study of HCV treatment service in primary care clinic	• Testing: RDTs, Lab-based HCV VL testing<br>• Treatment: Specialist led	n= 1012	Testing uptake- not reported<br>NAT uptake- not reported<br>Linkage rate- not reported<br>Treatment uptake- 17% (169/1012)<br>Cure assessment- 90% (152/169)<br>SVR- 93% (141/152)
Mera[108]	2016	USA, Cherokee Nation, Oklahoma	Outpatient clinics	Testing, Treatment (DAA)	Retrospective chart review of HCV testing and treatment in Indian health service	• Testing: Birth cohort, EMR based reminder for clinical decision support<br>• Treatment: PCP led following telehealth-based trainings.	n= 92012	Testing uptake- 18% (16772/92012)<br>NAT uptake- 68% (488/715)<br>Linkage rate- not reported<br>Treatment uptake- 57% (223/388)<br>Cure assessment- 90% (201/223)<br>SVR- 90% (180/201)
**Embedded clinic/Visiting Specialist**								

Jayasekera[109]	2015	USA, California	Rural outreach clinic	Treatment (DAA)	Retrospective observational study of HCV treatment facilitated by bi-monthly visit by specialist	• Testing: not described<br>• Treatment: Hepatologist led<br>• Other features: off-site support available for real time monitoring through EHR system	n= 58	Testing uptake- not reported<br>NAT uptake- not reported<br>Linkage rate- not reported<br>Treatment uptake- not reported<br>Cure assessment- 97% (56/58)<br>SVR- 91% (51/56)
Full Decentralization (Treatment only)								
Kattakuzhy (ASCEND study)[110]	2017	USA, D.C.	Urban Outpatient clinics	Treatment (DAA)	NRS comparing treatment of HCV cases with similar baseline clinical characteristics across 3 provider types- NP, PCP or specialist.	• Testing: not described<br>• Treatment: by various health providers following a uniform 3-hr training course.	n= 600	Testing uptake- not reported<br>NAT uptake- not reported<br>Linkage rate- not reported<br>Treatment uptake- not reported<br>Cure assessment- NP- 94% (141/150), PCP- 92% (147/160), Specialist- 91% (263/290)<br>SVR- NP- 95% (n=134/141)<br>PCP- 95% (n=139/147), Specialist- 92% (n=243/263).
Dhiman[111]	2018	India, Punjab	District hospitals	Treatment (DAA)	Prospective observational study of HCV treatment service in district hospitals.	• Testing: not describe<br>• Treatment: PCP led treatment,<br>• Other features: Use of telehealth for patient review, monthly patient follow up to assess for adverse event compliance.	n= 35877	Testing uptake- not reported<br>NAT uptake- not reported<br>Linkage rate- not reported<br>Treatment uptake- not reported<br>Cure assessment- 76% (27113/35877)<br>SVR- 91% (27113/24770)
Cooper[112]	2017	Canada, Ontario	Primary care clinics	Treatment (DAA)	Retrospective comparative study of outcomes in HCV patients in remote locations treated using telehealth (TH) vs. non-TH	• Testing: not described<br>• Treatment: Nurse led<br>• Other features: Specialist support available through telehealth	n= 43 (TM), n= 608 (non-TM)	Testing uptake- not reported<br>NAT uptake- not reported<br>Linkage rate- not reported<br>Treatment uptake-<br>non-TH- 53% (319/608), TH- 63% (27/43)<br>Cure assessment- non-TH- 78% (249/319), TH- 70% (19/27)<br>SVR-<br>Non-TH- 95% (236/249), TH- 95% (18/19)
Lasser[113]	2015	USA, Massachu-setts	District hospital	Linkage to care, Treatment (DAA)	Retrospective observational study of HCV treatment in patients without advanced liver disease	• Testing: not described<br>• Treatment: Trained internist.<br>• Other: Linkage to care facilitated by social workers. Fibrosis restrictions.	n= 302	Testing uptake- not reported<br>NAT uptake- not reported<br>Linkage rate- 51% (157/302)<br>Treatment uptake- not reported<br>Cure assessment- 67% (46/69)<br>SVR- 100% (46/46)
Baker[114]	2014	Australia, New South Wales	Urban and rural primary care clinics	Treatment (IFN)	Pilot study: Non-specialist assessment and treatment for HCV patients (GT 2 and 3) without advanced liver disease.	• Testing: not described<br>• Treatment: PCP led<br>• Other features: psychiatric assessment,	n= 34	Testing uptake- not reported<br>NAT uptake - not reported<br>Linkage rate- not reported<br>Treatment rate- not reported<br>Cure assessment- 91% (31/34)

								SVR- 84% (26/31)
Nazareth[115]	2013	Australia, Western Australia	Rural hospital	Treatment (IFN)	Comparative Prospective cohort of NP led treatment using telehealth with hepatologist support versus face-to-face (FTF) care at tertiary/specialist sites.	• Testing: not described<br>• Treatment: Nurse led	n= 528 (FTF); n= 53 (Tele-health)	Testing uptake- not reported<br>NAT uptake- not reported<br>Linkage rate- not reported<br>Treatment uptake- Telehealth 94% (50/53)<br>Cure assessment- Telehealth 86% (43/50)<br>SVR- FTF- 59% (311/528)<br>Telehealth- 84% (36/43)
Arora[116]	2011	USA, New Mexico	Primary care clinics	Treatment (IFN)	Prospective comparative study of HCV treatment in patients without advanced liver disease at rural areas (Intervention arm) or University HCV clinic (Standard Arm) – also in prisons	• Testing: not described<br>• Treatment: PCP led.<br>• Other features: APRI based assessment, Routine case discussions with multidisciplinary specialist team via telehealth	n= 261 (Int. arm); n= 146 (Stan. arm)	Testing uptake- not reported<br>NAT uptake- not reported<br>Linkage rate- not reported<br>Treatment uptake- not reported<br>Cure assessment- Int. arm- 100% (261/261), Stan. arm- 100% (146/146)<br>SVR- Int. arm- 58% (152/261)<br>Stan. arm- 58% (84/146)
Myers (APPROACH study)[117]	2011	Canada, Multi-city	Tertiary center and Community clinics	Treatment (IFN)	Prospective comparative study of HCV treatment in patients with similar baseline characteristic at community clinics (Int. arm) or Tertiary centers (Stan. Arm)	• Testing: not described<br>• Treatment: Specialist led.	n= 251 (Int. arm); n= 134 (Stan. arm)	Testing uptake- not reported<br>NAT uptake- not reported<br>Linkage rate- not reported<br>Treatment uptake- Int. arm- 100% (251/251), Stan. Arm- 99% (133/134)<br>Cure assessment- not reported<br>SVR- Int. arm- 48% (120/251)<br>Stan. arm- 59% (79/133)
Rossaro[118]	2008	USA Yuba City, California	Primary care clinics	Treatment (IFN)	Retrospective chart review of telehealth-based HCV treatment service for residents in rural areas.	• Testing: not described<br>• Treatment: PCP led,<br>• Other features: Focus on patients without advanced liver disease, Specialist support using TM. Fibrosis restrictions apply	n= 103	Testing uptake- not reported<br>NAT uptake- not reported<br>Linkage rate- not reported<br>Treatment uptake- 14% (14/103)<br>Cure assessment- 100% (14/14)<br>SVR- 36% (5/14)
Hill[119]	2008	Canada, British Columbia	Rural and urban clinics	Treatment (IFN)	Prospective observational study of HCV treatment in locally accessible clinics	• Summary: Physician led assessment and treatment of HCV cases in locally accessible clinics<br>• Testing: not described<br>• Treatment: PCP led	n= 471	Testing uptake- not reported<br>NAT uptake- not reported<br>Linkage rate- not reported<br>Treatment uptake- 77% (363/471)<br>Cure assessment- 56% (205/363)<br>SVR- 61% (126/205)

Jack[120]	2013	UK, Nottingham	At-home	Treatment (IFN)	Retrospective observational study of at-home HCV treatment following initial hospital assessments.	• Testing: not described<br>• Treatment: Specialist led. Treatment administration implemented by trained nurses.<br>• Other features: Down referral following initial assessments.	n= 81	Testing uptake- not reported<br>NAT uptake- not reported<br>Linkage rate- not reported<br>Treatment uptake- not reported<br>Cure assessment- 100% (81/81)<br>SVR- 44% (36/81)
Testing and Referral for Treatment (Partial decentralization)								
Shiha*[121]	2018	Egypt, Al-Othmanya	Communities/specialist clinic	Testing, Linkage to care, Treatment (DAA)	Prospective observational program for community testing and referral to liver center for treatment.	• Testing: RDTs, sample collection for HCV VL test immediately afterwards.<br>• Referrals:<br>• Assessment, staging: At liver center- TE, HBV, LFT<br>• Treatment: Hepatologist<br>• Other features: Use of 'village promoters' for community engagement, advocacy and counselling, Transportation available for group referrals.	n= 184693	Testing uptake- 92% (170618/184693)<br>NAT uptake- 100% (29553/29553)<br>Linkage rate- 100% (14414/14414)<br>Treatment uptake- 90% (13017/15139)<br>Cure assessment- 100% (13006/13017)<br>SVR- 98% (12693/13006)
Anartati[122]	2018	Indonesia, Jakarta	Primary care clinics (Testing) / District hospitals (Treatment)	Testing, Treatment (DAA)	Retrospective chart review of HCV testing in primary care clinics and referral for treatment in district hospitals.	• Testing: Targeted (based on risk profile), RDTs, Lab based VL test<br>• Treatment: Trained PCPs<br>• Other features: staging with APRI score, Hepatologists support available if needed	n= 809	Testing uptake- not reported<br>NAT uptake- 84% (679/801)<br>Linkage rate- not reported<br>Treatment uptake- 81% (480/595)<br>Cure assessment- not reported<br>SVR- not reported
Teply[123]	2018	USA, Nebraska/ Iowa	Primary care clinics	Testing	Retrospective comparative study of Birth cohort (BC) HCV screening rates and linkage to care following introduction of EMR prompts.	• Summary: Retrospective comparative study of Birth cohort HCV screening rates and linkage to care following introduction of EMR prompts.<br>• Testing: Targeted (BC), use of EMR prompts<br>• Treatment: not described<br>• Other features: staging with APRI score	n= 35823 (Pre-alert); n= 37424 (post alert)	Testing uptake- Pre-alert- 2% (625/35823)<br>Post alert- 24% (8928/37424)<br>NAT uptake- Pre-alert- 100% (31/31)<br>Post-alert- 100% (155/155)<br>Linkage rate- not reported<br>Treatment uptake- not reported<br>Cure assessment- not reported<br>SVR- not reported
Ivantes[124]	2017	Brazil, Curitiba	Tertiary center	Treatment (DAA)	Retrospective cohort study of HCV treatment at reference center following referrals from basic health units	• Testing: not described<br>• Treatment: Specialist led	n= 456	Testing uptake- not reported<br>NAT uptake- not reported<br>Linkage rate- not reported<br>Treatment uptake- not reported<br>Cure assessment- 100% (456/456)<br>SVR- 93% (424/456)
Smyth[125]	2017	Canada, Prince	Primary care clinics (Testing) / District	Linkage to care,	Prospective observational study of HCV treatment service for	• Testing: Lab based EIA, HCV VL test<br>• Treatment: Hepatologist led	n= 242	Testing uptake- not reported<br>NAT uptake- not reported<br>Linkage rate- 51% (123/242)

		Edward Island	hospitals (Treatment)	Treatment (DAA)	persons in remote underserved areas	• Other features: referrals facilitated by NP		Treatment uptake- 76% (93/123)<br>Cure assessment- 90% (84/93)<br>SVR- 98% (82/84)
Coyle (HepTLC)[126]	2016	USA, Philadelphia	Outpatient clinics	Treatment (IFN), Testing, Linkage to care	Retrospective observational study of HCV screening and referrals for underserved and homeless populations.	• Testing: Opt-out, Lab based EIA and reflex HCV VL test, targeted screening- (BC, previous tattoo or IDU, blood transfusion).<br>• Treatment: not described<br>• Other features: referrals supported by patient navigators. Automated results delivery into patient healthcare file.	n= 488	Testing uptake- not reported<br>NAT uptake- 92% (451/488)<br>Linkage rate- 67% (220/330)<br>Treatment uptake- 11% (24/220)<br>Cure assessment- not reported<br>SVR- 63% (15/24)
Falade-Nwulia[127]	2016	USA, Baltimore	STI clinics	Treatment (DAA), Testing, Linkage to care	Retrospective observational study of community HCV testing and linkage to care	• Testing: RDT, immediate sample collection for reflex VL testing.<br>• Treatment: not described Other features: referrals supported by PN.	n= 4399	Testing uptake- 61% (2681/4399)<br>NAT uptake- 98% (185/189)<br>Linkage rate- 89% (138/155)<br>Treatment uptake- 46% (37/81)<br>Cure assessment- not reported<br>SVR- not reported
Trooskin[128]	2015	USA, Philadelphia	Mobile medical unit	Testing, Linkage to care, Treatment (IFN/DAA)	Retrospective observational study HCV screening and linkage to care service in underserved areas	• Testing: RDT, immediate blood draw for HCV VL test<br>• Treatment: not described<br>• Other features: referrals supported by PN, use of e-mail notifications and specialist appointment scheduling.	n= 48	Testing uptake- not reported<br>NAT uptake- 88% (42/48)<br>Linkage rate- 58% (21/36)<br>Treatment uptake- 57% (12/21)<br>Cure assessment- not reported<br>SVR- not reported
Litwin[129]	2012	USA, New York	Urban Primary care clinics	Testing	Retrospective comparative study of HCV screening uptake	• Testing: Birth cohort testing, Risk based screened facilitated by EMR prompts.<br>• Treatment: not described	n= 6591 (Baseline); n= 8981 (risk-screener)	Testing uptake- Baseline- 6% (394/6591)<br>Risk screener- 13% (1179/8981)<br>NAT uptake- not reported<br>Linkage rate- not reported<br>Treatment uptake- not reported<br>Cure assessment- not reported<br>SVR- not reported
No Decentralization								
Luma[130]	2018	Cameroon, Yaoundé	Specialist clinic	Testing, Linkage to care, Treatment (IFN)	Retrospective chart review of HCV testing and treatment in tertiary hospital	• Testing: RDT. Re-screenings performed with EIA to minimize false positive results. Sample transportation for HCV VL test/GT testing overseas.<br>• Treatment: Hepatologist led	n= 669	Testing uptake- not reported<br>NAT uptake- 61% (410/669)<br>Linkage rate- 52% (192/366)<br>Treatment uptake- 42% (81/192)<br>Cure assessment- 89% (72/81)<br>SVR- 61% (44/72)

Carnaúba Junior[131]	2017	Brazil, São Paolo	Specialist clinic	Treatment (DAA)	Pilot study of HCV treatment service in specialist out-patient units	• Testing: not described<br>• Treatment: ID physician/Hepatologist.	n= 455	Testing uptake- not reported<br>NAT uptake- not reported<br>Linkage rate- not reported<br>Treatment uptake- not reported<br>Cure assessment- 80% (398/455)<br>SVR- 98% (394/398)
Qureshi[132]	2017	Pakistan, Karachi	Specialist clinic/ Health research center	Treatment (DAA)	Prospective observational study of HCV treatment service in liver clinic.	• Testing: Lab based EIA, near Point of care HCV VL test<br>• Treatment: Led by Gastroenterologist<br>• Other features: On-treatment VL monitoring, APRI based staging, Coverage of treatment experienced and cirrhotic patients.	n= 447	Testing uptake- not reported<br>NAT uptake- not reported<br>Linkage rate- not reported<br>Treatment uptake- not reported<br>Cure assessment- 28% (126/447)<br>SVR- 80% (101/126)
Iwamoto (MSF)[133]	2017	Cambodia Phnom Penh	Tertiary center	Treatment (DAA)	Retrospective cohort study of HCV testing and treatment using simplified model approach to care in tertiary facility.	• Testing: RDT, HCV VL test<br>• Treatment: Led by Gastroenterologist	n= 1800	Testing uptake- not reported<br>NAT uptake- not reported<br>Linkage rate- not reported<br>Treatment uptake- not reported<br>Cure assessment- 46% (820/1800)<br>SVR- 95% (779/820)
Levin[134]	2016	USA, Wisconsin	Specialist clinic	Treatment (DAA)	Retrospective chart review of Multidisciplinary HCV treatment in an integrated care tertiary facility.	• Testing: not described<br>• Treatment: ID specialist<br>• Other features: Pharmacist and nurse led review of patient adherence	n= 133	Testing uptake- not reported<br>NAT uptake- not reported<br>Linkage rate- not reported<br>Treated uptake not reported<br>Cure assessment- 100% (133/133)<br>SVR– 93% (124/133)
Gallach[135]	2016	Spain, Sabadell	Specialist clinic	Treatment (IFN)	Prospective cohort study of HCV treatment by multidisciplinary (MDT) team versus routine (non-MDT) care.	• Testing: not described<br>• Treatment: Hepatologist led	n= 286 (Baseline); n= 228 (MDT)	Testing uptake- not reported<br>NAT uptake- not reported<br>Linkage rate- not reported<br>Treatment uptake- not reported<br>Cure assessment- Baseline- 100% (286/286), MDT- 100% (228/228)<br>SVR- Baseline- 47% (134/286), MDT- 50% (114/228)
Ahmed[136]	2013	UK, Bristol	Specialist clinic	Linkage to care, Treatment (IFN)	Retrospective comparative study of HCV treatment following routine outpatient care or multi-disciplinary care	• Testing: not described<br>• Treatment: Hepatologist led or by MDT	n= 56 (Routine); n= 26 (MDT)	Testing uptake- not reported<br>NAT uptake- not reported<br>Linkage rate- Routine- 89% (50/56), MDT- 96% (25/26)<br>Treatment uptake- Routine- 92% (46/50), MDT- 96% (24/25)<br>Cure assessment- Routine- 43% (20/46), MDT- 83% (20/24)<br>SVR- Routine- 55% (11/20), MDT- 90% (18/20)

Goel[137]	2017	USA, New York	Tertiary center	Testing, Linkage to care, Treatment (DAA)	Prospective cohort study of HCV testing and linkage to care	• Testing: Birth cohort testing supported by EMR alerts/ reminders. Routine RNA<br>• Treatment: Hepatologist led<br>• Other features: Patient navigator supported referrals	n= 9101	Testing uptake- 49% (4419/9101)<br>NAT uptake- 91% (134/147)<br>Linkage rate- 71% (60/84)<br>Treatment uptake- 53% (32/60)<br>Cure assessment- not reported<br>SVR- not reported
Malhotra[138]	2016	India, Haryana	Tertiary center	Treatment (IFN)	Descriptive study of Program for HCV community-based testing and treatment in tertiary centers.	• Testing: not described.<br>• Treatment: not described<br>• Other features: Enrolment/ referrals facilitated by community outreaches.	n= 1530	Testing uptake- not reported<br>NAT uptake- not reported<br>Linkage rate- not reported<br>Treatment uptake- not reported<br>Cure assessment- 93% (1423/1530)<br>SVR- 90% (1281/1423)
Turner[139]	2015	USA, Texas	Tertiary center	Testing, Linkage to care, Treatment (IFN/DAA)	Prospective cohort study of hospital-based HCV testing and linkage to care service for in-patients.	• Testing: Opt-out, EMR facilitated birth cohort testing in admitted patients, Lab based reflex HCV VL test.<br>• Treatment: not described<br>• Other features: community health worker supported linkage to care.	n= 3777	Testing uptake- 84% (3168/3777)<br>NAT uptake- 89% (214/240)<br>Linkage rate- 81% (108/134)<br>Treatment uptake- 5% (5/108)<br>Cure assessment- not reported<br>SVR- not reported
Woodrell[140]	2015	USA, New York	Specialist clinic	Treatment (IFN/DAA)	Retrospective observational study of Multidisciplinary HCV treatment for persons without advanced liver disease.	• Testing: not described<br>• Treatment: Internist led.<br>• Other features: Involvement of mental health and social work services for development of a unified care plan.	n= 92	Testing uptake- not reported<br>NAT uptake- not reported<br>Linkage rate- not reported<br>Treatment uptake- 43% (40/92)<br>Cure assessment- 65% (26/40)<br>SVR- 69% (18/26)
Kuo[141]	2015	Taiwan, Tainan	Specialist Clinic	Treatment (IFN)	Prospective observational study of HCV treatment following community testing	• Testing: not described<br>• Treatment: Specialist led (Hepatologist)	n= 197	Testing uptake- not reported<br>NAT uptake- not reported<br>Linkage rate- not reported<br>Treatment uptake- 42% (83/197)<br>Cure assessment- 77% (64/83)<br>SVR- 73% (47/64)

(Ab) Antibody, (AE) Adverse event, (BC) Birth Cohort, (CHB) Chronic Hepatitis B, (CHW) Community Health Worker, (DBS) Dried Blood spot, (EMR) Electronic Medical Record, (FSW) Female Sex Worker, (HCC) Hepatocellular Carcinoma, (HDV) Hepatitis D Virus, (HCW) Healthcare worker, (MSM) Men who have sex with men, (NP) Nurse Practitioner, (PA) Physician Assistant, (PCP) Primary Care Physician, (RTK) Rapid Test Kit, (Rx) Treatment, (SRA) Stringent regulatory authority, (SVR) Sustained Virologic response, (TE) Transient elastography, (USS) Ultrasound, (VL) Viral Load

Supplementary Table 2: Assessment of bias summary of the 142 included studies

	Risk of bias			
	Critical	Serious	Moderate	Low
PWID	8 (10%)	34 (43%)	24 (30%)	14 (18%)
General population	4 (11%)	6 (16%)	10 (27%)	17 (46%)
People in prisons	2 (10%)	9 (45%)	2 (10%)	7 (35%)
PLHIV	2 (40%)	0 (0%)	2 (40%)	1 (20%)
**Overall**	**16 (11%)**	**49 (35%)**	**38 (27%)**	**39 (27%)**

Supplementary Table 3: Outcomes across the cascade of care among people who inject drugs (PWID), persons in prisons, people living with HIV (PLHIV), and the general population.

Outcomes across cascade of care	Pooled estimate-PWID (n; N)	Pooled estimate-Prisoners (n; N)	Pooled estimate-PLHIV (n; N)	Pooled estimate-General Population (n; N)
Serologic testing uptake	78% (95%CI 52–96) (n=9; 5219 persons)	35% (95%CI 17–55) (n=7; 28151 persons)	99% (95%CI 93–100) (n=2; 6073 persons)	36% (95%CI 8–71) (n=7; 382801 persons)
NAT uptake	90% (95%CI 82–96) (n=24; 10809 persons; p-value=0.741)	93% (95%CI 87–97) (n=9; 694 persons)	89% (95%CI 66–100) (n=3; 628 persons)	91% (95%CI 77–99) (n=12; 33925 persons; p-value=0.100)
Linkage to care	61% (95%CI 51–71) (n=32; 6299 persons; p-value=0.559)	74% (95%CI 51–91) (n=9; 505 persons)	N/A	79% (95%CI 69–87) (n=13; 196999 persons; p-value=0.051)
Treatment uptake	DAA: 69% (95%CI 62–75) (n=36; 5052 persons; p-value=0.521)<br><br>IFN: 41% (95%CI 28–55) (n=13; 1350 persons; p-value=0.143)	DAA: 68% (95%CI 45–87) (n=6; 296 persons)<br><br>IFN: 44% (95%CI 14–77) (n=3; 596 persons)	DAA: 63% (95%CI 59–67) (n=3; 492 persons)<br><br>IFN: 20% (95%CI 12–30) (n=2; 356 persons)	DAA: 57% (95%CI 44–70) (n=11; 186506 persons; p-value=0.484)<br><br>IFN: 66% (95%CI 42–86) (n=14; 1970 persons; p-value=0.913)
Uptake of cure assessment	DAA: 84% (95%CI 78–90) (n=38; 3960 persons; p-value=0.715)<br><br>IFN: 96% (95%CI 87–100) (n=17; 878 persons; p-value=0.069)	DAA: 89% (95%CI 87–92) (n=2; 508 persons)<br><br>IFN: 69% (95%CI 55–82) (n=3; 301 persons)	DAA: 95% (95%CI 79–100) (n=3; 307 persons)<br><br>IFN: 100% (95%CI 98–100) (n=2; 74 persons)	DAA: 85% (95%CI 72–94) (n=18; 53757 persons; p-value=0.850)<br><br>IFN: 91% (95%CI 82–97) (n=15; 3267 persons; p-value=0.234)
Sustained virologic response	DAA: 95% (95%CI 93–96) (n=40; 3947 persons; p-value=0.649)<br><br>IFN: 65% (95%CI 57–71) (n=20; 1098 persons; p-value=0.435)	DAA: 98% (95%CI 97–99) (n=6; 903 persons)<br><br>IFN: 53% (95%CI 32–73) (n=3; 191 persons)	DAA: 98% (95%CI 96–100) (n=5; 452 persons)<br><br>IFN: 40% (95%CI 29–52) (n=2; 74 persons)	DAA: 94% (95%CI 91–97) (n=19; 143403 persons; p-value=0.064)<br><br>IFN: 63% (95%CI 53–73) (n=19; 3856 persons; p-value=0.012)

n- number of study arms included
N- sample size
95%CI- 95% confidence interval
DAA- Direct acting antivirals
IFN- interferon
NAT- Nucleic acid test
p-value- Results of Begg's test for publication bias; only conducted when there are ≥10 studies

Supplementary figures 1 A – E: Impact of decentralization and integration on (A) NAT Uptake, (B) IFN treatment uptake, (C) IFN cure assessment, (D) DAA cure assessment, and (E) SVR 12 for IFN, for PWID, the general population, people in prisons, and PLHIV.

A.

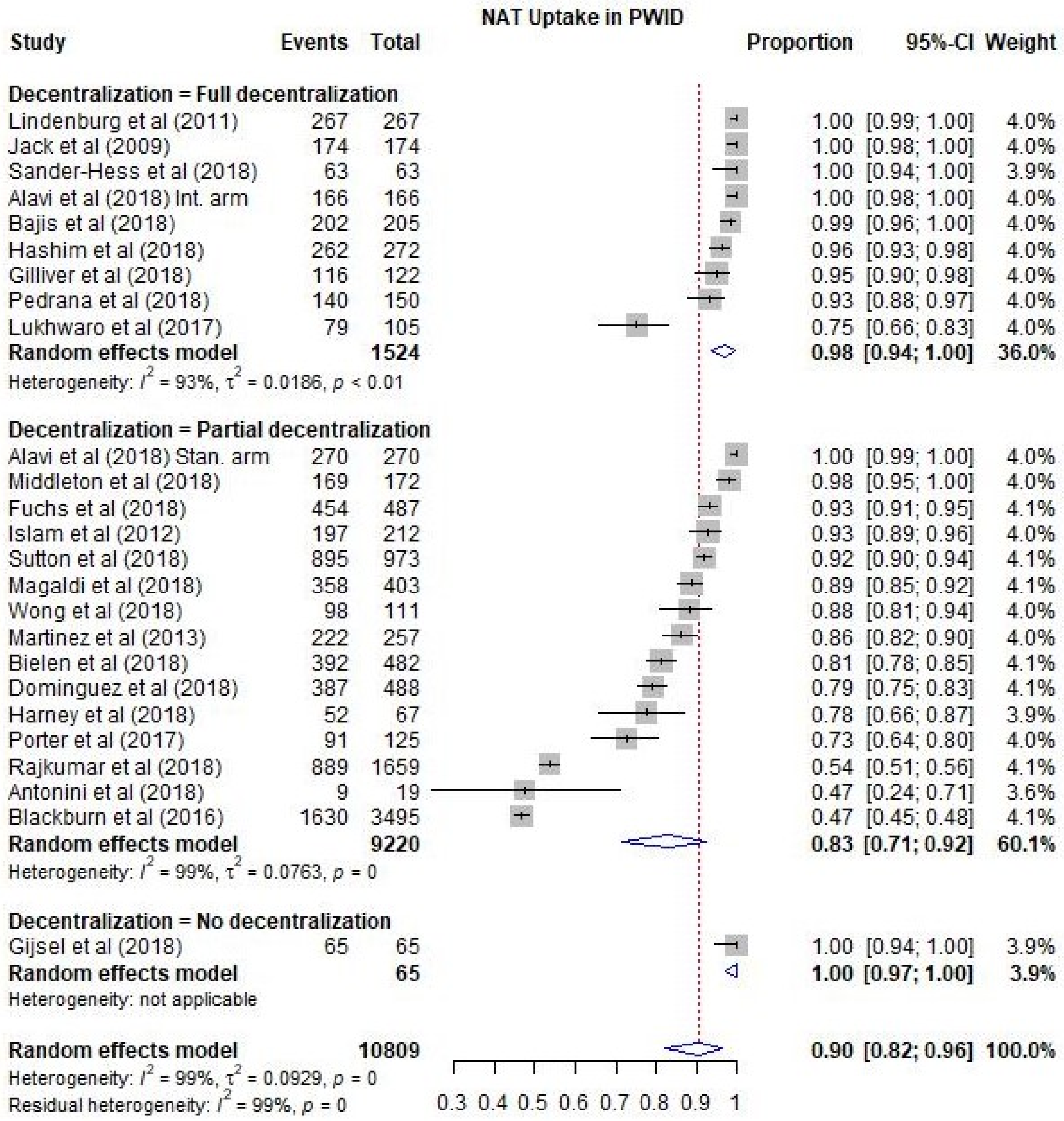

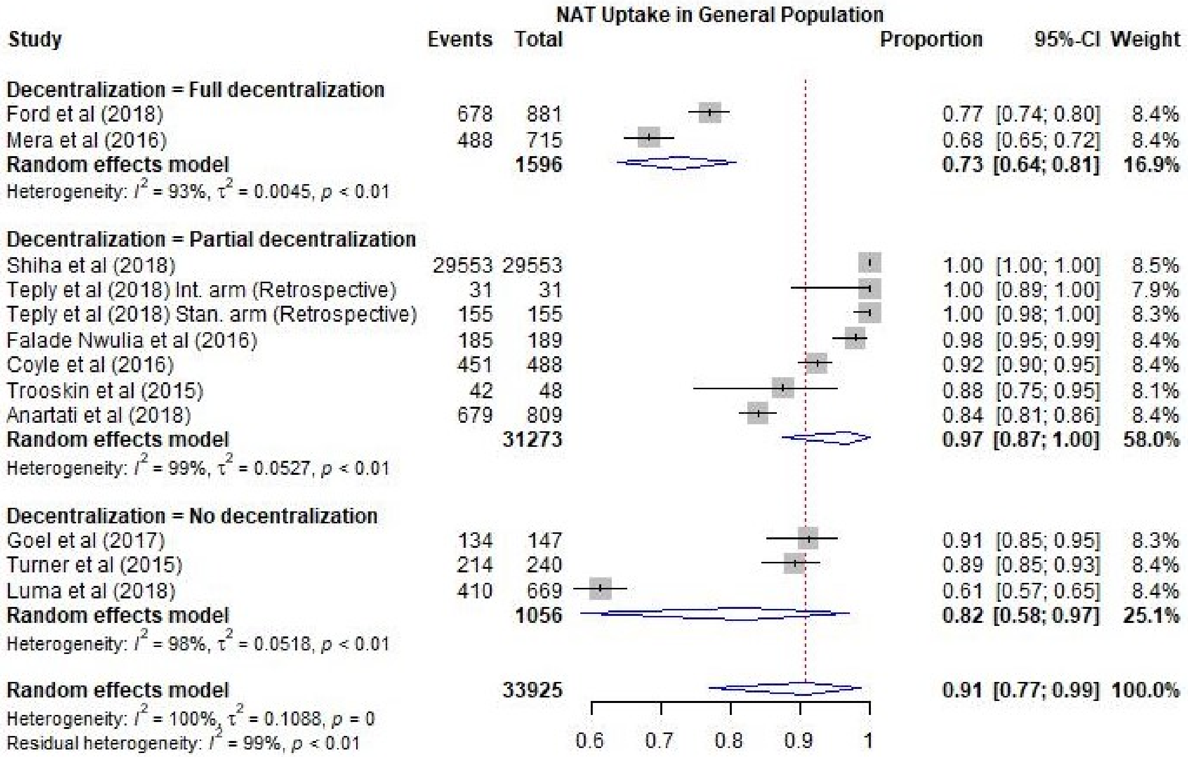
NAT Uptake in General Population
Study Events Total Proportion 95%-CI Weight
Decentralization = Full decentralization
Ford et al (2018) 678 881 0.77 [0.74; 0.80] 8.4%
Mera et al (2016) 488 715 0.68 [0.65; 0.72] 8.4%
Random effects model 1596 0.73 [0.64; 0.81] 16.9%
Heterogeneity: I^2 = 93%, τ^2 = 0.0045, p < 0.01
Decentralization = Partial decentralization
Shiha et al (2018) 29553 29553 1.00 [1.00; 1.00] 8.5%
Teply et al (2018) Int. arm (Retrospective) 31 31 1.00 [0.89; 1.00] 7.9%
Teply et al (2018) Stan. arm (Retrospective) 155 155 1.00 [0.98; 1.00] 8.3%
Falade Nwulia et al (2016) 185 189 0.98 [0.95; 0.99] 8.4%
Coyle et al (2016) 451 488 0.92 [0.90; 0.95] 8.4%
Trooskin et al (2015) 42 48 0.88 [0.75; 0.95] 8.1%
Anartati et al (2018) 679 809 0.84 [0.81; 0.86] 8.4%
Random effects model 31273 0.97 [0.87; 1.00] 58.0%
Heterogeneity: I^2 = 99%, τ^2 = 0.0527, p < 0.01
Decentralization = No decentralization
Goel et al (2017) 134 147 0.91 [0.85; 0.95] 8.3%
Turner et al (2015) 214 240 0.89 [0.85; 0.93] 8.4%
Luma et al (2018) 410 669 0.61 [0.57; 0.65] 8.4%
Random effects model 1056 0.82 [0.58; 0.97] 25.1%
Heterogeneity: I^2 = 98%, τ^2 = 0.0518, p < 0.01
Random effects model 33925 0.91 [0.77; 0.99] 100.0%
Heterogeneity: I^2 = 100%, τ^2 = 0.1088, p = 0
Residual heterogeneity: I^2 = 99%, p < 0.01
0.6 0.7 0.8 0.9 1

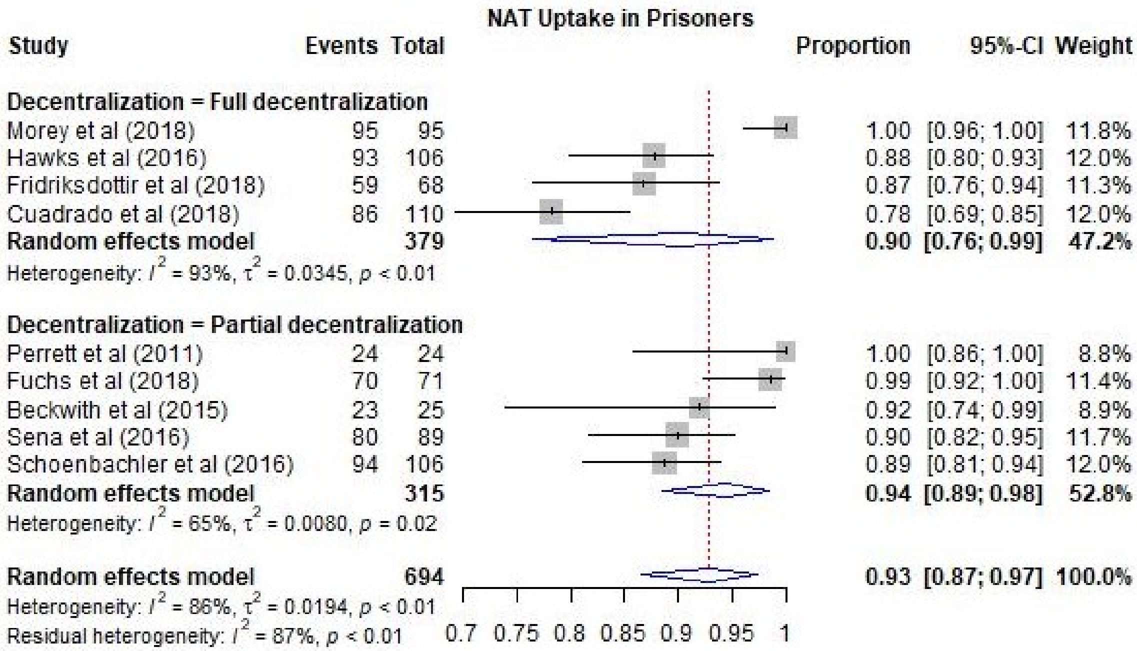
NAT Uptake in Prisoners
Study Events Total Proportion 95%-CI Weight
Decentralization = Full decentralization
Morey et al (2018) 95 95 1.00 [0.96; 1.00] 11.8%
Hawks et al (2016) 93 106 0.88 [0.80; 0.93] 12.0%
Fridriksdottir et al (2018) 59 68 0.87 [0.76; 0.94] 11.3%
Cuadrado et al (2018) 86 110 0.78 [0.69; 0.85] 12.0%
Random effects model 379 0.90 [0.76; 0.99] 47.2%
Heterogeneity: I^2 = 93%, τ^2 = 0.0345, p < 0.01
Decentralization = Partial decentralization
Perrett et al (2011) 24 24 1.00 [0.86; 1.00] 8.8%
Fuchs et al (2018) 70 71 0.99 [0.92; 1.00] 11.4%
Beckwith et al (2015) 23 25 0.92 [0.74; 0.99] 8.9%
Sena et al (2016) 80 89 0.90 [0.82; 0.95] 11.7%
Schoenbachler et al (2016) 94 106 0.89 [0.81; 0.94] 12.0%
Random effects model 315 0.94 [0.89; 0.98] 52.8%
Heterogeneity: I^2 = 65%, τ^2 = 0.0080, p = 0.02
Random effects model 694 0.93 [0.87; 0.97] 100.0%
Heterogeneity: I^2 = 86%, τ^2 = 0.0194, p < 0.01
Residual heterogeneity: I^2 = 87%, p < 0.01
0.7 0.75 0.8 0.85 0.9 0.95 1

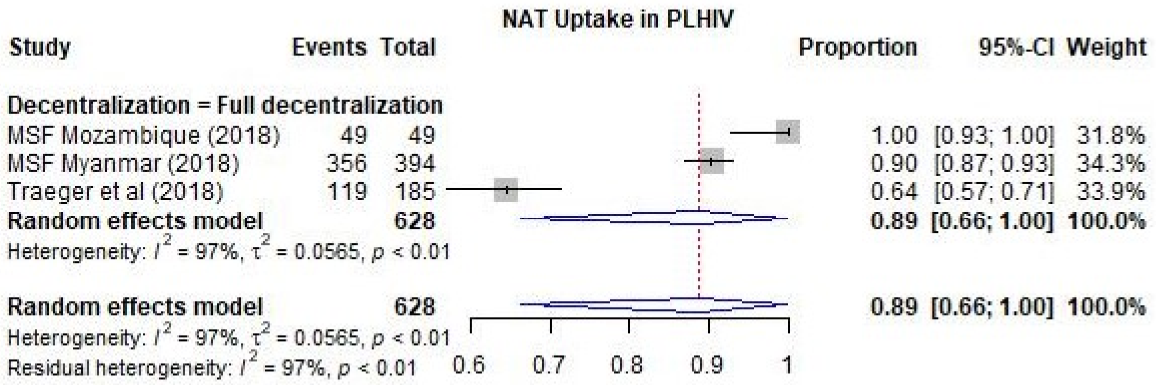
NAT Uptake in PLHIV
Study
Events Total
Proportion
95%-CI Weight
Decentralization = Full decentralization
MSF Mozambique (2018) 49 49
MSF Myanmar (2018) 356 394
Traeger et al (2018) 119 185
Random effects model 628
Heterogeneity: I^2 = 97%, τ^2 = 0.0565, p < 0.01
1.00 [0.93; 1.00] 31.8%
0.90 [0.87; 0.93] 34.3%
0.64 [0.57; 0.71] 33.9%
0.89 [0.66; 1.00] 100.0%
Random effects model 628
Heterogeneity: I^2 = 97%, τ^2 = 0.0565, p < 0.01
Residual heterogeneity: I^2 = 97%, p < 0.01
0.89 [0.66; 1.00] 100.0%
0.6
0.7
0.8
0.9
1

B.

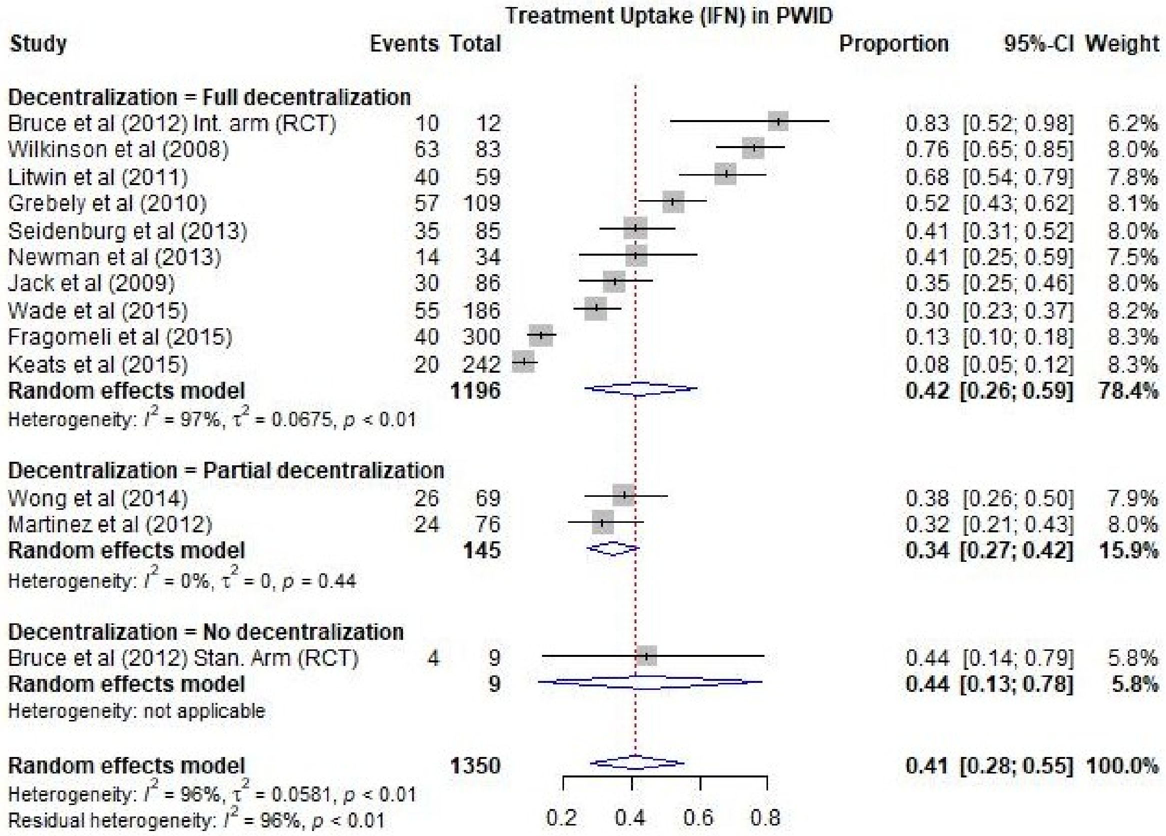
Treatment Uptake (IFN) in PWID
Study
Events Total
Proportion
95%-CI Weight
Decentralization = Full decentralization
Bruce et al (2012) Int. arm (RCT) 10 12
Wilkinson et al (2008) 63 83
Litwin et al (2011) 40 59
Grebely et al (2010) 57 109
Seidenburg et al (2013) 35 85
Newman et al (2013) 14 34
Jack et al (2009) 30 86
Wade et al (2015) 55 186
Fragomeli et al (2015) 40 300
Keats et al (2015) 20 242
Random effects model 1196
Heterogeneity: I^2 = 97%, τ^2 = 0.0675, p < 0.01
0.83 [0.52; 0.98] 6.2%
0.76 [0.65; 0.85] 8.0%
0.68 [0.54; 0.79] 7.8%
0.52 [0.43; 0.62] 8.1%
0.41 [0.31; 0.52] 8.0%
0.41 [0.25; 0.59] 7.5%
0.35 [0.25; 0.46] 8.0%
0.30 [0.23; 0.37] 8.2%
0.13 [0.10; 0.18] 8.3%
0.08 [0.05; 0.12] 8.3%
0.42 [0.26; 0.59] 78.4%
Decentralization = Partial decentralization
Wong et al (2014) 26 69
Martinez et al (2012) 24 76
Random effects model 145
Heterogeneity: I^2 = 0%, τ^2 = 0, p = 0.44
0.38 [0.26; 0.50] 7.9%
0.32 [0.21; 0.43] 8.0%
0.34 [0.27; 0.42] 15.9%
Decentralization = No decentralization
Bruce et al (2012) Stan. Arm (RCT) 4 9
Random effects model 9
Heterogeneity: not applicable
0.44 [0.14; 0.79] 5.8%
0.44 [0.13; 0.78] 5.8%
Random effects model 1350
Heterogeneity: I^2 = 96%, τ^2 = 0.0581, p < 0.01
Residual heterogeneity: I^2 = 96%, p < 0.01
0.41 [0.28; 0.55] 100.0%
0.2
0.4
0.6
0.8

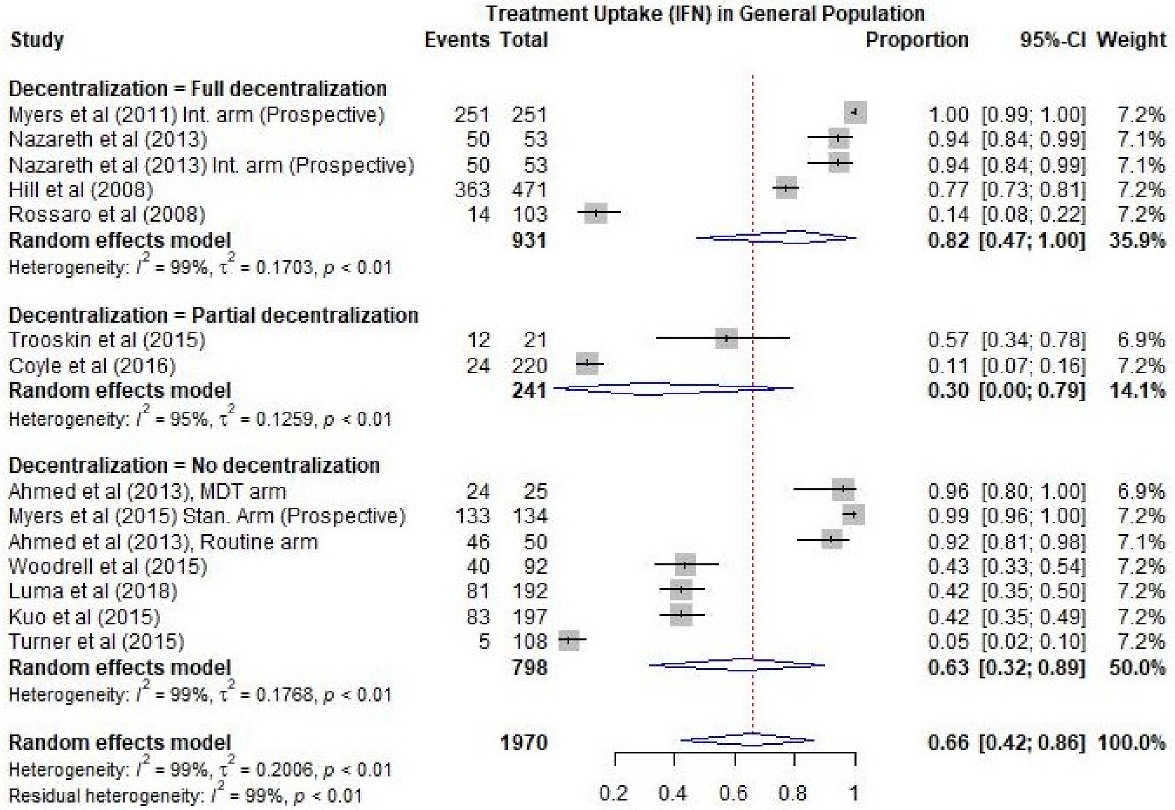
Treatment Uptake (IFN) in General Population
Study Events Total Proportion 95%-CI Weight
Decentralization = Full decentralization
Myers et al (2011) Int. arm (Prospective) 251 251 1.00 [0.99; 1.00] 7.2%
Nazareth et al (2013) 50 53 0.94 [0.84; 0.99] 7.1%
Nazareth et al (2013) Int. arm (Prospective) 50 53 0.94 [0.84; 0.99] 7.1%
Hill et al (2008) 363 471 0.77 [0.73; 0.81] 7.2%
Rossaro et al (2008) 14 103 0.14 [0.08; 0.22] 7.2%
Random effects model 931 0.82 [0.47; 1.00] 35.9%
Heterogeneity: I^2 = 99%, τ^2 = 0.1703, p < 0.01
Decentralization = Partial decentralization
Trooskin et al (2015) 12 21 0.57 [0.34; 0.78] 6.9%
Coyle et al (2016) 24 220 0.11 [0.07; 0.16] 7.2%
Random effects model 241 0.30 [0.00; 0.79] 14.1%
Heterogeneity: I^2 = 95%, τ^2 = 0.1259, p < 0.01
Decentralization = No decentralization
Ahmed et al (2013), MDT arm 24 25 0.96 [0.80; 1.00] 6.9%
Myers et al (2015) Stan. Arm (Prospective) 133 134 0.99 [0.96; 1.00] 7.2%
Ahmed et al (2013), Routine arm 46 50 0.92 [0.81; 0.98] 7.1%
Woodrell et al (2015) 40 92 0.43 [0.33; 0.54] 7.2%
Luma et al (2018) 81 192 0.42 [0.35; 0.50] 7.2%
Kuo et al (2015) 83 197 0.42 [0.35; 0.49] 7.2%
Turner et al (2015) 5 108 0.05 [0.02; 0.10] 7.2%
Random effects model 798 0.63 [0.32; 0.89] 50.0%
Heterogeneity: I^2 = 99%, τ^2 = 0.1768, p < 0.01
Random effects model 1970 0.66 [0.42; 0.86] 100.0%
Heterogeneity: I^2 = 99%, τ^2 = 0.2006, p < 0.01
Residual heterogeneity: I^2 = 99%, p < 0.01
0.2 0.4 0.6 0.8 1

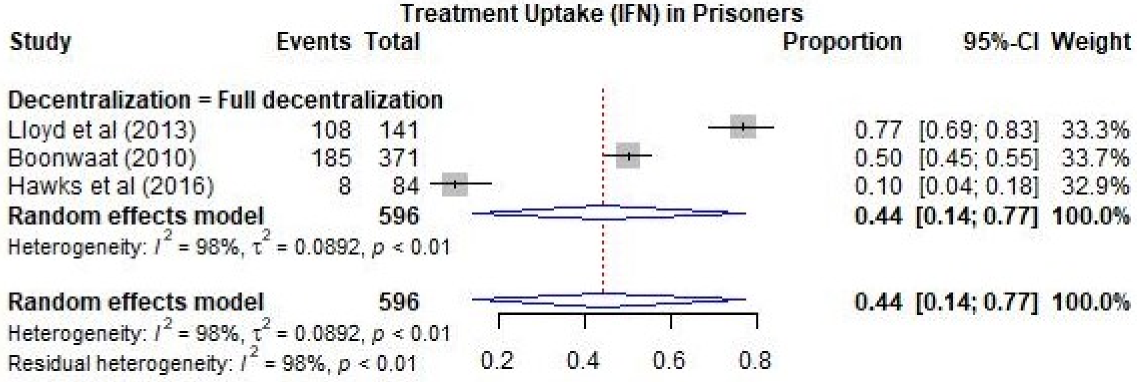
Treatment Uptake (IFN) in Prisoners
Study Events Total Proportion 95%-CI Weight
Decentralization = Full decentralization
Lloyd et al (2013) 108 141 0.77 [0.69; 0.83] 33.3%
Boonwaat (2010) 185 371 0.50 [0.45; 0.55] 33.7%
Hawks et al (2016) 8 84 0.10 [0.04; 0.18] 32.9%
Random effects model 596 0.44 [0.14; 0.77] 100.0%
Heterogeneity: I^2 = 98%, τ^2 = 0.0892, p < 0.01
Random effects model 596 0.44 [0.14; 0.77] 100.0%
Heterogeneity: I^2 = 98%, τ^2 = 0.0892, p < 0.01
Residual heterogeneity: I^2 = 98%, p < 0.01
0.2 0.4 0.6 0.8

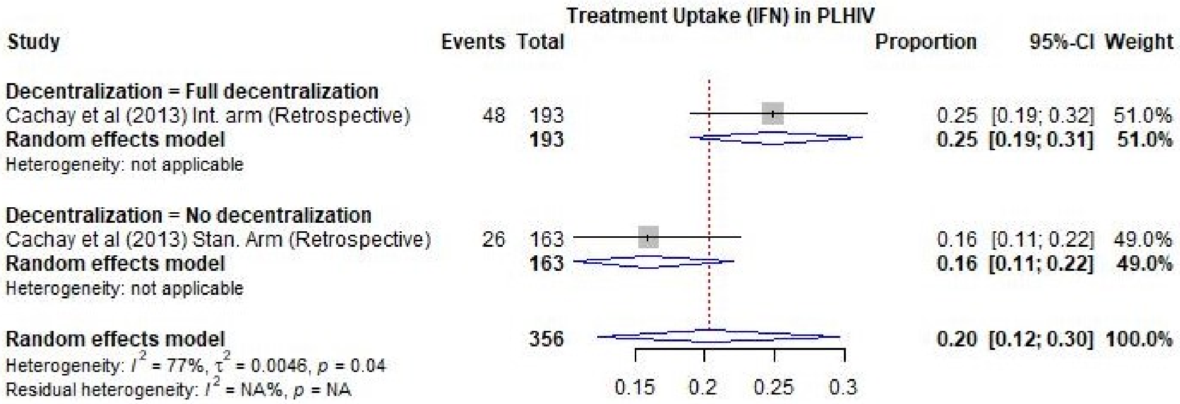
Treatment Uptake (IFN) in PLHIV
Study
Events Total
Proportion
95%-CI Weight
Decentralization = Full decentralization
Cachay et al (2013) Int. arm (Retrospective) 48 193 0.25 [0.19; 0.32] 51.0%
Random effects model 193 0.25 [0.19; 0.31] 51.0%
Heterogeneity: not applicable
Decentralization = No decentralization
Cachay et al (2013) Stan. Arm (Retrospective) 26 163 0.16 [0.11; 0.22] 49.0%
Random effects model 163 0.16 [0.11; 0.22] 49.0%
Heterogeneity: not applicable
Random effects model 356 0.20 [0.12; 0.30] 100.0%
Heterogeneity: I^2 = 77%, τ^2 = 0.0046, p = 0.04
Residual heterogeneity: I^2 = NA%, p = NA
0.15
0.2
0.25
0.3

C.

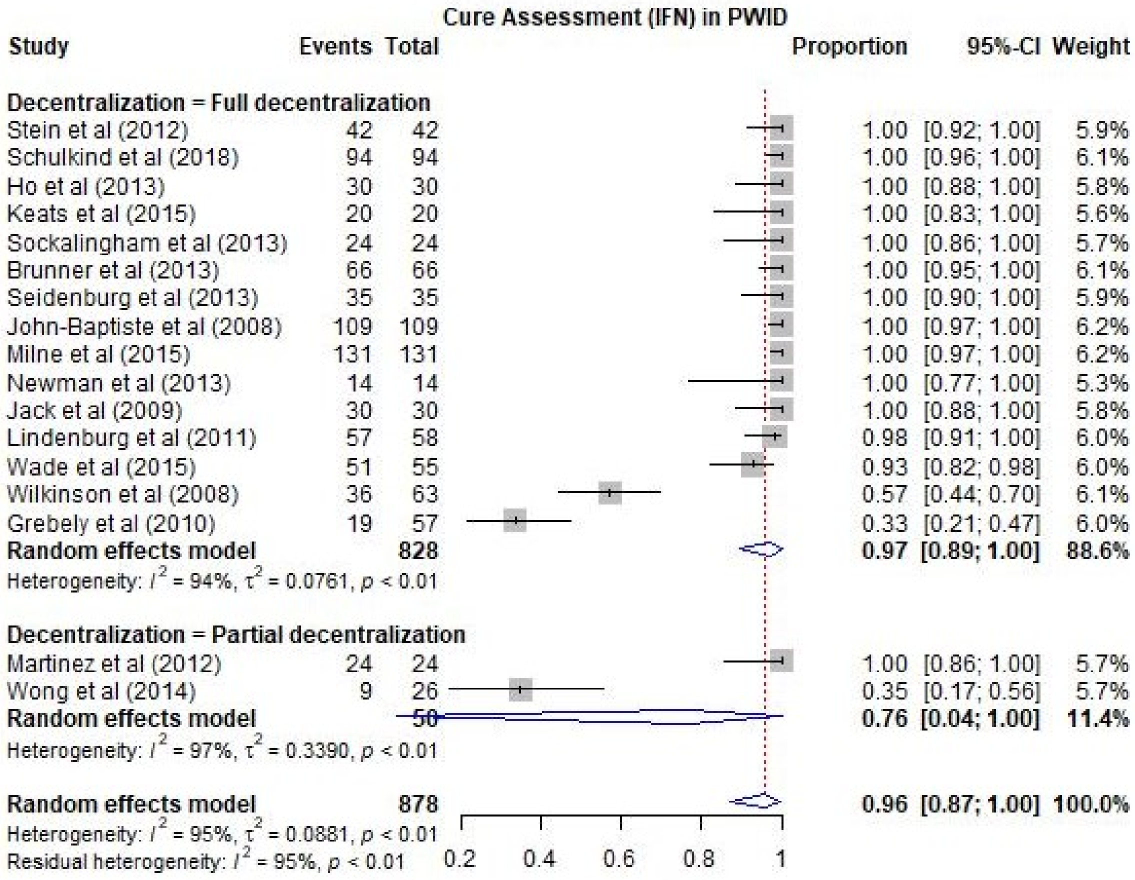
Cure Assessment (IFN) in PWID
Study
Events Total
Proportion
95%-CI Weight
Decentralization = Full decentralization
Stein et al (2012) 42 42 1.00 [0.92; 1.00] 5.9%
Schulkind et al (2018) 94 94 1.00 [0.96; 1.00] 6.1%
Ho et al (2013) 30 30 1.00 [0.88; 1.00] 5.8%
Keats et al (2015) 20 20 1.00 [0.83; 1.00] 5.6%
Sockalingham et al (2013) 24 24 1.00 [0.86; 1.00] 5.7%
Brunner et al (2013) 66 66 1.00 [0.95; 1.00] 6.1%
Seidenburg et al (2013) 35 35 1.00 [0.90; 1.00] 5.9%
John-Baptiste et al (2008) 109 109 1.00 [0.97; 1.00] 6.2%
Milne et al (2015) 131 131 1.00 [0.97; 1.00] 6.2%
Newman et al (2013) 14 14 1.00 [0.77; 1.00] 5.3%
Jack et al (2009) 30 30 1.00 [0.88; 1.00] 5.8%
Lindenburg et al (2011) 57 58 0.98 [0.91; 1.00] 6.0%
Wade et al (2015) 51 55 0.93 [0.82; 0.98] 6.0%
Wilkinson et al (2008) 36 63 0.57 [0.44; 0.70] 6.1%
Grebely et al (2010) 19 57 0.33 [0.21; 0.47] 6.0%
Random effects model 828 0.97 [0.89; 1.00] 88.6%
Heterogeneity: I^2 = 94%, τ^2 = 0.0761, p < 0.01
Decentralization = Partial decentralization
Martinez et al (2012) 24 24 1.00 [0.86; 1.00] 5.7%
Wong et al (2014) 9 26 0.35 [0.17; 0.56] 5.7%
Random effects model 50 0.76 [0.04; 1.00] 11.4%
Heterogeneity: I^2 = 97%, τ^2 = 0.3390, p < 0.01
Random effects model 878 0.96 [0.87; 1.00] 100.0%
Heterogeneity: I^2 = 95%, τ^2 = 0.0881, p < 0.01
Residual heterogeneity: I^2 = 95%, p < 0.01
0.2
0.4
0.6
0.8
1

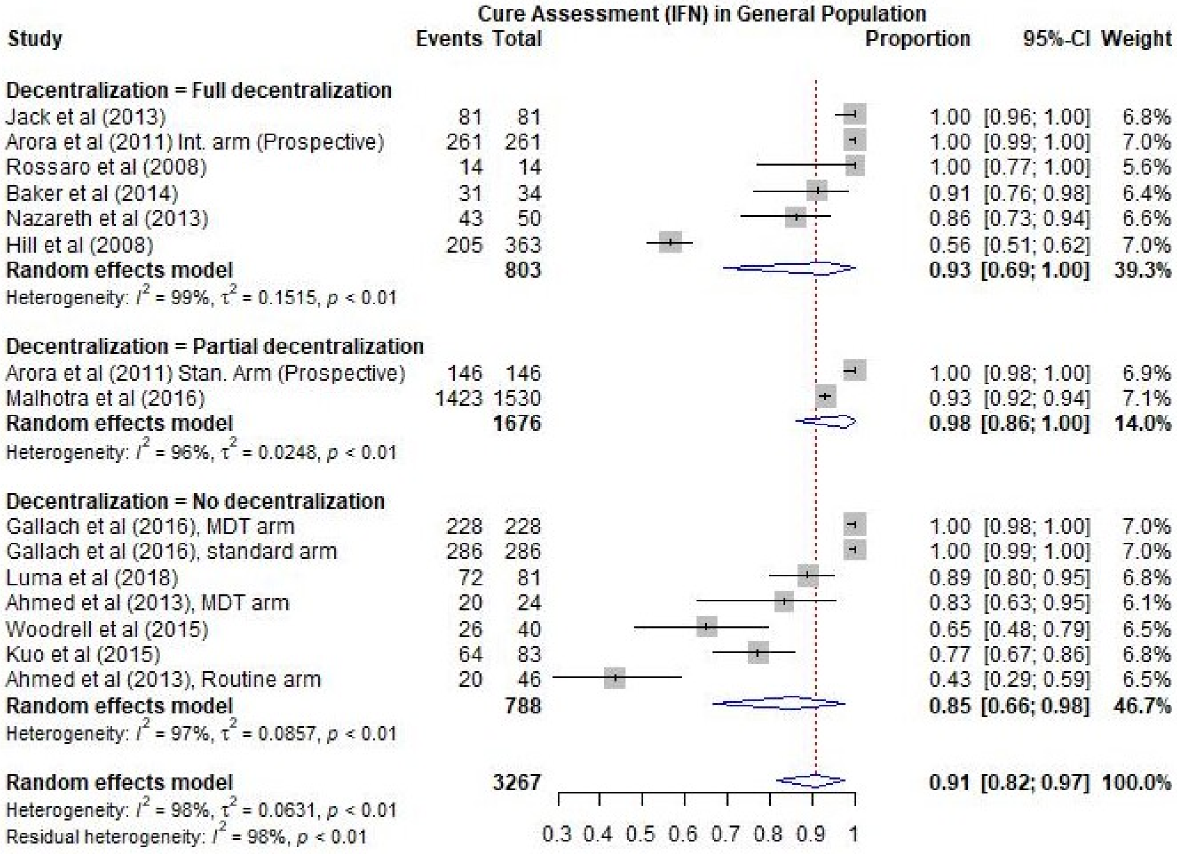
Cure Assessment (IFN) in General Population
Study Events Total Proportion 95%-CI Weight
Decentralization = Full decentralization
Jack et al (2013) 81 81 1.00 [0.96; 1.00] 6.8%
Arora et al (2011) Int. arm (Prospective) 261 261 1.00 [0.99; 1.00] 7.0%
Rossaro et al (2008) 14 14 1.00 [0.77; 1.00] 5.6%
Baker et al (2014) 31 34 0.91 [0.76; 0.98] 6.4%
Nazareth et al (2013) 43 50 0.86 [0.73; 0.94] 6.6%
Hill et al (2008) 205 363 0.56 [0.51; 0.62] 7.0%
Random effects model 803 0.93 [0.69; 1.00] 39.3%
Heterogeneity: I^2 = 99%, τ^2 = 0.1515, p < 0.01
Decentralization = Partial decentralization
Arora et al (2011) Stan. Arm (Prospective) 146 146 1.00 [0.98; 1.00] 6.9%
Malhotra et al (2016) 1423 1530 0.93 [0.92; 0.94] 7.1%
Random effects model 1676 0.98 [0.86; 1.00] 14.0%
Heterogeneity: I^2 = 96%, τ^2 = 0.0248, p < 0.01
Decentralization = No decentralization
Gallach et al (2016), MDT arm 228 228 1.00 [0.98; 1.00] 7.0%
Gallach et al (2016), standard arm 286 286 1.00 [0.99; 1.00] 7.0%
Luma et al (2018) 72 81 0.89 [0.80; 0.95] 6.8%
Ahmed et al (2013), MDT arm 20 24 0.83 [0.63; 0.95] 6.1%
Woodrell et al (2015) 26 40 0.65 [0.48; 0.79] 6.5%
Kuo et al (2015) 64 83 0.77 [0.67; 0.86] 6.8%
Ahmed et al (2013), Routine arm 20 46 0.43 [0.29; 0.59] 6.5%
Random effects model 788 0.85 [0.66; 0.98] 46.7%
Heterogeneity: I^2 = 97%, τ^2 = 0.0857, p < 0.01
Random effects model 3267 0.91 [0.82; 0.97] 100.0%
Heterogeneity: I^2 = 98%, τ^2 = 0.0631, p < 0.01
Residual heterogeneity: I^2 = 98%, p < 0.01
0.3 0.4 0.5 0.6 0.7 0.8 0.9 1

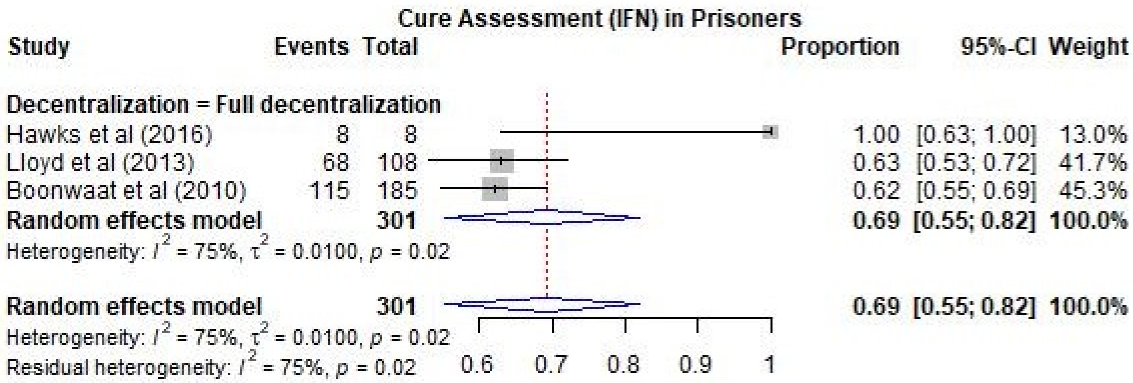
Cure Assessment (IFN) in Prisoners
Study Events Total Proportion 95%-CI Weight
Decentralization = Full decentralization
Hawks et al (2016) 8 8 1.00 [0.63; 1.00] 13.0%
Lloyd et al (2013) 68 108 0.63 [0.53; 0.72] 41.7%
Boonwaat et al (2010) 115 185 0.62 [0.55; 0.69] 45.3%
Random effects model 301 0.69 [0.55; 0.82] 100.0%
Heterogeneity: I^2 = 75%, τ^2 = 0.0100, p = 0.02
Random effects model 301 0.69 [0.55; 0.82] 100.0%
Heterogeneity: I^2 = 75%, τ^2 = 0.0100, p = 0.02
Residual heterogeneity: I^2 = 75%, p = 0.02
0.6 0.7 0.8 0.9 1

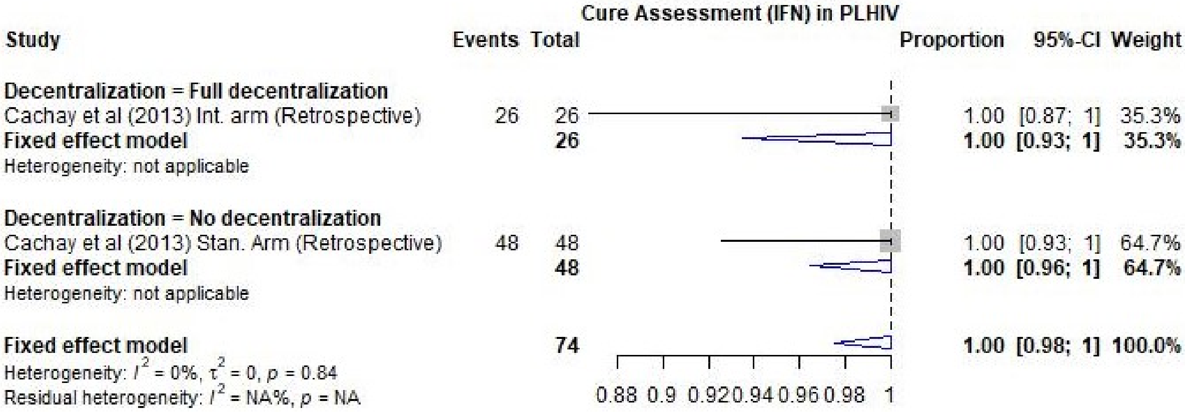
Cure Assessment (IFN) in PLHIV
Study
Events Total
Proportion 95%-CI Weight
Decentralization = Full decentralization
Cachay et al (2013) Int. arm (Retrospective) 26 26 1.00 [0.87; 1] 35.3%
Fixed effect model 26 1.00 [0.93; 1] 35.3%
Heterogeneity: not applicable
Decentralization = No decentralization
Cachay et al (2013) Stan. Arm (Retrospective) 48 48 1.00 [0.93; 1] 64.7%
Fixed effect model 48 1.00 [0.96; 1] 64.7%
Heterogeneity: not applicable
Fixed effect model 74 1.00 [0.98; 1] 100.0%
Heterogeneity: I^2 = 0%, τ^2 = 0, p = 0.84
Residual heterogeneity: I^2 = NA%, p = NA
0.88 0.9 0.92 0.94 0.96 0.98 1

D.

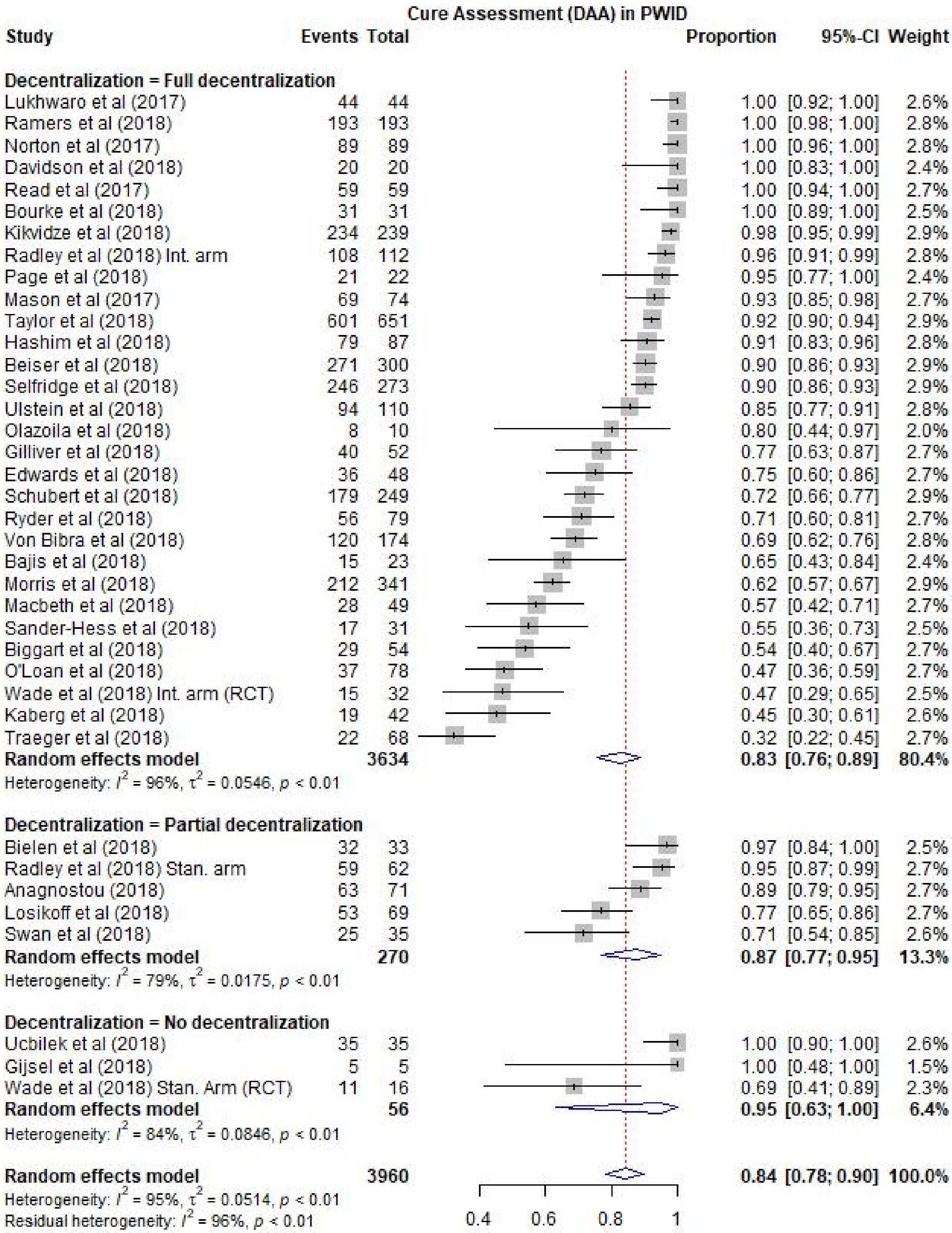
Cure Assessment (DAA) in PWID
Study Events Total Proportion 95%-CI Weight
Decentralization = Full decentralization
Lukhwaro et al (2017) 44 44 1.00 [0.92; 1.00] 2.6%
Ramers et al (2018) 193 193 1.00 [0.98; 1.00] 2.8%
Norton et al (2017) 89 89 1.00 [0.96; 1.00] 2.8%
Davidson et al (2018) 20 20 1.00 [0.83; 1.00] 2.4%
Read et al (2017) 59 59 1.00 [0.94; 1.00] 2.7%
Bourke et al (2018) 31 31 1.00 [0.89; 1.00] 2.5%
Kikvidze et al (2018) 234 239 0.98 [0.95; 0.99] 2.9%
Radley et al (2018) Int. arm 108 112 0.96 [0.91; 0.99] 2.8%
Page et al (2018) 21 22 0.95 [0.77; 1.00] 2.4%
Mason et al (2017) 69 74 0.93 [0.85; 0.98] 2.7%
Taylor et al (2018) 601 651 0.92 [0.90; 0.94] 2.9%
Hashim et al (2018) 79 87 0.91 [0.83; 0.96] 2.8%
Beiser et al (2018) 271 300 0.90 [0.86; 0.93] 2.9%
Selfridge et al (2018) 246 273 0.90 [0.86; 0.93] 2.9%
Ulstein et al (2018) 94 110 0.85 [0.77; 0.91] 2.8%
Olazoila et al (2018) 8 10 0.80 [0.44; 0.97] 2.0%
Gilliver et al (2018) 40 52 0.77 [0.63; 0.87] 2.7%
Edwards et al (2018) 36 48 0.75 [0.60; 0.86] 2.7%
Schubert et al (2018) 179 249 0.72 [0.66; 0.77] 2.9%
Ryder et al (2018) 56 79 0.71 [0.60; 0.81] 2.7%
Von Bibra et al (2018) 120 174 0.69 [0.62; 0.76] 2.8%
Bajis et al (2018) 15 23 0.65 [0.43; 0.84] 2.4%
Morris et al (2018) 212 341 0.62 [0.57; 0.67] 2.9%
Macbeth et al (2018) 28 49 0.57 [0.42; 0.71] 2.7%
Sander-Hess et al (2018) 17 31 0.55 [0.36; 0.73] 2.5%
Biggart et al (2018) 29 54 0.54 [0.40; 0.67] 2.7%
O'Loan et al (2018) 37 78 0.47 [0.36; 0.59] 2.7%
Wade et al (2018) Int. arm (RCT) 15 32 0.47 [0.29; 0.65] 2.5%
Kaberg et al (2018) 19 42 0.45 [0.30; 0.61] 2.6%
Traeger et al (2018) 22 68 0.32 [0.22; 0.45] 2.7%
Random effects model 3634 0.83 [0.76; 0.89] 80.4%
Heterogeneity: I^2 = 96%, τ^2 = 0.0546, p < 0.01
Decentralization = Partial decentralization
Bielen et al (2018) 32 33 0.97 [0.84; 1.00] 2.5%
Radley et al (2018) Stan. arm 59 62 0.95 [0.87; 0.99] 2.7%
Anagnostou (2018) 63 71 0.89 [0.79; 0.95] 2.7%
Losikoff et al (2018) 53 69 0.77 [0.65; 0.86] 2.7%
Swan et al (2018) 25 35 0.71 [0.54; 0.85] 2.6%
Random effects model 270 0.87 [0.77; 0.95] 13.3%
Heterogeneity: I^2 = 79%, τ^2 = 0.0175, p < 0.01
Decentralization = No decentralization
Ucbilek et al (2018) 35 35 1.00 [0.90; 1.00] 2.6%
Gijsel et al (2018) 5 5 1.00 [0.48; 1.00] 1.5%
Wade et al (2018) Stan. Arm (RCT) 11 16 0.69 [0.41; 0.89] 2.3%
Random effects model 56 0.95 [0.63; 1.00] 6.4%
Heterogeneity: I^2 = 84%, τ^2 = 0.0846, p < 0.01
Random effects model 3960 0.84 [0.78; 0.90] 100.0%
Heterogeneity: I^2 = 95%, τ^2 = 0.0514, p < 0.01
Residual heterogeneity: I^2 = 96%, p < 0.01
0.4 0.6 0.8 1

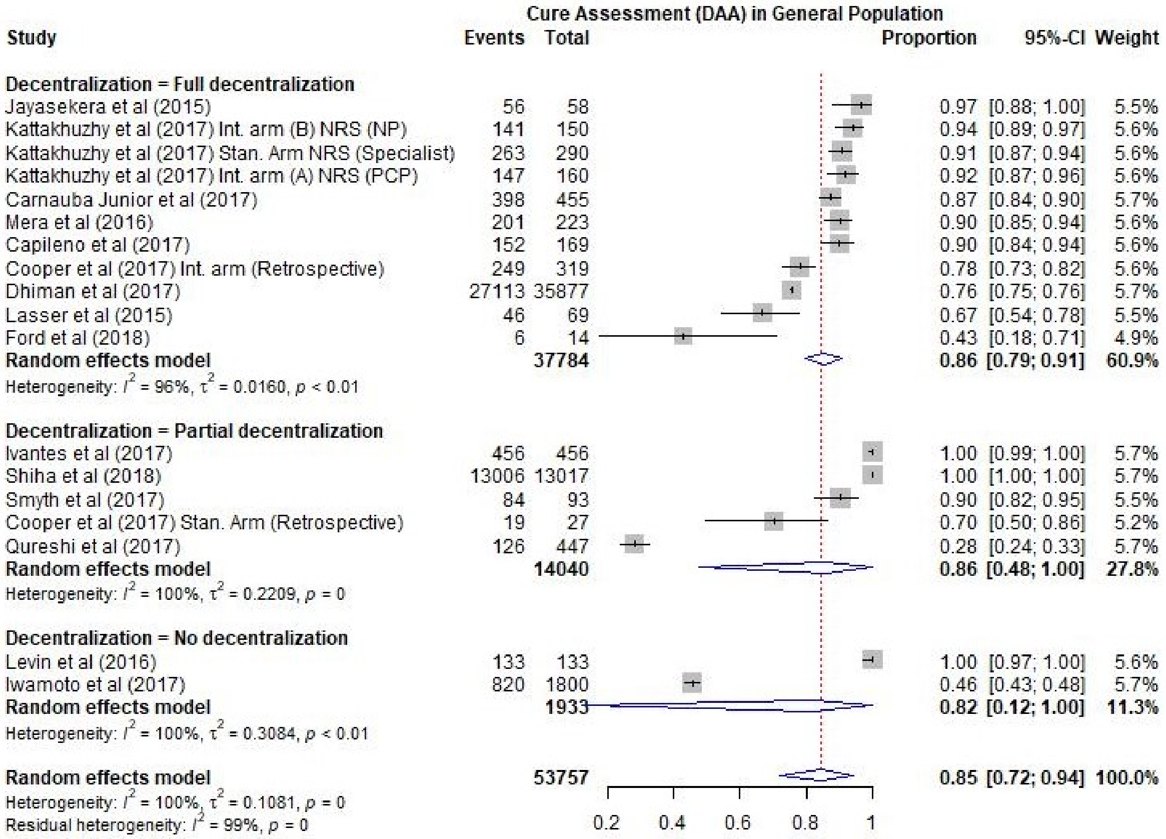
Cure Assessment (DAA) in General Population
Study Events Total Proportion 95%-CI Weight
Decentralization = Full decentralization
Jayasekera et al (2015) 56 58 0.97 [0.88; 1.00] 5.5%
Kattakhuzhy et al (2017) Int. arm (B) NRS (NP) 141 150 0.94 [0.89; 0.97] 5.6%
Kattakhuzhy et al (2017) Stan. Arm NRS (Specialist) 263 290 0.91 [0.87; 0.94] 5.6%
Kattakhuzhy et al (2017) Int. arm (A) NRS (PCP) 147 160 0.92 [0.87; 0.96] 5.6%
Carnauba Junior et al (2017) 398 455 0.87 [0.84; 0.90] 5.7%
Mera et al (2016) 201 223 0.90 [0.85; 0.94] 5.6%
Capileno et al (2017) 152 169 0.90 [0.84; 0.94] 5.6%
Cooper et al (2017) Int. arm (Retrospective) 249 319 0.78 [0.73; 0.82] 5.6%
Dhiman et al (2017) 27113 35877 0.76 [0.75; 0.76] 5.7%
Lasser et al (2015) 46 69 0.67 [0.54; 0.78] 5.5%
Ford et al (2018) 6 14 0.43 [0.18; 0.71] 4.9%
Random effects model 37784 0.86 [0.79; 0.91] 60.9%
Heterogeneity: I^2 = 96%, τ^2 = 0.0160, p < 0.01
Decentralization = Partial decentralization
Ivantes et al (2017) 456 456 1.00 [0.99; 1.00] 5.7%
Shiha et al (2018) 13006 13017 1.00 [1.00; 1.00] 5.7%
Smyth et al (2017) 84 93 0.90 [0.82; 0.95] 5.5%
Cooper et al (2017) Stan. Arm (Retrospective) 19 27 0.70 [0.50; 0.86] 5.2%
Qureshi et al (2017) 126 447 0.28 [0.24; 0.33] 5.7%
Random effects model 14040 0.86 [0.48; 1.00] 27.8%
Heterogeneity: I^2 = 100%, τ^2 = 0.2209, p = 0
Decentralization = No decentralization
Levin et al (2016) 133 133 1.00 [0.97; 1.00] 5.6%
Iwamoto et al (2017) 820 1800 0.46 [0.43; 0.48] 5.7%
Random effects model 1933 0.82 [0.12; 1.00] 11.3%
Heterogeneity: I^2 = 100%, τ^2 = 0.3084, p < 0.01
Random effects model 53757 0.85 [0.72; 0.94] 100.0%
Heterogeneity: I^2 = 100%, τ^2 = 0.1081, p = 0
Residual heterogeneity: I^2 = 99%, p = 0
0.2 0.4 0.6 0.8 1

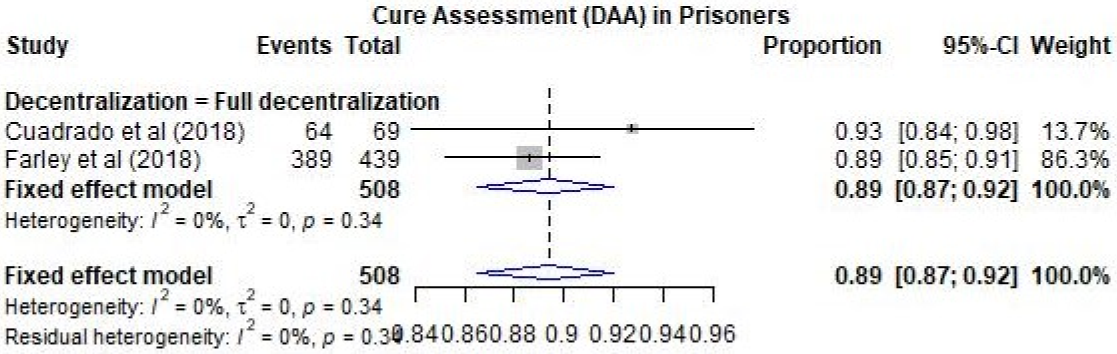
Cure Assessment (DAA) in Prisoners
Study Events Total Proportion 95%-CI Weight
Decentralization = Full decentralization
Cuadrado et al (2018) 64 69 0.93 [0.84; 0.98] 13.7%
Farley et al (2018) 389 439 0.89 [0.85; 0.91] 86.3%
Fixed effect model 508 0.89 [0.87; 0.92] 100.0%
Heterogeneity: I^2 = 0%, τ^2 = 0, p = 0.34
Fixed effect model 508 0.89 [0.87; 0.92] 100.0%
Heterogeneity: I^2 = 0%, τ^2 = 0, p = 0.34
Residual heterogeneity: I^2 = 0%, p = 0.34
0.84 0.86 0.88 0.9 0.92 0.94 0.96

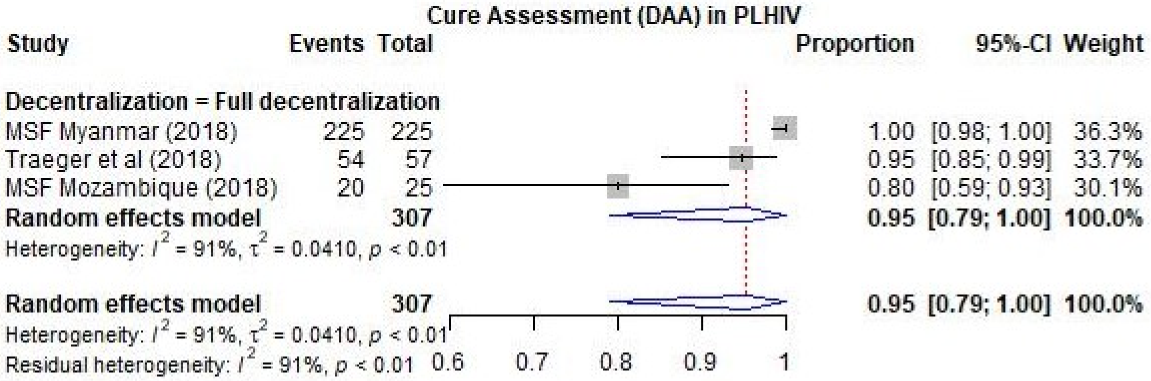
Cure Assessment (DAA) in PLHIV
Study Events Total Proportion 95%-CI Weight
Decentralization = Full decentralization
MSF Myanmar (2018) 225 225 1.00 [0.98; 1.00] 36.3%
Traeger et al (2018) 54 57 0.95 [0.85; 0.99] 33.7%
MSF Mozambique (2018) 20 25 0.80 [0.59; 0.93] 30.1%
Random effects model 307 0.95 [0.79; 1.00] 100.0%
Heterogeneity: I^2 = 91%, τ^2 = 0.0410, p < 0.01
Random effects model 307 0.95 [0.79; 1.00] 100.0%
Heterogeneity: I^2 = 91%, τ^2 = 0.0410, p < 0.01
Residual heterogeneity: I^2 = 91%, p < 0.01
0.6 0.7 0.8 0.9 1

E.

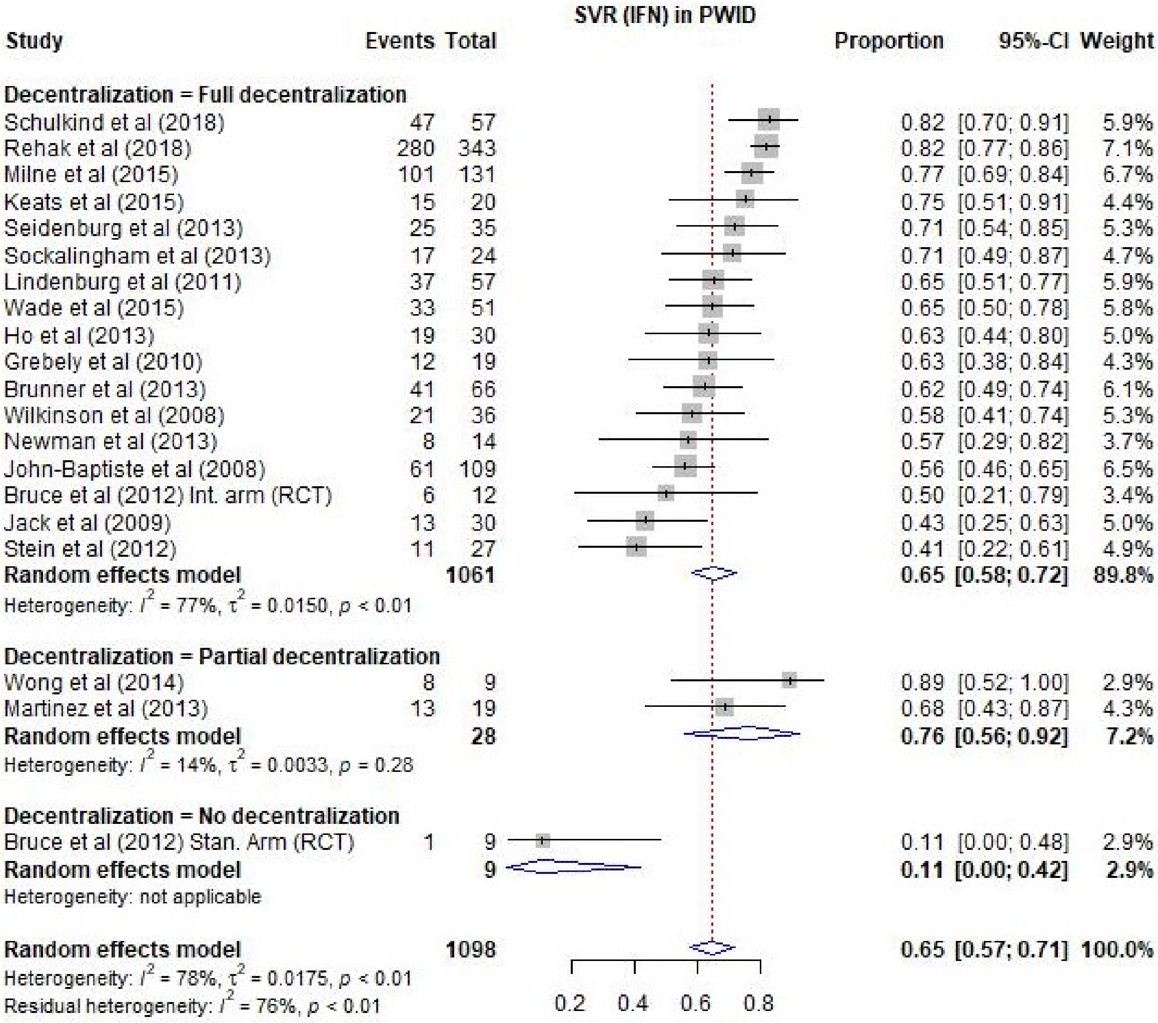
SVR (IFN) in PWID
Study Events Total Proportion 95%-CI Weight
Decentralization = Full decentralization
Schulkind et al (2018) 47 57 0.82 [0.70; 0.91] 5.9%
Rehak et al (2018) 280 343 0.82 [0.77; 0.86] 7.1%
Milne et al (2015) 101 131 0.77 [0.69; 0.84] 6.7%
Keats et al (2015) 15 20 0.75 [0.51; 0.91] 4.4%
Seidenburg et al (2013) 25 35 0.71 [0.54; 0.85] 5.3%
Sockalingham et al (2013) 17 24 0.71 [0.49; 0.87] 4.7%
Lindenburg et al (2011) 37 57 0.65 [0.51; 0.77] 5.9%
Wade et al (2015) 33 51 0.65 [0.50; 0.78] 5.8%
Ho et al (2013) 19 30 0.63 [0.44; 0.80] 5.0%
Grebely et al (2010) 12 19 0.63 [0.38; 0.84] 4.3%
Brunner et al (2013) 41 66 0.62 [0.49; 0.74] 6.1%
Wilkinson et al (2008) 21 36 0.58 [0.41; 0.74] 5.3%
Newman et al (2013) 8 14 0.57 [0.29; 0.82] 3.7%
John-Baptiste et al (2008) 61 109 0.56 [0.46; 0.65] 6.5%
Bruce et al (2012) Int. arm (RCT) 6 12 0.50 [0.21; 0.79] 3.4%
Jack et al (2009) 13 30 0.43 [0.25; 0.63] 5.0%
Stein et al (2012) 11 27 0.41 [0.22; 0.61] 4.9%
Random effects model 1061 0.65 [0.58; 0.72] 89.8%
Heterogeneity: I^2 = 77%, τ^2 = 0.0150, p < 0.01
Decentralization = Partial decentralization
Wong et al (2014) 8 9 0.89 [0.52; 1.00] 2.9%
Martinez et al (2013) 13 19 0.68 [0.43; 0.87] 4.3%
Random effects model 28 0.76 [0.56; 0.92] 7.2%
Heterogeneity: I^2 = 14%, τ^2 = 0.0033, p = 0.28
Decentralization = No decentralization
Bruce et al (2012) Stan. Arm (RCT) 1 9 0.11 [0.00; 0.48] 2.9%
Random effects model 9 0.11 [0.00; 0.42] 2.9%
Heterogeneity: not applicable
Random effects model 1098 0.65 [0.57; 0.71] 100.0%
Heterogeneity: I^2 = 78%, τ^2 = 0.0175, p < 0.01
Residual heterogeneity: I^2 = 76%, p < 0.01
0.2 0.4 0.6 0.8

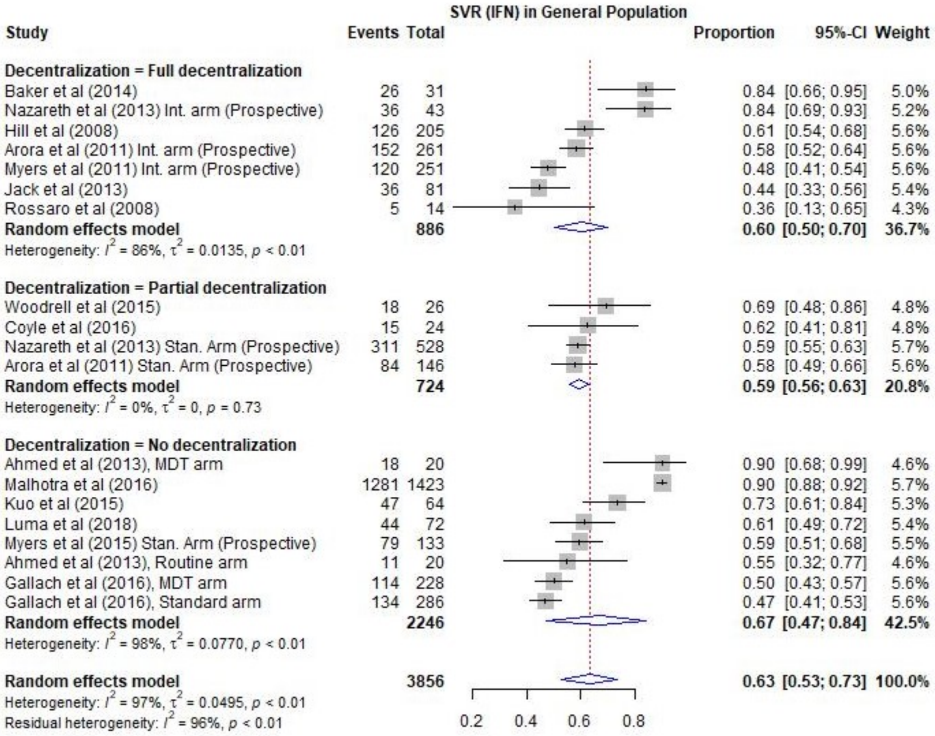
SVR (IFN) in General Population
Study Events Total Proportion 95%-CI Weight
Decentralization = Full decentralization
Baker et al (2014) 26 31 0.84 [0.66; 0.95] 5.0%
Nazareth et al (2013) Int. arm (Prospective) 36 43 0.84 [0.69; 0.93] 5.2%
Hill et al (2008) 126 205 0.61 [0.54; 0.68] 5.6%
Arora et al (2011) Int. arm (Prospective) 152 261 0.58 [0.52; 0.64] 5.6%
Myers et al (2011) Int. arm (Prospective) 120 251 0.48 [0.41; 0.54] 5.6%
Jack et al (2013) 36 81 0.44 [0.33; 0.56] 5.4%
Rossaro et al (2008) 5 14 0.36 [0.13; 0.65] 4.3%
Random effects model 886 0.60 [0.50; 0.70] 36.7%
Heterogeneity: I^2 = 86%, τ^2 = 0.0135, p < 0.01
Decentralization = Partial decentralization
Woodrell et al (2015) 18 26 0.69 [0.48; 0.86] 4.8%
Coyle et al (2016) 15 24 0.62 [0.41; 0.81] 4.8%
Nazareth et al (2013) Stan. Arm (Prospective) 311 528 0.59 [0.55; 0.63] 5.7%
Arora et al (2011) Stan. Arm (Prospective) 84 146 0.58 [0.49; 0.66] 5.6%
Random effects model 724 0.59 [0.56; 0.63] 20.8%
Heterogeneity: I^2 = 0%, τ^2 = 0, p = 0.73
Decentralization = No decentralization
Ahmed et al (2013), MDT arm 18 20 0.90 [0.68; 0.99] 4.6%
Malhotra et al (2016) 1281 1423 0.90 [0.88; 0.92] 5.7%
Kuo et al (2015) 47 64 0.73 [0.61; 0.84] 5.3%
Luma et al (2018) 44 72 0.61 [0.49; 0.72] 5.4%
Myers et al (2015) Stan. Arm (Prospective) 79 133 0.59 [0.51; 0.68] 5.5%
Ahmed et al (2013), Routine arm 11 20 0.55 [0.32; 0.77] 4.6%
Gallach et al (2016), MDT arm 114 228 0.50 [0.43; 0.57] 5.6%
Gallach et al (2016), Standard arm 134 286 0.47 [0.41; 0.53] 5.6%
Random effects model 2246 0.67 [0.47; 0.84] 42.5%
Heterogeneity: I^2 = 98%, τ^2 = 0.0770, p < 0.01
Random effects model 3856 0.63 [0.53; 0.73] 100.0%
Heterogeneity: I^2 = 97%, τ^2 = 0.0495, p < 0.01
Residual heterogeneity: I^2 = 96%, p < 0.01
0.2 0.4 0.6 0.8

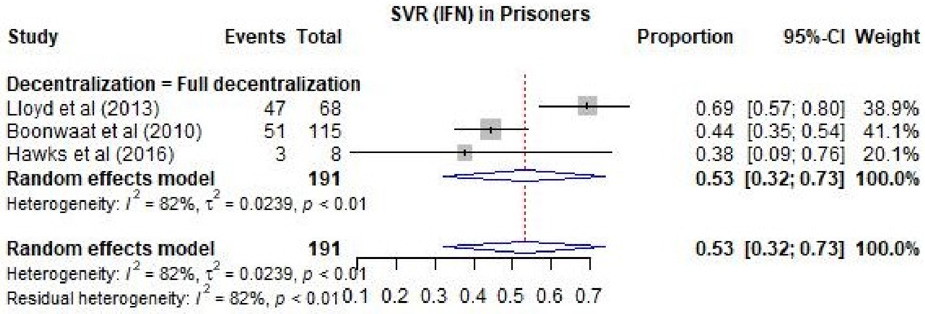
SVR (IFN) in Prisoners
Study Events Total Proportion 95%-CI Weight
Decentralization = Full decentralization
Lloyd et al (2013) 47 68 0.69 [0.57; 0.80] 38.9%
Boonwaat et al (2010) 51 115 0.44 [0.35; 0.54] 41.1%
Hawks et al (2016) 3 8 0.38 [0.09; 0.76] 20.1%
Random effects model 191 0.53 [0.32; 0.73] 100.0%
Heterogeneity: I^2 = 82%, τ^2 = 0.0239, p < 0.01
Random effects model 191 0.53 [0.32; 0.73] 100.0%
Heterogeneity: I^2 = 82%, τ^2 = 0.0239, p < 0.01
Residual heterogeneity: I^2 = 82%, p < 0.01
0.1 0.2 0.3 0.4 0.5 0.6 0.7

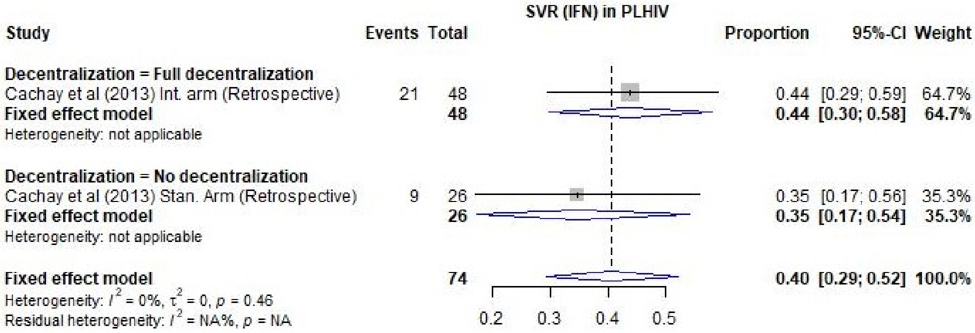
SVR (IFN) in PLHIV
Study Events Total Proportion 95%-CI Weight
Decentralization = Full decentralization
Cachay et al (2013) Int. arm (Retrospective) 21 48 0.44 [0.29; 0.59] 64.7%
Fixed effect model 48 0.44 [0.30; 0.58] 64.7%
Heterogeneity: not applicable
Decentralization = No decentralization
Cachay et al (2013) Stan. Arm (Retrospective) 9 26 0.35 [0.17; 0.56] 35.3%
Fixed effect model 26 0.35 [0.17; 0.54] 35.3%
Heterogeneity: not applicable
Fixed effect model 74 0.40 [0.29; 0.52] 100.0%
Heterogeneity: I^2 = 0%, τ^2 = 0, p = 0.46
Residual heterogeneity: I^2 = NA%, p = NA
0.2 0.3 0.4 0.5

Supplementary figure 2 A-C: Impact of decentralization and integration on SVR 12 for DAA, for A) PWID, B) the general population, and C) people in prisons.

A.

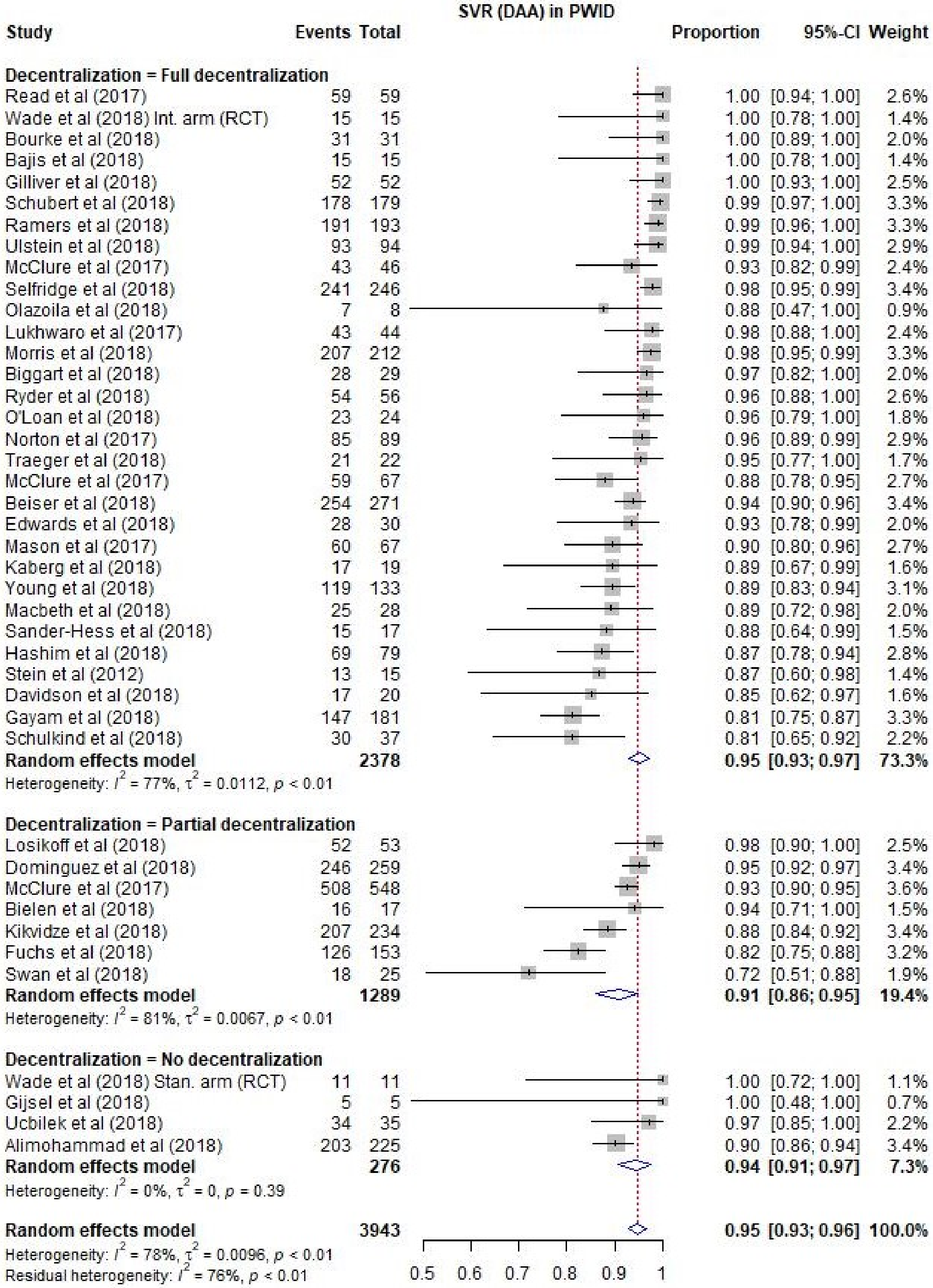

B.

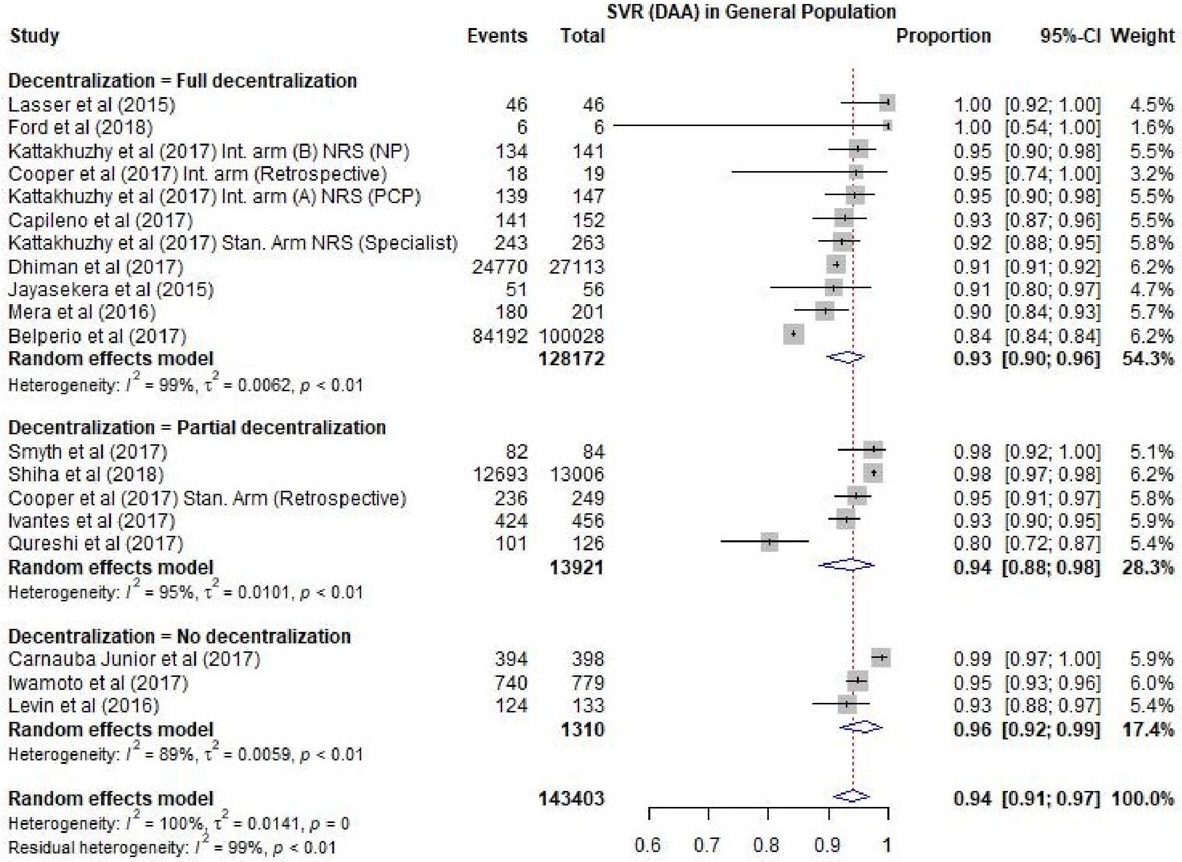
SVR (DAA) in General Population
Study
Events
Total
Proportion
95%-CI
Weight
Decentralization = Full decentralization
Lasser et al (2015) 46 46 1.00 [0.92; 1.00] 4.5%
Ford et al (2018) 6 6 1.00 [0.54; 1.00] 1.6%
Kattakhuzhy et al (2017) Int. arm (B) NRS (NP) 134 141 0.95 [0.90; 0.98] 5.5%
Cooper et al (2017) Int. arm (Retrospective) 18 19 0.95 [0.74; 1.00] 3.2%
Kattakhuzhy et al (2017) Int. arm (A) NRS (PCP) 139 147 0.95 [0.90; 0.98] 5.5%
Capileno et al (2017) 141 152 0.93 [0.87; 0.96] 5.5%
Kattakhuzhy et al (2017) Stan. Arm NRS (Specialist) 243 263 0.92 [0.88; 0.95] 5.8%
Dhiman et al (2017) 24770 27113 0.91 [0.91; 0.92] 6.2%
Jayasekera et al (2015) 51 56 0.91 [0.80; 0.97] 4.7%
Mera et al (2016) 180 201 0.90 [0.84; 0.93] 5.7%
Belperio et al (2017) 84192 100028 0.84 [0.84; 0.84] 6.2%
Random effects model 128172 0.93 [0.90; 0.96] 54.3%
Heterogeneity: I^2 = 99%, τ^2 = 0.0062, p < 0.01
Decentralization = Partial decentralization
Smyth et al (2017) 82 84 0.98 [0.92; 1.00] 5.1%
Shiha et al (2018) 12693 13006 0.98 [0.97; 0.98] 6.2%
Cooper et al (2017) Stan. Arm (Retrospective) 236 249 0.95 [0.91; 0.97] 5.8%
Ivantes et al (2017) 424 456 0.93 [0.90; 0.95] 5.9%
Qureshi et al (2017) 101 126 0.80 [0.72; 0.87] 5.4%
Random effects model 13921 0.94 [0.88; 0.98] 28.3%
Heterogeneity: I^2 = 95%, τ^2 = 0.0101, p < 0.01
Decentralization = No decentralization
Carnauba Junior et al (2017) 394 398 0.99 [0.97; 1.00] 5.9%
Iwamoto et al (2017) 740 779 0.95 [0.93; 0.96] 6.0%
Levin et al (2016) 124 133 0.93 [0.88; 0.97] 5.4%
Random effects model 1310 0.96 [0.92; 0.99] 17.4%
Heterogeneity: I^2 = 89%, τ^2 = 0.0059, p < 0.01
Random effects model 143403 0.94 [0.91; 0.97] 100.0%
Heterogeneity: I^2 = 100%, τ^2 = 0.0141, p = 0
Residual heterogeneity: I^2 = 99%, p < 0.01
0.6 0.7 0.8 0.9 1

C.

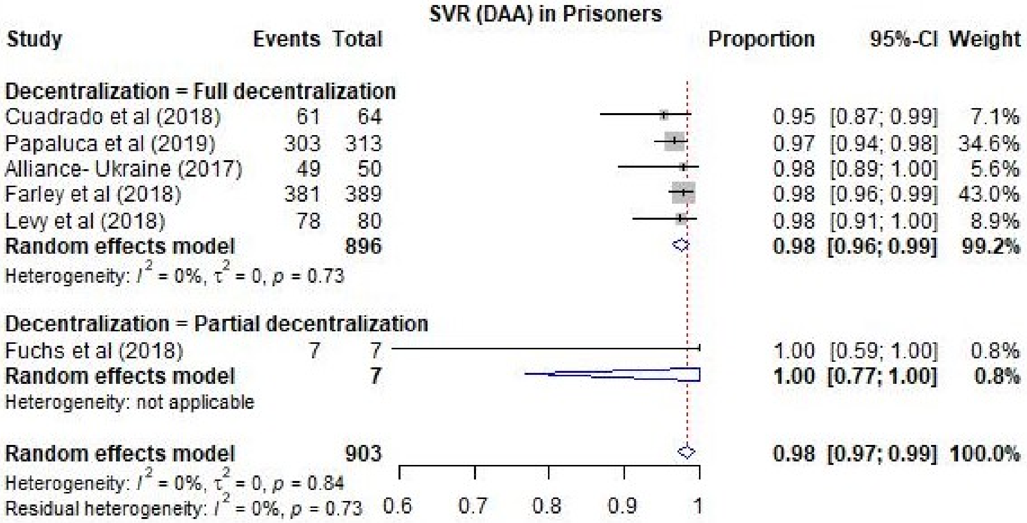
SVR (DAA) in Prisoners
Study
Events
Total
Proportion
95%-CI
Weight
Decentralization = Full decentralization
Cuadrado et al (2018) 61 64 0.95 [0.87; 0.99] 7.1%
Papaluca et al (2019) 303 313 0.97 [0.94; 0.98] 34.6%
Alliance- Ukraine (2017) 49 50 0.98 [0.89; 1.00] 5.6%
Farley et al (2018) 381 389 0.98 [0.96; 0.99] 43.0%
Levy et al (2018) 78 80 0.98 [0.91; 1.00] 8.9%
Random effects model 896 0.98 [0.96; 0.99] 99.2%
Heterogeneity: I^2 = 0%, τ^2 = 0, p = 0.73
Decentralization = Partial decentralization
Fuchs et al (2018) 7 7 1.00 [0.59; 1.00] 0.8%
Random effects model 7 1.00 [0.77; 1.00] 0.8%
Heterogeneity: not applicable
Random effects model 903 0.98 [0.97; 0.99] 100.0%
Heterogeneity: I^2 = 0%, τ^2 = 0, p = 0.84
Residual heterogeneity: I^2 = 0%, p = 0.73
0.6 0.7 0.8 0.9 1

Supplementary figures 3 A – D. I Among people living with HIV, impact of decentralization and integration on (A) testing uptake, (B) DAA treatment uptake, and (C) SVR 12 for DAAs, and (D) impact of task-shifting to non-specialists on SVR12 for people living with HIV.

A

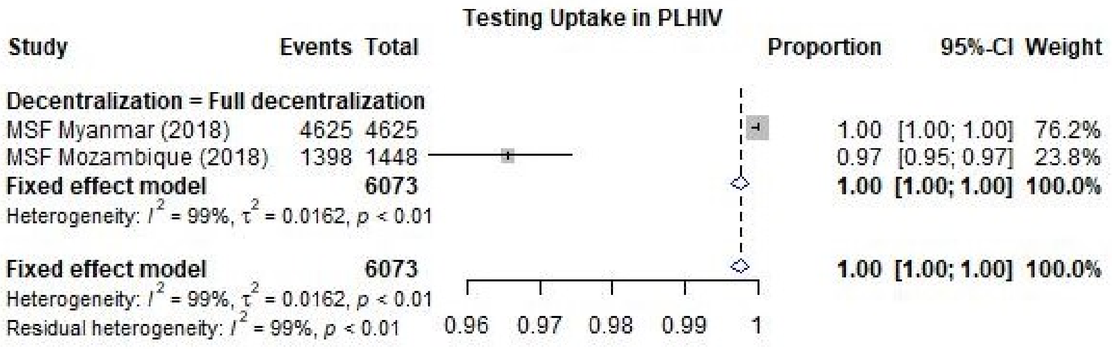

B

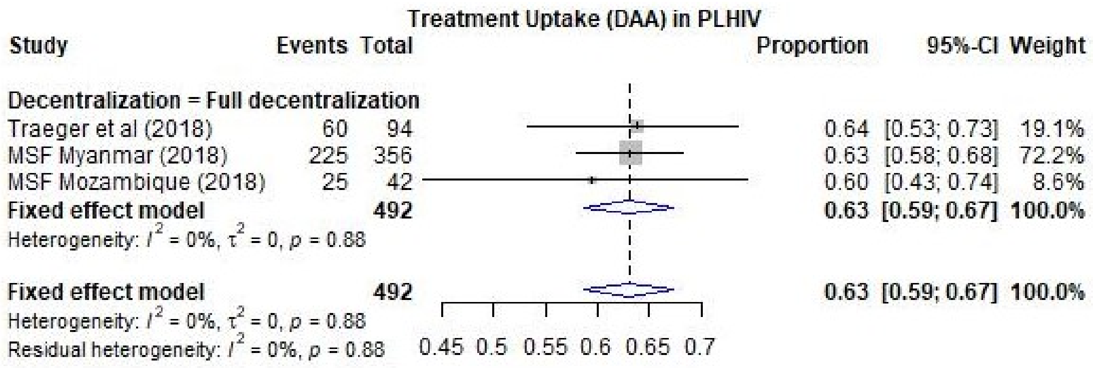

C

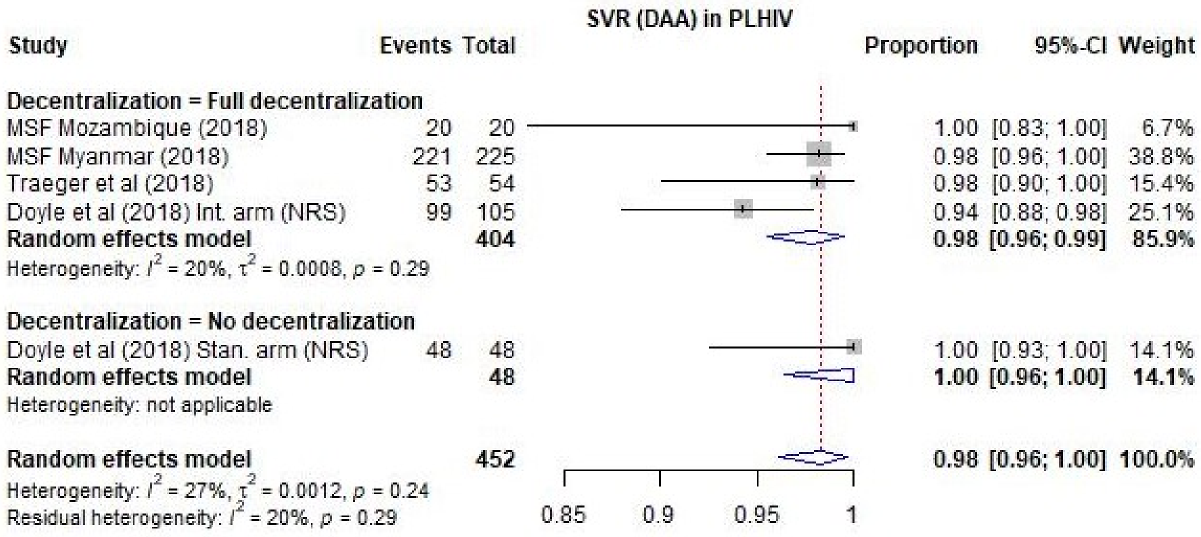

D

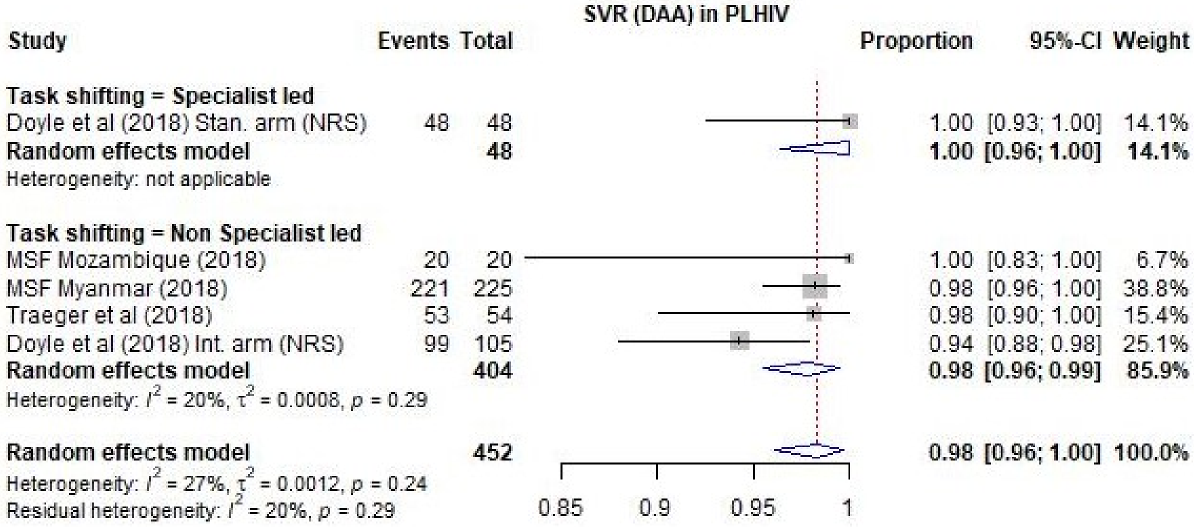
SVR (DAA) in PLHIV
Study Events Total Proportion 95%-CI Weight
Task shifting = Specialist led
Doyle et al (2018) Stan. arm (NRS) 48 48 1.00 [0.93; 1.00] 14.1%
Random effects model 48 1.00 [0.96; 1.00] 14.1%
Heterogeneity: not applicable
Task shifting = Non Specialist led
MSF Mozambique (2018) 20 20 1.00 [0.83; 1.00] 6.7%
MSF Myanmar (2018) 221 225 0.98 [0.96; 1.00] 38.8%
Traeger et al (2018) 53 54 0.98 [0.90; 1.00] 15.4%
Doyle et al (2018) Int. arm (NRS) 99 105 0.94 [0.88; 0.98] 25.1%
Random effects model 404 0.98 [0.96; 0.99] 85.9%
Heterogeneity: I^2 = 20%, τ^2 = 0.0008, p = 0.29
Random effects model 452 0.98 [0.96; 1.00] 100.0%
Heterogeneity: I^2 = 27%, τ^2 = 0.0012, p = 0.24
Residual heterogeneity: I^2 = 20%, p = 0.29
0.85
0.9
0.95
1

Supplementary figures 4 A – C. Impact of task-shifting on SVR 12 for DAAs for (A) PWID, (B) the general population, and (C) people in prisons.

A.

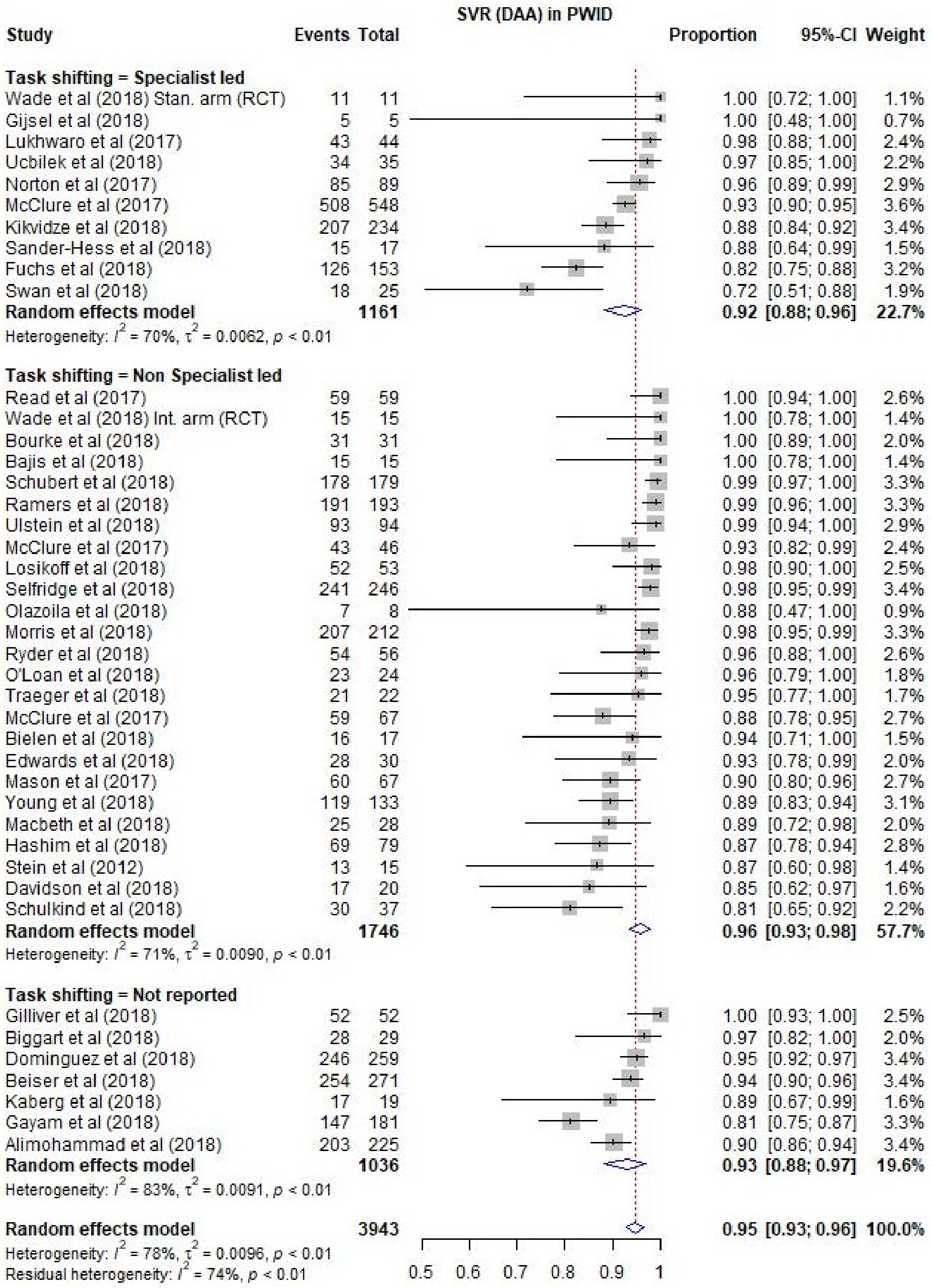

B.

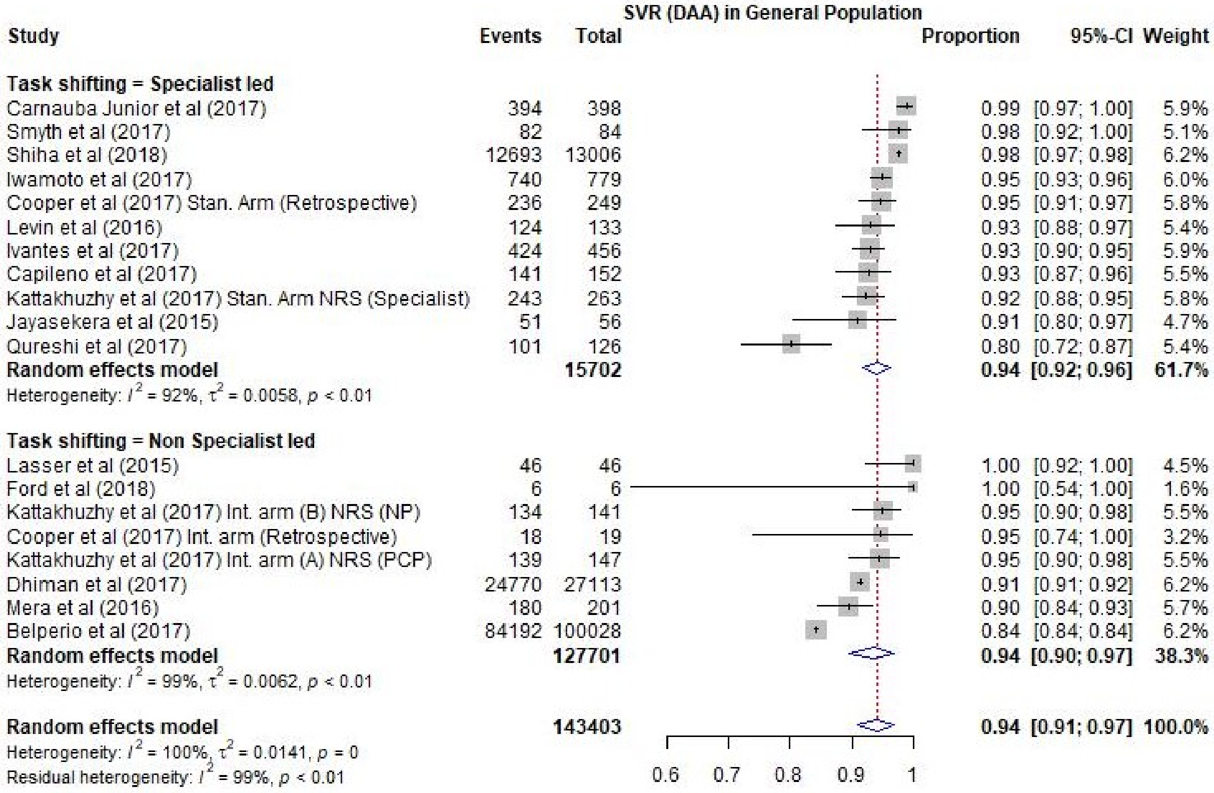
SVR (DAA) in General Population
Study
Events
Total
Proportion
95%-CI
Weight
Task shifting = Specialist led
Carnauba Junior et al (2017) 394 398 0.99 [0.97; 1.00] 5.9%
Smyth et al (2017) 82 84 0.98 [0.92; 1.00] 5.1%
Shiha et al (2018) 12693 13006 0.98 [0.97; 0.98] 6.2%
Iwamoto et al (2017) 740 779 0.95 [0.93; 0.96] 6.0%
Cooper et al (2017) Stan. Arm (Retrospective) 236 249 0.95 [0.91; 0.97] 5.8%
Levin et al (2016) 124 133 0.93 [0.88; 0.97] 5.4%
Ivantes et al (2017) 424 456 0.93 [0.90; 0.95] 5.9%
Capileno et al (2017) 141 152 0.93 [0.87; 0.96] 5.5%
Kattakhuzhy et al (2017) Stan. Arm NRS (Specialist) 243 263 0.92 [0.88; 0.95] 5.8%
Jayasekera et al (2015) 51 56 0.91 [0.80; 0.97] 4.7%
Qureshi et al (2017) 101 126 0.80 [0.72; 0.87] 5.4%
Random effects model 15702 0.94 [0.92; 0.96] 61.7%
Heterogeneity: I^2 = 92%, τ^2 = 0.0058, p < 0.01
Task shifting = Non Specialist led
Lasser et al (2015) 46 46 1.00 [0.92; 1.00] 4.5%
Ford et al (2018) 6 6 1.00 [0.54; 1.00] 1.6%
Kattakhuzhy et al (2017) Int. arm (B) NRS (NP) 134 141 0.95 [0.90; 0.98] 5.5%
Cooper et al (2017) Int. arm (Retrospective) 18 19 0.95 [0.74; 1.00] 3.2%
Kattakhuzhy et al (2017) Int. arm (A) NRS (PCP) 139 147 0.95 [0.90; 0.98] 5.5%
Dhiman et al (2017) 24770 27113 0.91 [0.91; 0.92] 6.2%
Mera et al (2016) 180 201 0.90 [0.84; 0.93] 5.7%
Belperio et al (2017) 84192 100028 0.84 [0.84; 0.84] 6.2%
Random effects model 127701 0.94 [0.90; 0.97] 38.3%
Heterogeneity: I^2 = 99%, τ^2 = 0.0062, p < 0.01
Random effects model 143403 0.94 [0.91; 0.97] 100.0%
Heterogeneity: I^2 = 100%, τ^2 = 0.0141, p = 0
Residual heterogeneity: I^2 = 99%, p < 0.01
0.6
0.7
0.8
0.9
1

C.

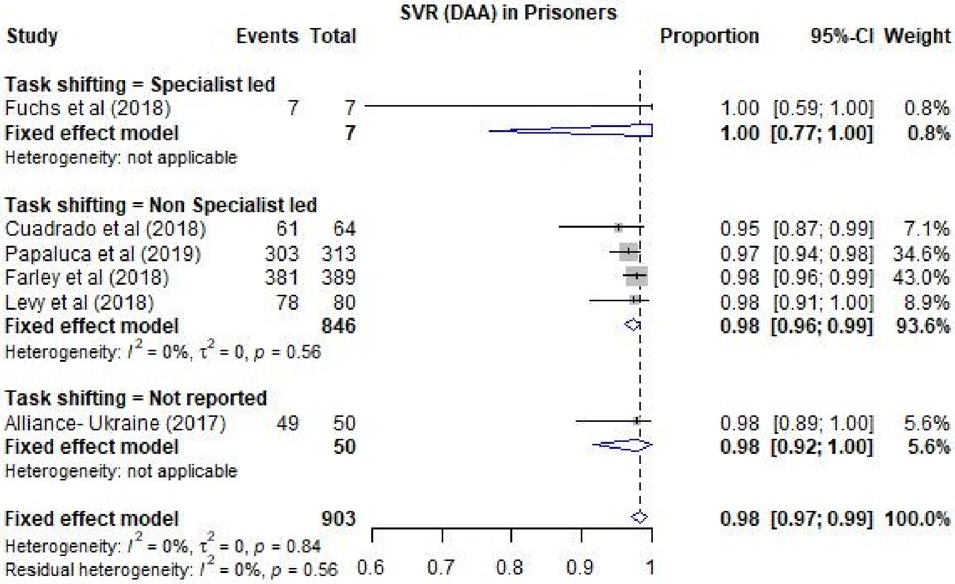
SVR (DAA) in Prisoners
Study
Events
Total
Proportion
95%-CI
Weight
Task shifting = Specialist led
Fuchs et al (2018) 7 7 1.00 [0.59; 1.00] 0.8%
Fixed effect model 7 1.00 [0.77; 1.00] 0.8%
Heterogeneity: not applicable
Task shifting = Non Specialist led
Cuadrado et al (2018) 61 64 0.95 [0.87; 0.99] 7.1%
Papaluca et al (2019) 303 313 0.97 [0.94; 0.98] 34.6%
Farley et al (2018) 381 389 0.98 [0.96; 0.99] 43.0%
Levy et al (2018) 78 80 0.98 [0.91; 1.00] 8.9%
Fixed effect model 846 0.98 [0.96; 0.99] 93.6%
Heterogeneity: I^2 = 0%, τ^2 = 0, p = 0.56
Task shifting = Not reported
Alliance- Ukraine (2017) 49 50 0.98 [0.89; 1.00] 5.6%
Fixed effect model 50 0.98 [0.92; 1.00] 5.6%
Heterogeneity: not applicable
Fixed effect model 903 0.98 [0.97; 0.99] 100.0%
Heterogeneity: I^2 = 0%, τ^2 = 0, p = 0.84
Residual heterogeneity: I^2 = 0%, p = 0.56
0.6
0.7
0.8
0.9
1

Supplementary figures 5 A – D: Impact of task-shifting on SVR 12 for IFN for (A) PWID, (B) the general population, (C) people in prisons, and (D) PLHIV.

A.

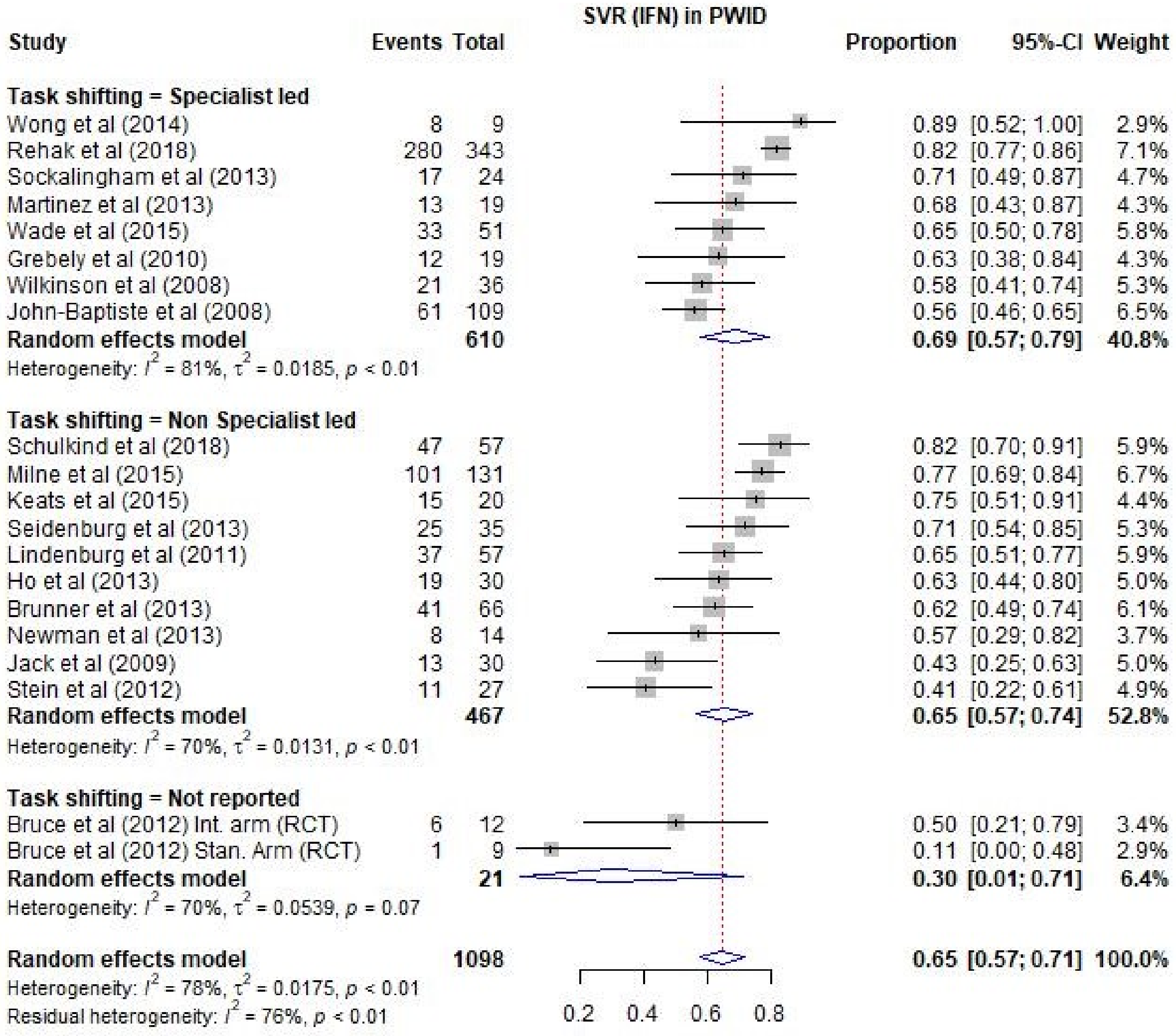

B.

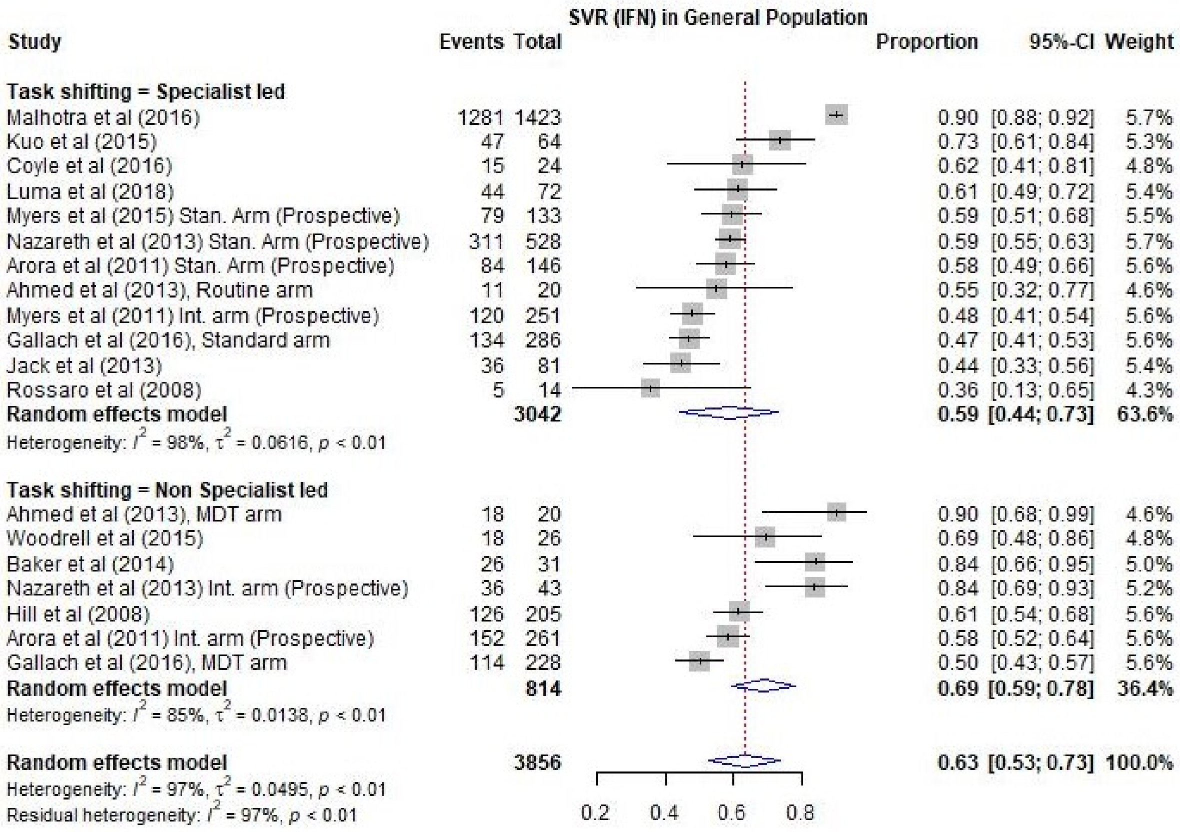
SVR (IFN) in General Population
Study Events Total Proportion 95%-CI Weight
Task shifting = Specialist led
Malhotra et al (2016) 1281 1423 0.90 [0.88; 0.92] 5.7%
Kuo et al (2015) 47 64 0.73 [0.61; 0.84] 5.3%
Coyle et al (2016) 15 24 0.62 [0.41; 0.81] 4.8%
Luma et al (2018) 44 72 0.61 [0.49; 0.72] 5.4%
Myers et al (2015) Stan. Arm (Prospective) 79 133 0.59 [0.51; 0.68] 5.5%
Nazareth et al (2013) Stan. Arm (Prospective) 311 528 0.59 [0.55; 0.63] 5.7%
Arora et al (2011) Stan. Arm (Prospective) 84 146 0.58 [0.49; 0.66] 5.6%
Ahmed et al (2013), Routine arm 11 20 0.55 [0.32; 0.77] 4.6%
Myers et al (2011) Int. arm (Prospective) 120 251 0.48 [0.41; 0.54] 5.6%
Gallach et al (2016), Standard arm 134 286 0.47 [0.41; 0.53] 5.6%
Jack et al (2013) 36 81 0.44 [0.33; 0.56] 5.4%
Rossaro et al (2008) 5 14 0.36 [0.13; 0.65] 4.3%
Random effects model 3042 0.59 [0.44; 0.73] 63.6%
Heterogeneity: I^2 = 98%, τ^2 = 0.0616, p < 0.01
Task shifting = Non Specialist led
Ahmed et al (2013), MDT arm 18 20 0.90 [0.68; 0.99] 4.6%
Woodrell et al (2015) 18 26 0.69 [0.48; 0.86] 4.8%
Baker et al (2014) 26 31 0.84 [0.66; 0.95] 5.0%
Nazareth et al (2013) Int. arm (Prospective) 36 43 0.84 [0.69; 0.93] 5.2%
Hill et al (2008) 126 205 0.61 [0.54; 0.68] 5.6%
Arora et al (2011) Int. arm (Prospective) 152 261 0.58 [0.52; 0.64] 5.6%
Gallach et al (2016), MDT arm 114 228 0.50 [0.43; 0.57] 5.6%
Random effects model 814 0.69 [0.59; 0.78] 36.4%
Heterogeneity: I^2 = 85%, τ^2 = 0.0138, p < 0.01
Random effects model 3856 0.63 [0.53; 0.73] 100.0%
Heterogeneity: I^2 = 97%, τ^2 = 0.0495, p < 0.01
Residual heterogeneity: I^2 = 97%, p < 0.01
0.2 0.4 0.6 0.8

C.

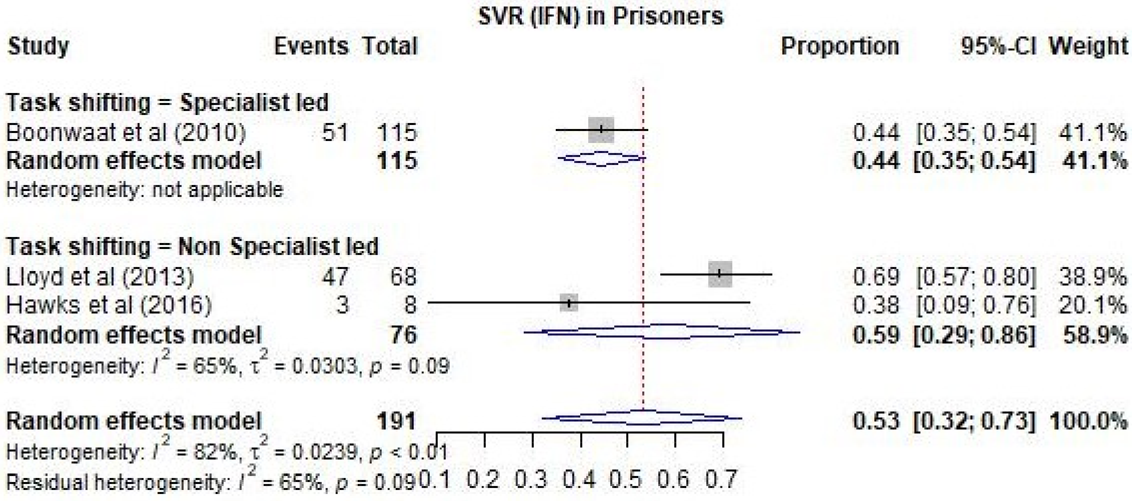
SVR (IFN) in Prisoners
Study Events Total Proportion 95%-CI Weight
Task shifting = Specialist led
Boonwaat et al (2010) 51 115 0.44 [0.35; 0.54] 41.1%
Random effects model 115 0.44 [0.35; 0.54] 41.1%
Heterogeneity: not applicable
Task shifting = Non Specialist led
Lloyd et al (2013) 47 68 0.69 [0.57; 0.80] 38.9%
Hawks et al (2016) 3 8 0.38 [0.09; 0.76] 20.1%
Random effects model 76 0.59 [0.29; 0.86] 58.9%
Heterogeneity: I^2 = 65%, τ^2 = 0.0303, p = 0.09
Random effects model 191 0.53 [0.32; 0.73] 100.0%
Heterogeneity: I^2 = 82%, τ^2 = 0.0239, p < 0.01
Residual heterogeneity: I^2 = 65%, p = 0.09
0.1 0.2 0.3 0.4 0.5 0.6 0.7

D.

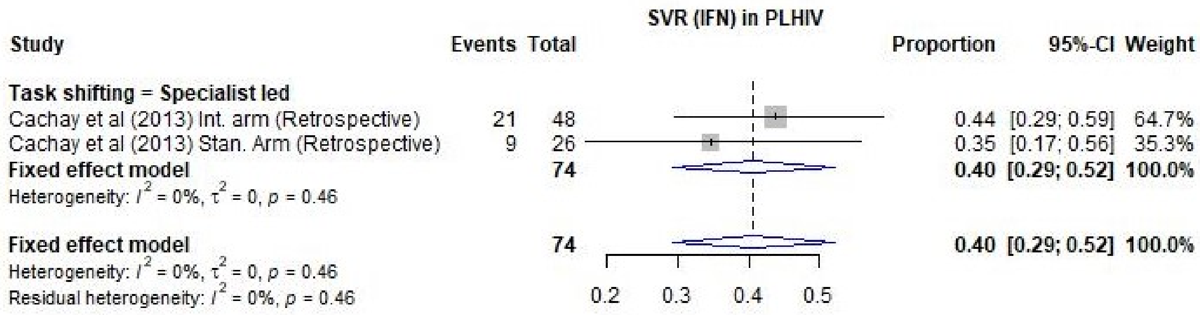
SVR (IFN) in PLHIV
Study
Events Total
Proportion
95%-CI Weight
Task shifting = Specialist led
Cachay et al (2013) Int. arm (Retrospective) 21 48 0.44 [0.29; 0.59] 64.7%
Cachay et al (2013) Stan. Arm (Retrospective) 9 26 0.35 [0.17; 0.56] 35.3%
Fixed effect model 74 0.40 [0.29; 0.52] 100.0%
Heterogeneity: I^2 = 0%, τ^2 = 0, p = 0.46
Fixed effect model 74 0.40 [0.29; 0.52] 100.0%
Heterogeneity: I^2 = 0%, τ^2 = 0, p = 0.46
Residual heterogeneity: I^2 = 0%, p = 0.46
0.2 0.3 0.4 0.5

Supplementary table 4: Meta-analysis estimates (with 95% confidence intervals) stratified by setting (NSP, OST, other) for studies among PWID, for each decentralization type group.

Setting	Full decentralization (FD)	Partial decentralization (PD)	No decentralization (ND)	P-value FD vs PD	P-value FD vs ND	P-value PD vs ND
			**Serological testing uptake**			
**NSP**	45% (40-51) [n=1]	29% (27-30) [n=2]	[n=0]	0.979	NA	NA
**OST**	88% (77-96) [n=5]	100% (87-100) [n=1]	[n=0]	0.260	NA	NA
**Other**	[n=0]	[n=0]	[n=0]	NA	NA	NA
			**NAT uptake**			
**NSP**	87% (83-91) [n=2]	73% (37-97) [n=3]	45% (40-51) [n=1]	0.621	0.329	0.483
**OST**	99% (98-100) [n=7]	87% (81-92) [n=8]	[n=0]	0.001	NA	NA
**Other**	99% (96-100) [n=1]	77% (41-98) [n=3]	100% (94-100) [n=1]	0.422	0.895	0.361
			**Linkage Rate**			
**NSP**	62% (43-79) [n=3]	54% (8-96) [n=4]	[n=0]	0.842	NA	NA
**OST**	75% (53-92) [n=8]	53% (41-64) [n=10]	[n=0]	0.085	NA	NA
**Other**	72% (62-83) [n=3]	51% (45-57) [n=2]	52% (41-62) [n=2]	0.423	0.361	0.910
			**Treatment Uptake**			
**NSP**	65% (39-87) [n=5]	74% (69-78) [n=1]	[n=0]	0.777	NA	NA
**OST**	62% (49-74) [n=22]	61% (43-77) [n=8]	44% (14-79) [n=1]	0.949	0.634	0.660
**Other**	73% (60-84) [n=8]	62% (40-81) [n=5]	35% (23-48) [n=3]	0.350	0.031	0.154
			**Cure Assessment**			
**NSP**	94% (83-100) [n=9]	[n=0]	[n=0]	NA	NA	NA
**OST**	89% (82-94) [n=28]	87% (70-97) [n=6]	[n=0]	0.810	NA	NA
**Other**	79% (59-94) [n=8]	71% (54-85) [n=1]	95% (63-100) [n=3]	0.794	0.421	0.470
			**SVR12 IFN**			
**NSP**	79% (70-88) [n=2]	[n=0]	[n=0]	NA	NA	NA
**OST**	64% (55-71) [n=14]	76% (57-91) [n=2]	11% (0-48) [n=1]	0.343	0.024	0.017
**Other**	63% (44-80) [n=1]	[n=0]	[n=0]	NA	NA	NA
			**SVR12 DAA**			
**NSP**	93% (84-99) [n=5]	88% (84-92) [n=1]	[n=0]	0.692	NA	NA
**OST**	96% (93-99) [n=17]	96% (94-98) [n=3]	[n=0]	0.949	NA	NA
**Other**	95% (92-97) [n=9]	85% (72-94) [n=3]	94% (91-97) [n=4]	0.097	0.860	0.120

NSP: Needle and syringe program; OST: Opiate Substitution Therapy; IFN: Interferon; DAA: Direct acting antivirals; SVR12: Sustained virologic response after 12 weeks. P-values relate to double arcsin transformed meta-regression analyses across levels of decentralization.

Supplementary table 5: Meta-regression univariable coefficient estimates across the stages of the cascade of care*

Subgroup	Screening uptake	NAT uptake	Linkage to care	Treatment uptake	Cure assessment	SVR12
**Population**						
PWID vs. General population	0.45 (-0.04, 0.94)	-0.02 (-0.24, 0.19)	**-0.19 (-0.34, -0.05)**	0.01 (-0.11, 0.12)	0.02 (-0.11, 0.16)	**0.08 (0.02, 0.15)**
Prisoners vs. General population	-0.02 (-0.54, 0.51)	0.05 (-0.23, 0.32)	-0.05 (-0.24, 0.15)	0.01 (-0.18, 0.19)	-0.05 (-0.35, 0.25)	0.10 (-0.01, 0.22)
PLHIV vs. General population	**0.83 (0.01, 1.65)**	-0.02 (-0.42, 0.38)	--	-0.18 (-0.41, 0.06)	0.22 (-0.08, 0.51)	**0.12 (0.00, 0.25)**
**Population restricted to IFN**						
PWID vs. General population	--	--	--	-0.25 (-0.53, 0.04)	0.09 (-0.12, 0.31)	0.00 (-0.13, 0.14)
Prisoners vs. General population	--	--	--	-0.23 (-0.69, 0.23)	-0.19 (-0.55, 0.18)	-0.11 (-0.39, 0.16)
PLHIV vs. General population	--	--	--	-0.49 (-1.04, 0.07)	0.29 (-0.14, 0.72)	-0.24 (-0.57, 0.08)
**Population restricted to DAAs**						
PWID vs. General population	--	--	--	0.12 (-0.03, 0.27)	0 (-0.18, 0.19)	0.09 (-0.03, 0.21)
Prisoners vs. General population	--	--	--	0.16 (-0.07, 0.39)	0.09 (-0.38, 0.56)	0.01 (-0.06, 0.08)
PLHIV vs. General population	--	--	--	0.05 (-0.23, 0.34)	0.18 (-0.22, 0.57)	**0.13 (0.00, 0.26)**
**Decentralization**						
Partial vs. No decentralization	-0.29 (-1.19, 0.61)	0.10 (-0.27, 0.46)	-0.14 (-0.53, 0.24)	0.09 (-0.09, 0.26)	-0.02 (-0.23, 0.19)	0.08 (-0.02, 0.17)
Full vs. No decentralization	0.07 (-0.84, 0.97)	0.19 (-0.19, 0.56)	0.08 (-0.31, 0.46)	0.12 (-0.03, 0.27)	0.00 (-0.17, 0.16)	**0.08 (0.00, 0.16)**
**Decentralization restricted to IFN**						
Partial vs. No decentralization	--	--	--	-0.23 (-0.69, 0.23)	-0.19 (-0.55, 0.18)	-0.11 (-0.39, 0.16)
Full vs. No decentralization	--	--	--	-0.49 (-1.04, 0.07)	0.29 (-0.14, 0.72)	-0.24 (-0.57, 0.08)
**Decentralization restricted to DAAs**						
Partial vs. No decentralization	--	--	--	0.16 (-0.07, 0.39)	0.09 (-0.38, 0.56)	0.09 (-0.03, 0.21)
Full vs. No decentralization	--	--	--	0.05 (-0.23, 0.34)	0.18 (-0.22, 0.57)	**0.13 (0.00, 0.26)**
**Task shifting**						
Non-specialist vs specialist	--	--	--	--	--	**0.13 (0.07, 0.19)**
Non-specialist vs specialist (IFN only)	--	--	--	--	--	0.07 (-0.06, 0.20)
Non-specialist vs specialist (DAAs only)	--	--	--	--	--	0.04 (-0.02, 0.10)
**Low- and middle-income countries**						
LMIC vs high income	0.46 (-0.15, 1.06)	-0.06 (-0.41, 0.30)	0.23 (-0.30, 0.76)	0.11 (-0.14, 0.36)	-0.09 (-0.30, 0.13)	0.09 (-0.01, 0.19)

SVR12: Sustained virologic response after 12 weeks; IFN: Interferon; DAA: Direct acting antivirals; PWID: People who inject drugs; PLHIV: People living with HIV; NAT: Nucleic Acid Testing *Each outcome measure was double arcsin-transformed.

Supplementary table 5 shows, relative to the general population, studies among PLHIV had higher uptake of testing, those among PWID had lower linkage to care, and both had higher SVR12 rates. Similarly, the strong evidence of improved SVR12 using full decentralization relative to no decentralization was driven by DAA studies. In contrast, the evidence of improved SVR12 using task-shifting to non-specialist was only observed when DAA and IFN studies were combined.

Supplementary table 6: Associations (coefficients with 95% confidence intervals [CIs]) between decentralization level and the outcome measures for each population group produced by the meta-regression analyses.

	Coefficients (95% CIs) from meta-regression			
	**PWID**	**Gen-pop**	**Prisoners**	**PLHIV**
**Screening uptake**				
No decentralization	NA	Comparator	NA	NA
Partial decentralization	Comparator	-0.78 (-3.29, 1.73)	Comparator	NA
Full decentralization	0.30 (-0.81, 1.41)	-1.05 (-4.81, 2.71)	0.34 (-0.78, 1.46)	NA
**NAT uptake**				
No decentralization	Comparator	Comparator	NA	NA
Partial decentralization	0.00 (-0.78, 0.79)	0.49 (-0.17, 1.16)	Comparator	NA
Full decentralization	0.60 (-0.20, 1.41)	-0.22 (-1.09, 0.66)	-0.14 (-0.65, 0.38)	NA
**Linkage rate**				
No decentralization	Comparator	Comparator	NA	NA
Partial decentralization	0.12 (-0.79, 1.02)	0.13 (-0.79, 1.05)	Comparator	NA
Full decentralization	0.51 (-0.40, 1.42)	-0.15 (-1.06, 0.77)	1.06 (0.32, 1.80)	NA
**Treatment uptake (IFN)**				
No decentralization	Comparator	Comparator	NA	NA
Partial decentralization	-0.21 (-1.76, 1.34)	-0.65 (-2.22, 0.91)	NA	NA
Full decentralization	-0.05 (-1.42, 1.32)	0.42 (-0.72, 1.55)	NA	NA
**Treatment uptake (DAA)**				
No decentralization	Comparator	Comparator	NA	NA
Partial decentralization	0.56 (-0.07, 1.19)	0.33 (-0.90, 1.57)	Comparator	NA
Full decentralization	0.69 (0.09, 1.29)	-0.18 (-1.41, 1.06)	0.76 (-1.53, 3.04)	NA
**Cure assessment (IFN)**				
No decentralization	NA	Comparator	NA	NA
Partial decentralization	Comparator	0.49 (-0.62, 1.60)	NA	NA
Full decentralization	0.60 (-0.53, 1.74)	0.24 (-0.54, 1.03)	NA	NA
**Cure assessment (DAA)**				
No decentralization	Comparator	Comparator	NA	NA
Partial decentralization	-0.15 (-0.99, 0.69)	0.10 (-1.01, 1.21)	NA	NA
Full decentralization	-0.27 (-0.99, 0.44)	0.06 (-0.96, 1.08)	NA	NA
**SVR12 (IFN)**				
No decentralization	Comparator	Comparator	NA	NA
Partial decentralization	1.32 (0.31, 2.32)	-0.15 (-0.80, 0.51)	NA	NA
Full decentralization	1.09 (0.23, 1.95)	-0.13 (-0.64, 0.37)	NA	NA
**SVR12 (DAA)**				
No decentralization	Comparator	Comparator	NA	1
Partial decentralization	-0.14 (-0.48, 0.21)	-0.11 (-0.39, 0.16)	Comparator	NA
Full decentralization	0.03 (-0.27, 0.33)	-0.17 (-0.42, 0.08)	0.02 (-0.97, 1.01)	-0.20 (-0.73, 0.32)

Supplementary table 7: Associations between task-shifting level and the outcome measures for each population group produced by the meta-regression analyses.

	Coefficients (95% CIs) from meta-regression			
	**PWID**	**Gen-pop**	**Prisoners**	**PLHIV**
**SVR12 (IFN)**				
Specialist led	Comparator	Comparator	Comparator	NA
Non-specialist led	-0.08 (-0.41, 0.25)	0.25 (-0.25, 0.76)	0.27 (-5.69, 6.24)	NA
**SVR12 (DAA)**				
Specialist led	Comparator	Comparator	Comparator	Comparator
Non-specialist led	0.17 (-0.02, 0.36)	-0.07 (-0.26, 0.13)	0.02 (-1.11, 1.15)	-0.20 (-0.73, 0.32)

Supplementary table 8. Comparison of outcomes across the cascade of care (estimate and 95% confidence intervals) for studies with a comparator arm and non-comparator studies that used either IFN or DAA based treatment, according to population group and:- (a) level of decentralization for outcomes (linkage to care, treatment uptake, cure assessment, and SVR12) and (b) task-shifting to non-specialists for SVR12.

(a) Decentralisation level (full, partial, and none)

			Comparator studies				Non-comparator studies			
			Levels of Decentralization				Levels of Decentralization			
**Population**	**Design**	**(a) Study***	**Full**	**Partial**	**None**	**P-value**	**Full**	**Partial**	**None**	**P-value**
			**LINKAGE TO CARE**							
PWID	RCT	Wade	88% (77-94)		67% (54-78)		n=12		n=1	
**Overall PWID Full vs None**			**88% (77-94)**		**67% (54-78)**	**0.008**	**73% (56-87)**		**26% (13-44)**	**<0.001**
PWID	RCT	Radley	39% (35-44)	26% (22-30)			n=12	n=15		
**Overall PWID Full vs Partial**			**39% (35-44)**	**26% (22-30)**		**<0.001**	**73% (56-87)**	**55% (39-70)**		**0.108**
			**TREATMENT UPTAKE**							
PWID	RCT	Wade [DAA]	74% (60-85)		39% (26-53)					
PWID	RCS	Middleton [DAA]	98% (88-100)		27% (17-41)		n=21		n=1	
**Overall PWID [DAA] Full vs None**			**88% (65-100)**		**33% (25-43)**	**<0.001**	**71% (62-79)**		**56% (21-86)**	**0.381**
PWID	PCS	Alavi [DAA]	88% (77-95)	100% (91-100)						
PWID	RCT	Radley [DAA]	52% (45-59)	44% (36-53)			n=21	n=10		
**Overall PWID [DAA] Full vs Partial**			**72% (32-98)**	**83% (10-100)**		**0.954**	**71% (62-79)**	**64% (53-74)**		**0.310**
Gen-pop	RCS	Cooper [DAA]	63% (47-77)	52% (48-57)			n=4	n=4		
**Overall Gen-pop [DAA] Full vs Partial**			**63% (47-77)**	**52% (48-57)**		**0.146**	**40% (16-67)**	**74% (63-84)**		**0.016**
PWID	RCT	Bruce [IFN]	83% (55-95)		44% (19-73)		n=9		n=0	
**Overall PWID [IFN] Full vs None**			**83% (55-95)**		**44% (19-73)**	**0.111~~023~~**	**39% (23-56)**		**NA**	**NA**
PLHIV	RCS	Cachay [IFN]	25% (19-31)		16% (11-22)		n=0		n=0	

**Overall PLHIV [IFN] Full vs None**			**25% (19-31)**		**16% (11-22)**	**0.04330**	**NA**		**NA**	**NA**
Gen-pop	PCS	Myers [IFN]	100% (100-100)		99% (96-100)		n=4		n=6	
**Overall Gen-pop [IFN] Full vs None**			**100% (100-100)**		**99% (96-100)**	**0.959**	**73% (34-98)**		**53% (28-77)**	**0.331**
**CURE ASSESSMENT**										
PWID	RCT	Wade [DAA]	47% (31-64)		69% (44-86)		n=28		n=2	
**Overall PWID [DAA] Full vs None**			**47% (31-64)**		**69% (44-86)**	**0.130**	**83% (76-90)**		**100% (98-100)**	**<0.001**
PWID	RCT	Radley [DAA]	96% (91-99)	95% (87-98)			n=28	n=4		
**Overall PWID [DAA] Full vs Partial**			**96% (91-99)**	**95% (87-98)**		**0.697**	**83% (76-90)**	**85% (73-94)**		**0.756**
Gen-pop	RCS	Cooper [DAA]	78% (73-82)	70% (50-86)			n=10	n=4		
**Overall Gen-pop [DAA] Full vs Partial**			**78% (73-82)**	**70% (50-86)**		**0.826**	**86% (79-92)**	**89% (47-100)**		**0.829**
Gen-pop	PCS	Arora [IFN]	100% (99-100)	100% (97-100)			n=5	n=1		
**Overall Gen-pop [IFN] Full vs Partial**			**100% (99-100)**	**100% (97-100)**		**1.000**	**91% (64-100)**	**93% (92-94)**		**0.828**
PLHIV	RCS	Cachay [IFN]	100% (87-100)		100% (93-100)		n=0		n=0	
**Overall PLHIV [IFN] Full vs None**			**100% (87-100)**		**100% (93-100)**	**1.000**	**NA**		**NA**	**NA**
**SVR12**										
Gen-pop	PCS	Arora [IFN]	58% (52-64)	58% (49-65)						
Gen-pop	PCS	Nazareth [IFN]	84% (69-93)	59% (55-68)			n=4	n=2		
**Overall Gen-pop [IFN] Full vs Partial**			**62% (57-68)**	**59% (55-62)**		**0.32567**	**58% (41-74)**	**66% (52-79)**		**0.462**
PWID	RCT	Wade [DAA]	100% (78-100)		100% (72-100)		n=28		n=3	
**Overall PWID [DAA] Full vs None**			**100% (78-100)**		**100% (72-100)**	**0.688**	**95% (93-97)**		**94% (90-97)**	**0.627795**
PWID	PCS	McClure [DAA]	88% (78-95) Nurse<br>93% (82-99) GP	93% (90-95)			n=28	n=6		

**Overall PWID [DAA] Full vs Partial**			**91% (84-95)**	**93% (90-95)**		**NA**	**95% (93-97)**	**90% (83-96)**		**0.149**
PLHIV	NRS	Doyle [DAA]	94% (88-99)		100% (93-100)		n=3		n=0	
**Overall PLHIV [DAA] Full vs None**			**94% (88-99)**		**100% (93-100)**	**NA**	**99% (97-100)**		**NA**	**NA**
Gen-pop	RCS	Cooper [DAA]	95% (74-100)	95% (91-97)			n=10	n=4		
**Overall Gen-pop [DAA] Full vs Partial**			**95% (74-100)**	**95% (91-97)**		**NA**	**93% (90-96)**	**93% (86-98)**		**1.000~~0.312~~**
PWID	RCT	Bruce [IFN]	50% (25-75)		11% (2-44)		n=16		n=0	
**Overall PWID [IFN] Full vs None**			**50% (25-75)**		**11% (2-44)**	**0.127~~019~~**	**66% (59-73)**		**NA**	**NA**
PLHIV	RCS	Cachay [IFN]	44% (31-58)		35% (19-54)		n=0		n=0	
**Overall PLHIV [IFN] Full vs None**			**44% (31-58)**		**35% (19-54)**	**0.458~~25~~**	**NA**		**NA**	**NA**
Gen-pop	PCS	Myers [IFN]	47~~8~~% (42~~1~~-54)		59% (51-68~~7~~)		n=4		n=7	
**Overall Gen-pop [IFN] Full vs None**			**47% (42~~1~~-54~~3~~)**		**59% (51-68)~~38% (14-~~**	**0.026~~501~~**	**58% (41-74)**		**68% (46-87)**	**0.456**

(b) Comparator studies on task-shifting

			Comparator studies			Non-comparator studies		
			Deliverer of care			Deliverer of care		
**Population**	**Type**	**(b) Study***	**Non-specialist**	**Specialist**	**P-value**	**Non-specialist**	**Specialist**	**P-value**
		**SVR12**						
PWID	RCT	Wade [DAA]	PCPs/nurses 100% (76-100)	100% (74-100)				
PWID	PCS	McClure [DAA]	Nurses 88% (78-95), GP 93% (82-99)	93% (90-95)		n=22	n=8	
**Overall PWID [DAA] Task-shifting**			**93% (86-98)**	**95% (92-97)**	**0.460**	**96% (94-98)**	**92% (86-96)**	**0.145**
PLHIV	NRS	Doyle [DAA]	Nurses 94% (88-98)	100% (93-100)		n=3	n=0	
**Overall PLHIV [DAA] Task-shifting**			**94% (88-98)**	**100% (93-100)**	**0.065**	**99% (97-100)**	**NA**	**NA**
Gen-pop	RCS	Cooper [DAA]	Nurses 95% (74-100)	95% (91-97)				
Gen-pop	NRS	Kattakuzhy [DAA]	PCP 95% (90-98), nurses 95% (90-98)	92% (89-95)		n=5	n=9	
**Overall Gen-pop [DAA] Task-shifting**			**95% (92-98)**	**94% (91-96)**	**0.466**	**93% (87-97)**	**94% (91-97)**	**0.737**
Gen-pop	RCS	Ahmed [IFN]	Multidisciplinary team 90% (68-99)	55% (32-77)				
Gen-pop	PCS	Arora [IFN]	PCP 58% (52-64)	58% (49-65)				
Gen-pop	PCS	Gallach [IFN]	Multidisciplinary team 50% (44-56)	47% (41-53)				
Gen-pop	PCS	Nazareth [IFN]	Nurses 84% (69-93)	59% (55-68)		n=3	n=8	
**Overall Gen-pop [IFN] Task-shifting**			**69% (54-82)**	**55% (47-62)**	**0.084**	**71% (56-83)**	**61% (40-80)**	**0.417**

DAA: Direct acting antivirals; PCS: Prospective comparative study; RCT: Randomised controlled trial; RCS: Retrospective comparative study; NRS: non-randomised study; IFN: Interferon; Gen-pop: General population; PWID: People who inject drugs; PLHIV: People living with HIV; SVR12: Sustained virologic response after 12 week. PCP: Primary Care physician

P-values are from meta-regression analyses across the decentralization types.

Of the total 142 studies, there were a subset of 13 studies (9%) with comparator arms (either randomized trials, non-randomised studies, or prospective cohort studies), that had examined the impact of decentralization of care or task-shifting of care and treatment to non-specialists (table 4: DAA regimens; supplementary table 7: IFN). Table 4 shows a comparison of available outcomes from this subgroup of studies using DAA regimens (those using IFN are also shown in Supplementary table 7) across arms with different levels of decentralization and task-shifting with the corresponding results for the non-comparator studies (NCS). There were generally few comparator studies (CS) (7 for decentralisation and 5 for task-shifting analyses) compared to non-comparator ones, and the majority of these were among PWID. Findings were generally consistent between comparator and non-comparator studies. Among PWID, the increase in linkage to care was greatest for FD compared to ND for linkage to care, and treatment uptake, with smaller increases observed with FD compared PD for linkage to care and treatment uptake. Uptake rates for cure assessment were higher in ND compared to FD, and not different between FD and PD for either PWID, or general population. For SVR12, there were no differences across decentralisation levels for any population in either CS or NCS. Similarly, for task-shifting, SVR12 rates were comparable for task-shifting to non-specialists vs. specialists across CS and NCS for all populations.

Meta-analyses of these studies with comparator arms showed similar results for full versus partial or no decentralization for outcomes (linkage to care, treatment uptake, cure assessment, and SVR12) across the care cascade, to that observed from the overall meta-analysis, based on the estimates and confidence intervals, with better outcomes seen for higher levels of decentralization, and similar SVR12 rates for DAAs across levels of decentralization and for specialist and non-specialist led care. Meta-regression analyses showed no strong evidence of differences in outcomes between decentralization or task-shifting groups, but formal tests were not possible for the majority of comparisons due to a lack of comparative studies, with only one study available for many outcome/population group stratifications

Whilst in the overall analysis there was strong evidence of improved DAA treatment uptake among PWID when comparing full versus no decentralization, there was weak evidence in the comparator studies, FD 89% (95%CI 81-95), ND 33% (95%CI 23-42) – p-value=0.090. However, this effect was stronger than in the non-comparator arm studies, FD 71% (95%CI 62-79), ND 56% (95%CI 21-86), indicating the overall effect was being driven by the studies with comparator arms.

References

1. Hashim A, O'Sullivan M, Williams H, Verma S. Developing a community HCV service: project ITTREAT (integrated community-based test - stage - TREAT) service for people who inject drugs. Prim Health Care Res Dev. 2018 Mar;19(2):110-120. doi: 10.1017/S1463423617000731.
2. Schulkind J, Ahmad F, Stephens B, et al. Eradicate Hepatitis C: a pilot treatment as prevention in active drug users. Poster Abstract, The International Liver Congress, April 2018.
3. Wade AJ, Doyle JS, Gane E, et al. Preliminary analysis of the Prime Study: a randomised controlled trial comparing the hepatitis C care cascade in primary vs tertiary care. Poster Abstract, International Liver Conference, Paris, 2018
4. Ramers C, Rojas S, Khasira M, et al. Strategic Elimination: Efficacy of Hepatitis C Treatment in PWID in an urban underserved clinic. Poster Abstract, The International Liver Congress, April 2018.
5. Davidson K, Bathgate A, Budd A, et al. Treatment of Hepatitis C in a dedicated GP practice by Multidisciplinary team. Poster Abstract, International Liver Conference, Paris, 2018
6. Morris L, Smirnov A, Kvassay A, et al. Initial outcomes of integrated community-based hepatitis C treatment for people who inject drugs: findings from the Queensland Injectors health network. Int J Drug Policy. 2017 Sep;47:216-220. doi: 10.1016/j.drugpo.2017.05.056.
7. Mason K, Dodd Z, Guyton M, et al. Understanding real-world adherence in the directly acting antiviral era: A prospective evaluation of adherence among people with a history of drug use at a community-based program in Toronto, Canada. Int J Drug Policy. 2017 Sep;47:202-208. doi: 10.1016/j.drugpo.2017.05.025.
8. Read P, Lothian R, Chronister K, et al. Delivering direct acting antiviral therapy for hepatitis C to highly marginalized and current drug injecting populations in a targeted primary health care setting. International Journal of Drug Policy, Volume 47, 209 – 215. doi: 10.1016/j.drugpo.2017.05.032.
9. Norton BL, Fleming J, Bachhuber MA, et al. High HCV cure rates for people who use drugs treated with direct acting antiviral therapy at an urban primary care clinic. Int J Drug Policy. 2017 Sep;47:196-201. doi: 10.1016/j.drugpo.2017.07.021
10. Keats J, Micallef M, Grebely J, et al. Assessment and delivery of treatment for hepatitis C virus infection in an opioid substitution treatment clinic with integrated peer-based support in Newcastle, Australia. International Journal of Drug Policy, Volume 26, Issue 10, 999 – 1006. Epub 2015 Jul 17. doi: 10.1016/j.drugpo.2015.07.006.
11. Alavi M, Grebely J, Micallef M, et al. Assessment and treatment of hepatitis C virus infection among people who inject drugs in the opioid substitution setting: ETHOS study. Clin Infect Dis. 2013 Aug;57 Suppl 2:S62-9. doi: 10.1093/cid/cit305.
12. Wade AJ, Macdonald D, Doyle J, et al. The Cascade of Care for an Australian Community-Based Hepatitis C Treatment Service. PLOS One. 2015 Nov. doi.org/10.1371/journal.pone.0142770.
13. Milne R, Price M, Wallace B, et al. From principles to practice: Description of a novel equity-based HCV primary care treatment model for PWID. Int J Drug Policy. 2015 Oct;26(10):1020-7. doi: 10.1016/j.drugpo.2015.07.009
14. Brunner N, Senn O, Rosemann T, et al. Hepatitis C treatment for multimorbid patients with substance use disorder in a primary care-based integrated treatment centre: A retrospective analysis. European Journal of Gastroenterology and Hepatology. 25(11): 1300-1307. doi: 10.1097/MEG.0b013e32836140bb
15. Seidenberg, A, Rosemann T, Senn O. Patients receiving opioid maintenance treatment in primary care: successful chronic hepatitis C care in a real-world setting. BMC Infect Dis. 2013; 13: 9. doi: 10.1186/1471-2334-13-9

16. Newman AI, Beckstead S, Beking D, et al. Treatment of chronic hepatitis C infection among current and former injection drug users within a multidisciplinary treatment model at a community health centre. Canadian Journal of Gastroenterology. 2013;27(4):217-223.
17. Bruce RD, Eiserman J, Acosta A, et al. Developing a Modified Directly Observed Therapy Intervention for Hepatitis C Treatment in a Methadone Maintenance Program: Implications for Program Replication. Am J Drug Alcohol Abuse. 2012 May; 38(3): 206–212.
18. Stein MR, Soloway IJ, Jefferson KS, et al. Concurrent Group Treatment for Hepatitis C: Implementation and Outcomes in a Methadone Maintenance Treatment Program. Journal of substance abuse treatment. 2012;43(4):424-432. doi:10.1016/j.jsat.2012.08.007.
19. Lindenburg CE, Lambers FA, Urbanus AT, et al. Hepatitis C testing and treatment among active drug users in Amsterdam: Results from the DUTCH-C project. European Journal of Gastroenterology and Hepatology. 23(1):23-31, Jan 2011. doi: 10.1097/MEG.0b013e328340c451.
20. Litwin AH, Berg K, Xuan L, et al. Rationale and design of a randomized controlled trial of directly observed hepatitis C treatment delivered in methadone clinics. BMC Infect Dis. 2011; 11: 315. doi: 10.1186/1471-2334-11-315
21. Grebely J, Knight E, Genoway KA, et al. Optimizing assessment and treatment for hepatitis C virus infection in illicit drug users: A novel model incorporating multidisciplinary care and peer support. Eur J Gastroenterol Hepatol. 2010 Mar;22(3):270-7.
22. Jack K, Willott S, Manners J, et al. Clinical trial: a primary-care-based model for the delivery of anti-viral treatment to injecting drug users infected with hepatitis C. Aliment Pharmacol Ther. 2009 Jan;29(1):38-45. doi: 10.1111/j.1365-2036.2008.03872.x.
23. John-Baptiste A, Varenbut M, Tingley M, et al. Treatment of Hepatitis C infection for current or former substance abusers in a community setting. Journal of Viral Hepatitis, Volume 16, Issue 8, August 2009, Pages 557-567. doi.org/10.1111/j.1365-2893.2009.01097.x
24. Alavi M, Poustchi H, Merat S, et al. An intervention to improve HCV testing, linkage to care, and treatment among people who use drugs in Tehran, Iran: the ENHANCE study. 7th International Symposium on Hepatitis Care in Substance Users, Cascais Portugal, 19-21 September 2018.
25. Gayam V, Jayanti R, Mazin K, et al. Direct Acting Antivirals in HCV mono-infection compared to HCV & HIV co-infection in Community Care. 7th International Symposium on Hepatitis Care in Substance Users, Cascais Portugal, 19-21 September 2018.
26. Schubert R, Schutz A, Schwanke C, et al. Interim results of an ongoing project to eradicate HCV in people who inject drugs at risk for non-adherence to direct-acting antivirals in Vienna. 7th International Symposium on Hepatitis Care in Substance Users, Cascais Portugal, 19-21 September 2018.
27. Kaberg M, Edgren E, Hammarberg A, Weiland O. Enhancing the hepatitis C (HCV) care cascade for people who inject drugs (PWID) at the Stockholm Needle Exchange Program. 7th International Symposium on Hepatitis Care in Substance Users, Cascais Portugal, 19-21 September 2018.
28. Selfridge M, Milne R, Drost A, et al. Direct-acting therapy for hepatitis C virus infection and reinfection among people attending an inner-city community health centre in Victoria, Canada. 7th International Symposium on Hepatitis Care in Substance Users, Cascais Portugal, 19-21 September 2018.
29. Traeger M, Pedrana A, Draper B, et al. Estimating Progression Through The Cascade Of Care Among Patients With Hepatitis C Infection In Victoria, Australia After The Introduction Of Direct-Acting Antivirals. 7th International Symposium on Hepatitis Care in Substance Users, Cascais Portugal, 19-21 September 2018.
30. Edwards B, Boothman H, Spollen S, et al. The Development of a Collaborative Model to Optimise Hepatitis C (HCV) Antiviral Treatment in Community Drug Services. 7th International Symposium on Hepatitis Care in Substance Users, Cascais Portugal, 19-21 September 2018.

31. Biggart L, Sills L, Motaleb E, et al. Introduction of combined hepatology/addictions advanced fibrosis clinic leads to high attendance rates amongst cohort with a history of failure to engage with service. 7th International Symposium on Hepatitis Care in Substance Users, Cascais Portugal, 19-21 September 2018.
32. Page J, Maher L, Sheaves F, et al. Positively Hep: A multidisciplinary, incentivised Hepatitis C Project for disadvantaged communities. 7th International Symposium on Hepatitis Care in Substance Users, Cascais Portugal, 19-21 September 2018.
33. Macbeth K, Davidson K, Budd J, Bathgate A. Successful Treatment of Hepatitis C in a Dedicated GP Practice for the Homeless using a Multidisciplinary Approach. 7th International Symposium on Hepatitis Care in Substance Users, Cascais Portugal, 19-21 September 2018.
34. Middleton L, Ritchie T. Combined drug recovery and hepatitis C treatment clinic leads to more effective engagement than traditional care model. 7th International Symposium on Hepatitis Care in Substance Users, Cascais Portugal, 19-21 September 2018.
35. Rehak V, Krekulova L, Slesinger P, et al. 20 years of evolution of the comprehensive model of care for PWID to enhance HCV treatment uptake and outcome. 7th International Symposium on Hepatitis Care in Substance Users, Cascais Portugal, 19-21 September 2018.
36. Ryder N, Woodward S, Voght K, Lindsay M. Improving access to Hepatitis C treatment for people currently injecting drugs, a sexual health clinic model of care. 7th International Symposium on Hepatitis Care in Substance Users, Cascais Portugal, 19-21 September 2018.
37. Bourke M. Treatment of hepatitis C in a community based opioid substitution (OST) clinic. 7th International Symposium on Hepatitis Care in Substance Users, Cascais Portugal, 19-21 September 2018.
38. Bajis S, Cooper L, Smith J, et al. Hepatitis C virus (HCV) testing, liver disease assessment and direct-acting antiviral (DAA) treatment uptake and outcomes in a service for the homeless in Sydney: The LiveRLife study. 7th International Symposium on Hepatitis Care in Substance Users, Cascais Portugal, 19-21 September 2018.
39. Pedrana A, Williams B, Howell J, et al. Straight To The Point - Lessons From The Rapid-EC Study: A Point-Of-Care Hepatitis C Testing Pilot In Needle And Syringe Programs Targeted To People Who Inject Drugs in Australia. 7th International Symposium on Hepatitis Care in Substance Users, Cascais Portugal, 19-21 September 2018.
40. Radley A, de Bruin M, Inglis SK, et al. Preliminary Analysis of the SuperDOT-C Study: A Cluster Randomised Controlled Trial of Pharmacy Led Versus Conventional Treatment for HCV Positive Patients Receiving Daily Opioid Substition Therapy Within NHS Scotland. 7th International Symposium on Hepatitis Care in Substance Users, Cascais Portugal, 19-21 September 2018.
41. Gilliver R, Chronister KJ, Lothian R, et al. Daily dosing of direct acting antivirals from a public opioid substitution therapy (OST) program for marginalised clients in Kings Cross, Sydney. 7th International Symposium on Hepatitis Care in Substance Users, Cascais Portugal, 19-21 September 2018.
42. Midgard H, Backe O, Ulstein K, et al. The cascade of HCV care among people who inject drugs in a Norwegian low-threshold setting: Increasing treatment uptake. 7th International Symposium on Hepatitis Care in Substance Users, Cascais Porgual, 19-21 September 2018.
43. Beiser M. Experience and outcomes of a high volume homeless health center-based HCV treatment program in Boston. 7th International Symposium on Hepatitis Care in Substance Users, Cascais Portugal, 19-21 September 2018.
44. Young M, O'Loan J. Liberating Medeco Inala from Hep C. A study of micro-elimination in primary care. 7th International Symposium on Hepatitis Care in Substance Users, Cascais Portugal, 19-21 September 2018.
45. O'Loan J, Young M. The Kombi Clinic: Driving Out Hep C In South East Queensland Disenfranchised Communities. 7th International Symposium on Hepatitis Care in Substance Users, Cascais Portugal, 19-21 September 2018.

46. Von Bibra S, Allardice K, Chan K. Engaging PWID in community based treatment and care using a nurse-led, social and network model. 7th International Symposium on Hepatitis Care in Substance Users, Cascais Portugal, 19-21 September 2018.
47. Olaizola C, Maitre C, Bidart E, et al. Test and treat: 6 years of HCV rapid testing among drug users in the Bayonne area (France). 7th International Symposium on Hepatitis Care in Substance Users, Cascais Portugal, 19-21 September 2018.
48. Taylor LE, Feinberg J, Kim AY, et al. Real World Direct-Acting Antiviral (DAA) Outcomes Among People Who Inject Drugs (PWID) in the United States: Hepatitis C Real Options (HERO). 7th International Symposium on Hepatitis Care in Substance Users, Cascais Portugal, 19-21 September 2018.
49. McClure T, Olenski M, Ward A, et al. Real-world treatment of hepatitis C in the unrestricted direct-acting antiviral era – comparing liver clinic and community outcomes. The AASLD Liver meeting, Washington DC, USA, 2017.
50. Lukhwaro A. Hepatitis C Testing, Treatment & Care For People Who Inject Drugs: Lessons From Kenya. Poster Abstract, World Hepatitis Summit. São Paulo, Brazil. November 1-3 2017.
51. Barnett T, Lundgren K, Milne R, et al. Treating People Where They Are: Micro-elimination in Practice. 7th International Symposium on Hepatitis Care in Substance Users, Cascais Portugal, 19-21 September 2018.
52. Fragomeli V, Weltman M. Addressing viral hepatitis in the opiate substitution setting: An integrated nursing model of care. Journal of Gastroenterology and Hepatology 2015; 30 (Suppl. 2): 6–11. doi: 10.1111/jgh.12864.
53. Sockalingam S, Blank D, Banga CA, et al. A novel program for treating patients with trimorbidity: hepatitis C, serious mental illness, and active substance use. Eur J Gastroenterol Hepatol. 2013 Dec;25(12):1377-84. doi: 10.1097/MEG.0b013e3283624a28.
54. Ho CJ, Preston C, Fredericks K, et al. A unique model for treating chronic hepatitis C in patients with psychiatric disorders, substance abuse, and/or housing instability. J Addict Med. 2013 Sep-Oct;7(5):320-4. doi: 10.1097/ADM.0b013e31829b1a6c.
55. Wilkinson M, Crawford V, Tippet A, et al. Community-based treatment for chronic hepatitis C in drug users: high rates of compliance with therapy despite ongoing drug use. Aliment Pharmacol Therapy 29, 29-37, 2008. doi: 10.1111/j.1365-2036.2008.03834.x.
56. Ulstein K, Midgard H, Backe O, et al. High efficacy of direct-acting antiviral HCV treatment among people with recent injecting drug use: a real-life experience. 7th International Symposium on Hepatitis Care in Substance Users, Cascais Portugal, 19-21 September 2018.
57. Sander-Hess C, Parker M, Maggs J, et al. INTACCT: INTegrAted Community hepatitis C Treatment. 7th International Symposium on Hepatitis Care in Substance Users, Cascais Portugal, 19-21 September 2018.
58. Kikvidze T, Luhmann N, Avril E, et al. Harm reduction-based and peer-supported hepatitis C treatment for people who inject drugs in Georgia, International Journal of Drug Policy 52 (2018) 16–19. doi: 10.1016/j.drugpo.2017.11.014.
59. Magaldi L, Brown N, Coleman C, et al. Outcomes of Hepatitis C testing, linkage to care and treatment in a community based program in high versus low prevalence sites. Poster Abstract, The International Liver Congress, April 2018.
60. Sutton M, Holloway W, Johnson B. Building a successful hepatitis C screening and linkage to care program with substance users. Poster Abstract, World Hepatitis Summit. São Paulo, Brazil. November 1-3 2017.
61. Porter C, Nickel R, Bensley K, et al. HCV services and syringe exchange programs: partnering to improve access to care for people who inject drugs. Poster Abstract, World Hepatitis Summit. São Paulo, Brazil. November 1-3 2017

62. Blackburn NA, Patel RC, Zibbell JE. Improving screening methods for hepatitis C among people who inject drugs: Findings from the HepTLC initiative, 2012-2014. Public Health Reports, 2016 Supplement 2, Volume 131 doi: 10.1177/00333549161310S214
63. Rajkumar N, Khwairakpam G et al. Community Network for Empowerment (CoNE). Completing the Hepatitis C screening, diagnosis and treatment cascade. Project Report 2018
64. Wong VW, Wong GL, Chim AM, et al. Targeted hepatitis C screening among ex-injection drug users in the community. J Gastroenterol Hepatol. 2014 Jan;29(1):116-20. doi: 10.1111/jgh.12355.
65. Masson CL, Delucchi KL, McKnight C, et al. A Randomized Trial of a Hepatitis Care Coordination Model in Methadone Maintenance Treatment. American Journal of Public Health. 2013;103(10):e81-e88. doi:10.2105/AJPH.2013.301458.
66. Islam, M, Top L, Conigrave KM, et al. Linkage into specialist hepatitis C treatment services of injecting drug users attending a needle syringe program-based primary healthcare centre. J Subst Abuse Treat. 2012 Dec;43(4):440-5. doi: 10.1016/j.jsat.2012.07.007.
67. Martinez AD, Dimova R, Marks KM, et al. Integrated internist — addiction medicine — hepatology model for hepatitis C management for individuals on methadone maintenance. Journal of viral hepatitis. 2012;19(1):47-54. doi:10.1111/j.1365-2893.2010.01411.x.
68. Surey J, Gibbons J, Francis M, et al. HepFriend: Peer Support and Community Engagement in Underserved Populations with Hepatitis C in the UK, Ireland, Romania and Spain as Part of the HepCare Programme. 7th International Symposium on Hepatitis Care in Substance Users, Cascais Portugal, 19-21 September 2018.
69. Anagnostou O, Kranidioti C, Micha K, et al. Is the availability of direct acting antiviral agents (DDAs) enough to treat chronic hepatitis C (CHC) and achieve HCV elimination among people who use drugs (PWUD)? What do the real world data suggest? 7th International Symposium on Hepatitis Care in Substance Users, Cascais Portugal, 19-21 September 2018.
70. Foroghi L, Campogiani L, Teti E, et al. Preliminary results about HCV Care Cascade among PWID in Rome: "Una regione senza la C" project. 7th International Symposium on Hepatitis Care in Substance Users, Cascais Portugal, 19-21 September 2018.
71. Dominguez S, Bachelard A, Avril E, et al. Strengthening patient's pathways for a better hepatitis C (HCV) cascade care in most vulnerable populations: the HCV "parcours" project in Ile de France (IDF). 7th International Symposium on Hepatitis Care in Substance Users, Cascais Portugal, 19-21 September 2018.
72. Losikoff P, Chiodo L, Martin S, et al. Providing Access to Hepatitis C Treatment Improves Adherence to Addiction Treatment in an Outpatient Addiction Treatment Center. 7th International Symposium on Hepatitis Care in Substance Users, Cascais Portugal, 19-21 September 2018.
73. Bielen R, Dercon E, Koc O, et al. Hepatitis C nurse as a case manager in people who inject drugs. 7th International Symposium on Hepatitis Care in Substance Users, Cascais Portugal, 19-21 September 2018.
74. Fuchs D, Craddock S, Rodger D, et al. Expanding Access to Hepatitis C Care in Community and Correctional Settings via Non-Traditional Models in Sasktchewan, Canada. 7th International Symposium on Hepatitis Care in Substance Users, Cascais Portugal, 19-21 September 2018.
75. Harney BL, Whitton B, Lim C, et al. Evaluation of an integrated nurse model of care providing hepatitis C treatment to people attending homeless services in Melbourne, Australia. 7th International Symposium on Hepatitis Care in Substance Users, Cascais Portugal, 19-21 September 2018.
76. Antonini TM, Deschenau A, Le Pape S, et al. Feasibility of a global out of wall assessment of hepatitis C liver disease in a drug service. 7th International Symposium on Hepatitis Care in Substance Users, Cascais Portugal, 19-21 September 2018.
77. Holeska J, Alimohammadi A, Truong D, et al. Community Outreach Events - Engaging the Disengaged. 7th International Symposium on Hepatitis Care in Substance Users, Cascais Portugal, 19-21 September 2018.

78. Swan D, O'Connor E, McCombe G, et al. Integrating Hepatitis C care for at risk groups; Findings from a Multi-centre Observational Study in Primary and Community Care. 7th International Symposium on Hepatitis Care in Substance Users, Cascais Portugal, 19-21 September 2018.
79. Alimohammadi A, Holeska J, Yung R, et al. Real-life cohorts and the impact of losses to follow-up (LTFU) on HCV sustained virologic response (SVR) rates. 7th International Symposium on Hepatitis Care in Substance Users, Cascais Portugal, 19-21 September 2018.
80. de Gijsel D, Fleischer N. Low Hepatitis C Treatment Rates among Patients Screened as Inpatients at a Rural Academic Medical Center. 7th International Symposium on Hepatitis Care in Substance Users, Cascais Portugal, 19-21 September 2018.
81. Ucbilek E, Sahin Horasan E. New directly acting antiviral therapy of HCV infection people who injected drugs: real life experience from Turkey. 7th International Symposium on Hepatitis Care in Substance Users, Cascais Portugal, 19-21 September 2018.
82. Morey S, Hamood A, Valappil M, et al. A universal offer of blood borne virus testing substantially increases diagnosis and treatment of HCV in prisons. Poster Abstract, The International Liver Congress, April 2018.
83. Lloyd AR, Clegg J, Lange J, et al. Safety and effectiveness of a nurse-led outreach program for assessment and treatment of chronic hepatitis C in the custodial setting. Clinical Infectious Diseases, Volume 56, Issue 8, 15 April 2013, Pages 1078–1084. Epub 2013 Jan 29. doi: 10.1093/cid/cis1202.
84. Fridriksdottir RH, Fridjonsdottir H, Alexiusdottir K, et al. Marked reduction of hepatitis C prevalence in the prison setting during 2nd year of TraP HepC (Treatment as Prevention for Hepatitis C) program in Iceland. 7th International Symposium on Hepatitis Care in Substance Users, Cascais Portugal, 19-21 September 2018.
85. Bachelard A, Dullioust A, Bambe A, et al. Special concern and strategy for optimizing cascade care among prisoners. The HCV "parcours" project in Ile de France (IDF). 7th International Symposium on Hepatitis Care in Substance Users, Cascais Portugal, 19-21 September 2018.
86. Levy M, Harkness B, Evans R, et al. An on-site primary care approach delivers rapid reduction of hepatitis C in prison - Canberra, Australia. 7th International Symposium on Hepatitis Care in Substance Users, Cascais Portugal, 19-21 September 2018.
87. Farley J, Jabar A, Farley J-R, Hakobyan V. Chronic Hepatitis C Eradication Model Through Primary Care: Treating HCV In Prisons And The Community Continuum Of Care. 7th International Symposium on Hepatitis Care in Substance Users, Cascais Portugal, 19-21 September 2018.
88. Islam Z, Alliance for Public Health. Catalyst for Change, Viral Hepatitis Control Program, Country Model Ukraine. Personal communication. October 2018.
89. Papaluca T, McDonald L, Gibson A, et al. Outcomes of treatment for hepatitis C in prisoners using nurse-led, statewide model of care. Journal of Hepatology, Volume 70, Issue 5, 839-846. May 2019.
90. Cuadrado A, LLerena S, Cobo C, et al. Microenvironment Eradication of Hepatitis C: A Novel Treatment Paradigm. Am J Gastroenterol. 2018 Nov; 113(11): 1639-1648.
91. Hawks L, Norton BL, Cunningham CO, Fox AD. The Hepatitis C virus treatment cascade at an urban post incarceration transitions clinic. J Viral Hepat, 23: 473-478. Epub 2016 Feb 9 doi:10.1111/jvh.12512
92. Mohamed Z. Improving HCV treatment uptake in prisons: breaking the 60 day barrier. 7th International Symposium on Hepatitis Care in Substance Users, Cascais Portugal, 19-21 September 2018.
93. Boonwaat L, Haber PS, Levy MH, Lloyd AR. Establishment of a successful assessment and treatment service for Australian prison inmates with chronic hepatitis C. Med J Aust 2010; 192 (9): 496-500.
94. Jack K, Thomson B, Linsey P, et al. C-INSIDE: Outcomes of an opt-out Hepatitis C testing policy in the East Midlands prison estate. Poster Abstract, The International Liver Congress, April 2018.
95. Winter RJ, White B, Kinner SA, et al. A nurse-led intervention improved blood-borne virus testing and vaccination in Victorian prisons. Australian and New Zealand Journal of Public Health. Volume 40, Issue 6 December 2016 Pages 592-594. Epub 2016 October 23

96. Schoenbachler BT, Smith BD, Seña AC, et al. Hepatitis C Virus Testing and Linkage to Care in North Carolina and South Carolina Jails, 2012–2014. Public Health Reports Volume: 131 issue: 2_suppl, page(s): 98-104. Epub: 2016 May 1. doi.org/10.1177/00333549161310S215
97. Seña AC, Willis SJ, Hilton A, et al. Efforts at the Frontlines: Implementing a Hepatitis C Testing and Linkage-to-Care Program at the Local Public Health Level. Public Health Reports. 2016;131(Suppl 2):57-64.
98. Beckwith CG, Kurth AE, Bazerman LB, et al. A pilot study of rapid hepatitis C virus testing in Rhode Island department of corrections. J Public Health (Oxf). 2016 Mar;38(1):130-7. doi: 10.1093/pubmed/fdv023.
99. Cocoros N, Nettle E, Church D, et al. Screening for Hepatitis C as a Prevention Enhancement (SHAPE) for HIV: An Integration Pilot Initiative in a Massachusetts County Correctional Facility. Public Health Reports. 2014;129(Suppl 1):5-11.
100. Perrett SE. Prisoner health: assessing a nurse-led hepatitis C testing clinic. Br J Nurs. 2011 May 27-Jun 9;20(10):611-4. DOI: 10.12968/bjon.2011.20.10.611
101. Doyle JS. Hepatitis C treatment success in primary and tertiary care among people with HCV/HIV coinfection. Poster Abstract, International Liver Conference, Paris, 2018
102. Cachay ER, Hill L, Ballard C, et al. Increasing Hepatitis C treatment uptake among HIV-infected patients using an HIV primary care model. AIDS Res Ther. 2013 Mar 28;10(1):9. doi: 10.1186/1742-6405-10-9. doi: 10.1186/1742-6405-10-9
103. Traeger M, Pedrana A, Draper B, et al. Hepatitis C testing, treatment uptake and sustained virologic response among gay and bisexual men with hepatitis C and HIV co infection in Melbourne, Australia. 7th International Symposium on Hepatitis Care in Substance Users, Cascais Portugal, 19-21 September 2018.
104. Nguyen A, Medicines San Frontieres (MSF). Personal Communication. 21 November 2018.
105. Ford MM, Jordan AE, Johnson N, et al. Check Hep C: A Community-Based Approach to Hepatitis C Diagnosis and Linkage to Care in High-Risk Populations. J Public Health Manag Pract. 2018 Jan/Feb;24(1):41-48.
106. Belperio PS, Chartrier M, Ross DB, et al. Curing Hepatitis C Virus Infection: Best Practices From the U.S. Department of Veterans Affairs. Ann Intern Med. 2017 Oct 3;167(7):499-504. doi: 10.7326/M17-1073.
107. Capileno YA, Van den Bergh R, Donchunk D, et al, Management of chronic Hepatitis C at a primary health clinic in the high-burden context of Karachi, Pakistan. doi.org/10.1371/journal.pone.0175562
108. Mera J, Vellozzi C, Hariri S, et al. Identification and clinical management of persons with Chronic Hepatitis C virus infection- Cherokee Nation, 2012-2015. MMWR Morb Mortal Wkly Rep 2016; 65: 461-466.
109. Jayasekera CR, Perumpail RB, Chao DT, et al. Task-Shifting: An Approach to Decentralized Hepatitis C Treatment in Medically Underserved Areas. Digestive Diseases and Sciences December 2015, Volume 60, Issue 12, pp 3552–3557. DOI: 10.1007/s10620-015-3911-6
110. Kattakuzhy S, Gross C, Emmanuel B, et al. Expansion of Treatment for Hepatitis C Virus Infection by Task Shifting to Community Based Non Specialists Providers: A non-randomized trial. Ann Intern Med. 2017 Sep 5;167(5):311-318. doi: 10.7326/M17-0118.
111. Dhiman R. Treatment of Hepatitis C patients in a public health care setting with sofosbuvir based treatment regimens: Efficacy and safety in real life Punjab, India. AASLD Liver Learning. Oct 22, 2017; 195072
112. Cooper CL, Hatashita H, Corsi DJ, et al. Direct Acting Antiviral Therapy Outcomes in Canadian Chronic Hepatitis C Telemedicine Patients. Annals of Hepatology Nov-Dec, Vol 16 No 6 2017 874 880
113. Lasser KE, Heinz A, Battisti L, et al. A Hepatitis C Treatment Program Based in a Safety-Net Hospital Patient-Centered Medical Home. Ann Fam Med. 2017 May;15(3):258-261. doi: 10.1370/afm.2069.

114. Baker D, Alavi M, Erratt A, et al. Delivery of Treatment for Hepatitis C virus infection in the primary care setting. Eur J Gastroenterol Hepatol. 2014 Sep;26(9):1003-9.
115. Nazareth S, Kontorinis N, Muwanwella H, et al, Successful treatment of patients with hepatitis C in rural and remote Western Australia via telehealth. J Telemed Telecare. 2013 Feb;19(2):101-6. doi: 10.1258/jtt.2012.120612
116. Arora S, Thornton K, Murata G, et al. Outcomes of Treatment for Hepatitis C Virus Infection by Primary care providers. N Engl J Med 2011; 364:2199-2207 doi: 10.1056/NEJMoa1009370
117. Myers R, Cooper C, Sherman D, et al. Outcomes of chronic hepatitis C therapy in patients treated in community versus academic centres in Canada: Final results of APPROACH (A Prospective study of Peginterferon alfa-2a and Ribavirin at Academic and Community Centres in Canada). Can J Gastroenterol. 2011 Sep; 25(9): 503–510.
118. Rossaro L, Aoki C, Yuk J, et al. The Evaluation of Patients with Hepatitis C Living in Rural California via Telemedicine. Telemed J E Health. 2008 Dec; 14(10): 1127–1129. doi: 10.1089/tmj.2008.0029
119. Hill WD, Butt G, Alvarez M, et al. Capacity enhancement of hepatitis C virus treatment through integrated, community-based care. Can J Gastroenterol. 2008 Jan; 22(1): 27–32.
120. Jack K, Barnett J, Holiday A, et al. Hepatitis C therapy at home: a hospital and home care partnership. Br J Nurs. 2013 May 9-22;22(9):518-23.
121. Shiha G, Metwally AM, Soliman R, et al. Educate, Test and Treat: A community model towards the elimination of Hepatitis C in Egypt. Lancet Gastro Hep. 2018 Nov;3(11): 778-789.
122. Anartati A, Kosahih R, Budiman A, et al. Scale up of the National HCV screening and treatment program in Indonesia: data from Jakarta, West Java and Central Java. Poster Abstract, International Liver Conference, Paris, 2018
123. Teply R, Mukherjee S, Goodman M, et al. Impact of a Hepatitis C Virus Electronic Medical Record Screening Alert for Baby Boomers. Poster Abstract, The International Liver Congress, April 2018.
124. Ivantes CA, El Tawil FB, Souza DR, et al. Report of well succeeded experience in treatment of viral hepatitis by training general physicians from basic health units and by creation of a multidisciplinary team. Poster Abstract, World Hepatitis Summit. São Paulo, Brazil. November 1-3 2017.
125. Smyth D, Francheville JW, Rankin R, et al. Early successes in an open access, provincially funded hepatitis C treatment program in Prince Edward Island. Ann Hepatol. 2017 Sep-Oct;16(5):749-758. doi: 10.5604/01.3001.0010.2757.
126. Coyle C, Kwakwa H, Viner K. Integrating Routine HCV Testing in Primary Care: Lessons Learned from Five Federally Qualified Health Centers in Philadelphia, Pennsylvania, 2012-2014. Public Health Rep. 2016 May-Jun;131 Suppl 2:65-73. doi: 10.1177/00333549161310S211.
127. Falade-Nwulia O, Mehta SH, Lasola J, et al. Public health clinic-based hepatitis C testing and linkage to care in Baltimore. J Viral Hepat. 2016 May;23(5):366-74. doi: 10.1111/jvh.12507.
128. Trooskin SB, Poceta J, Towey CM, et al. Results from a Geographically Focused, Community-Based HCV Screening, Linkage-to-Care and Patient Navigation Program. J Gen Intern Med. 2015 Jul;30(7):950-7. doi: 10.1007/s11606-015-3209-6.
129. Litwin AH, Smith BD, Drainoni ML, et al. Primary care-based interventions are associated with increases in hepatitis C virus testing for patients at risk. Dig Liver Dis. 2012 Jun;44(6):497-503. doi: 10.1016/j.dld.2011.12.014.
130. Luma H, Eloumou S, Noah D, et al. Hepatitis C Continuum of Care in a Treatment Center in Sub-Saharan Africa, Journal of Clinical and Experimental Hepatology. doi.org/10.1016/j.jceh.2018.01.001
131. Carnaúba Junior D, Tenore SB, Sasaki M, et al. Efficacy and safety of oral direct acting antivirals for the treatment of chronic hepatitis C virus infection: Brazilian experience in two public outpatient clinics in São Paulo. Poster Abstract, World Hepatitis Summit. São Paulo, Brazil. November 1-3 2017

132. Qureshi H, Muhammad K, Mahmood H, et al. The report of a collaboration of a civil society organization and private gastroenterologist in diagnosis and treatment of patients with HCV infection. Poster Abstract, World Hepatitis Summit. São Paulo, Brazil. November 1-3 2017.
133. Iwamoto M, Dousset JP, Kimchamroeun S, et al. Identifying the optimal care model for HCV care in Cambodia, and overcoming barriers to decentralization and scale-up: MSF pilot program. Poster Abstract, World Hepatitis Summit. São Paulo, Brazil. November 1-3 2017.
134. Levin JM, Dabirshahsahebi S, Bauer M, Huckins, E. Retrospective analysis of hepatitis C infected patients treated through an integrated care model. World J Gastroenterol. 2016 Oct 14;22(38):8558-8567. DOI: 10.3748/wjg.v22.i38.8558
135. Gallach M, Vergara M, Miquel M, et al. Effects of a multidisciplinary approach on the effectiveness of antiviral treatment for chronic Hepatitis C. Ann Hepatol. 2016 Jul-Aug;15(4):524-31.
136. Ahmed I, Habibi A, Iqbal J, et al. Improving outcome in hepatitis C management- a need for dedicated multi-disciplinary service to improve compliance. Journal of Gastroenterology and Hepatology Research. Vol 2, No 8 (2013)
137. Goel A, Sanchez J, Paulino L, et al. A systematic model improves hepatitis C virus birth cohort screening in hospital-based primary care. J Viral Hepat. 2017 Jun;24(6):477-485. doi: 10.1111/jvh.12669.
138. Malhotra P, Malhotra N, Malhotra V, et al. Jeevan Rekha: Counter for Hepatitis C in Haryana. Adv Res Gastroentero Hepatol. 2016; 1(3): 555561. doi: 10.19080/ARGH.2016.01.555561
139. Turner BJ, Taylor BS, Hanson JT, et al. Implementing Hospital Based Baby Boomner Hepatitis C Virus Screening and linkage to care: Strategies, results, and costs. J Hosp Med. 2015 Aug;10(8):510-6. doi: 10.1002/jhm.2376
140. Woodrell C, Weiss J, Branch A, et al. Primary Care-Based Hepatitis C Treatment Outcomes With First-Generation Direct-Acting Agents. J Addict Med. 2015 Oct; 9(5): 405–410.
141. Kuo YH, Chen PF, Wang JH, et al. Comparison Stratagems of Post-Screening Management of Anti-HCV-Positive Community Residents: Simple Notification, Active Referral, or Accessible Medical Care. PLoS One. 2015 May 13;10(5):e0126031.
